# Synthesis of medronic acid monoesters and their purification by high-performance countercurrent chromatography or by hydroxyapatite

**DOI:** 10.3762/bjoc.12.204

**Published:** 2016-10-07

**Authors:** Elina Puljula, Jouko Vepsäläinen, Petri A Turhanen

**Affiliations:** 1 School of Pharmacy, University of Eastern Finland, Biocenter Kuopio, P.O.Box 1627, FI-70211 Kuopio, Finland

**Keywords:** bisphosphonate, bisphosphonate monoester, high performance counter current chromatography, hydroxyapatite, medronic acid

## Abstract

We achieved the synthesis of important medronic acid monoalkyl esters via the dealkylation of mixed trimethyl monoalkyl esters of medronic acid. Two methods were developed for the purification of medronic acid monoesters: 1) small scale (10–20 mg) purification by using hydroxyapatite and 2) large scale (tested up to 140 mg) purification by high-performance countercurrent chromatography (HPCCC).

## Introduction

**Figure 1:**
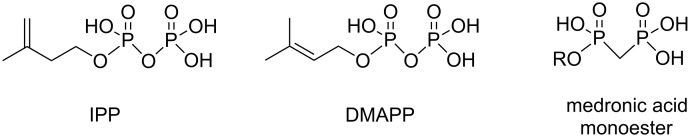
Structures of isopentenyl pyrophosphate (IPP), dimethylallyl pyrophosphate (DMAPP) and the general structure of medronic acid monoesters.

Bisphosphonates (BPs) have been used for decades in the treatment of bone-related diseases as well as for bone imaging. Nevertheless, several recent reports have demonstrated their versatility extends beyond their conventional uses, making them still highly attractive compounds [1–5]. Monoesters of medronic acid are structural analogues of isopentenyl pyrophosphate (IPP) and dimethylallyl pyrophosphate (DMAPP) (Figure 1), both common and important metabolites of the mevalonate pathway [6]. In addition, IPP is known to be able to stimulate gamma delta (γδ) T-cells [7] and BP monoesters have been demonstrated to exert effects on γδ T-cells as well [8–9]. Activators of γδ T-cells have been claimed to be potentially useful for cancer immunotherapy [10]. Pyrophosphates contain a P–O–P structural motif and are thus unstable against chemical and enzymatic hydrolysis, whereas BPs have a more stable P–C–P structure (see general structure in Figure 1). The increased stability means that these kinds of molecules can be used as enzyme inhibitors [11–12].

Countercurrent chromatography (CCC) is an old invention but recently this technique has seen great advances, leading to the appearance of bench top CCC methodology instruments in many laboratories. One of the latest developments in this field is the HPCCC instrument; this achieves improved peak resolution within a reasonable time scale [13]. HPCCC relies on the combination of a biphasic immiscible solvent system and a high centrifugal force field. The centrifugal force immobilizes one of the phases in the coiled column (stationary phase), while the remaining phase can be pumped through the column (mobile phase). The rotation and the coiled structure of the column induce multiple sequential extractions between these two phases and thus the compounds are eluted from the column according to their partition coefficients. Most of the biphasic solvent systems contain different combinations of hexane, ethyl acetate, methanol (or butanol) and water (HEMWat). The majority of the applications developed for HPCCC focus on the separation and purification processes for natural products [14–15] and only a few reports describe the purification of synthetic products [16–18]. In many purification applications CCC is coupled with HPLC to achieve optimal results, but it has also been proposed that CCC could be considered as an alternative method to RP-HPLC [19]. As far as we are aware, CCC instruments have not been previously used for the purification of BPs.

Despite the potential wide-range uses of medronic acid monoesters, there are only a few publications describing alternative protocols for their synthesis. This is most likely because, in general, BP monoesters are challenging to prepare. The synthesis of monoesters has been described previously by using tris(tetra-*n*-butylammonium) methylenediphosphonate and alkyl halides [20] or tosylated alcohols [8] with the desired alkyl group. However, the purification process is laborious and includes several extractions and chromatographic procedures. Furthermore, most of the published reports of medronic acid monoesters do not contain any NMR data, which could prove the achieved degree of purity. Some other methods have also been used for the synthesis of nucleoside methylene-BPs which could structurally be considered as medronic acid monoesters [21–22].

Interestingly, there are only a few publications describing the synthesis of medronic acid monoesters via dealkylation of medronic acid mixed tetraesters [22]. The synthesis of triesters [23] and symmetrical diesters [24] from medronic acid tetraesters has been achieved previously by using tertiary and secondary amines, for example. Unfortunately the same strategy cannot be applied to the synthesis of monoesters. Silylhalides are routinely used for dealkylation [25] of BP esters. Moreover, since the dealkylation reaction by silylation favors sterically less hindered methyl esters over other esters [26], we propose that trimethyl monoalkyl esters could offer a simple route for the synthesis of medronic acid monoesters.

**Scheme 1:**
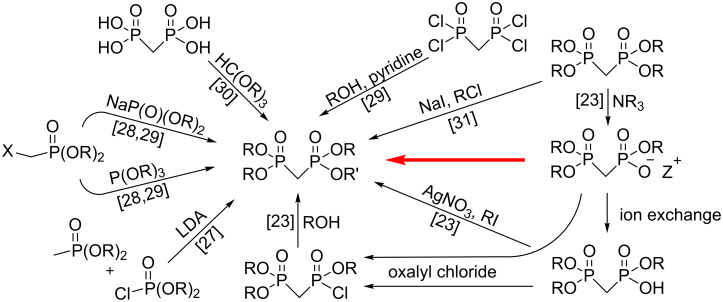
Synthetic strategies for mixed tetraesters of medronic acid. The method described in this paper is marked with a red arrow.

Several methods have been utilized for the production of mixed tetraesters of medronic acid (Scheme 1). One option is to build the tetraester from monophosphonates and phosphites with the desired alkyl groups. The most commonly used reactions are Michaelis–Becker, Michaelis–Arbuzov and carbanion methods. From these three the carbanion method has been proposed to be the most convenient approach to produce mixed tetraesters, but it requires extremely dry conditions because of the use of lithium diisopropylamide (LDA) [27].

Methylenebis(phosphonic dichloride) [29,32] and medronic acid [30] can also be used as the starting material for the synthesis of mixed tetraesters. If the starting point is a tetraester of medronic acid, several routes can be taken, for example transesterification [31]. In addition, after dealkylation, the corresponding triester can be esterified via chlorination of the triester [33] or directly by using alkylhalides. Many of these strategies are sensitive to moisture and require dry conditions, or alternatively lead to the formation of mixtures of different mixed tetraesters of medronic acid. Additionally, we encountered one previously unreported problem in the chlorination reaction of trimethyl methylene-BP with oxalyl chloride, i.e., we observed that the triester readily formed a dimer with itself during the chlorination reaction according to the ^1^H and ^31^P NMR spectra. Dimerization has been previously observed with clodronic acid trimethyl ester but not with medronic acid trimethyl ester [34]. In our case the dimerization led to low and unpredictable yields and therefore it was decided to develop an alternative method for the synthesis of mixed tetraesters.

Here we describe a convenient method with good yields for the synthesis of mixed tetraesters as the starting material for the synthesis of more important monoesters of medronic acid and a simple HPCCC-based method for the purification of the prepared monoesters. In addition, a small-scale purification method for medronic acid monoesters by using hydroxyapatite is introduced.

## Results and Discussion

**Scheme 2:**
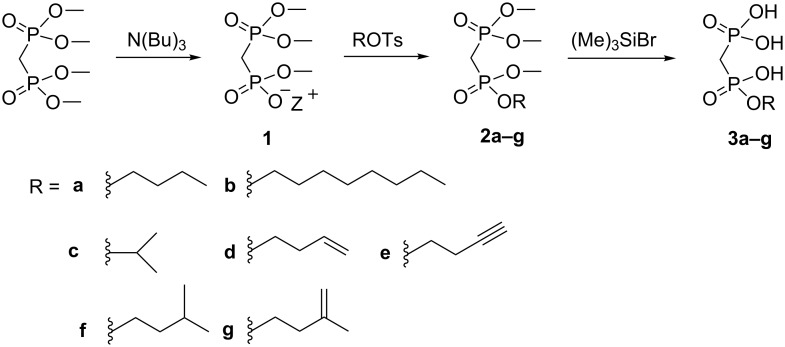
Synthesis of medronic acid monoesters.

The synthesis of mixed tetraesters from the tetraalkylammonium salt of medronic acid trimethylester and alkyl tosylate utilizes a similar strategy used previously for the synthesis of monoesters (Scheme 2). The starting point for the synthesis was tetramethyl methylene-BP, from which one of the methyl groups was removed by selective demethylation with tributylamine. The resulting mono *N*,*N*,*N*,tributyl-*N*-methylammonium salt of the triester was then refluxed with the desired alkyl tosylate in acetonitrile. The resulting mixed tetraesters **2a–g** were purified by column chromatography with relatively high yields (60–78%).

The three methyl ester groups were removed with bromotrimethylsilane at low temperature (<0 °C). The removal of the methylesters by silylation was found not to be fully specific and it led always to the silylation of small amounts of the alkyl ester, despite the low silylation temperature. This resulted in mixtures of the desired monoalkylester and medronic acid (5–20%) after the hydrolysis of silyl esters. It was evident that a suitable purification method was needed in order to obtain monoesters with sufficient purity.

Two strategies were tested for the purification of monoalkyl esters. The first approach was based on the fact that medronic acid is more likely than its monoesters to bind to hydroxyapatite [35]. The crude monoesters were first converted into their disodium salts and then dissolved in a small volume of dry methanol. The desired amount of hydroxyapatite was added to the solution and the mixture was stirred for 20 minutes, filtered and the solvent evaporated. The medronic acid was bound to the hydroxyapatite, leaving the desired monoester in the solution. The only drawback of this method was that it could only be used in small scale (10–20 mg) purifications. The use of larger amounts led to the partial dissolution of phosphate from the hydroxyapatite and, according to the ^31^P NMR spectra, mixtures of the monoester and phosphate were obtained.

For larger scale purifications, an HPCCC-based method was developed. The HEMWat solvent system number one (Dynamic Extractions) containing water and *n*-butanol was found to be most suitable for the purification of the monoesters. The separation by HPCCC is based on the different solubilities of the compounds being separated between two liquid phases. It was found that the acidic form of the monoester achieved a better separation from medronic acid when compared to the disodium salt forms of the monoester and medronic acid (medronate). However, the isopentenyl monoester **3g** proved to be unstable in aqueous solutions in its acidic form. For this reason it was converted into its monosodium salt instantly after the hydrolysis of the silylesters. The purification in the sodium salt form resulted in a lower yield (31%), as fewer of the fractions contained only the pure monoester because of peak overlapping with medronate. A low yield (29%) was obtained also with compound **3d** as it was converted into its disodium salt before the purification.

## Conclusion

Mixed tetraesters of medronic acid were prepared with relatively high yields (60–78%) and used as starting material for the synthesis of monoesters of medronic acid. A series of medronic acid monoesters were synthesized by using a silylation and desilylation procedure of mixed tetraesters. Two methods were developed for the purification of monoesters, the first one based on the superior hydroxyapatite binding of medronic acid and the second approach exploited HPCCC and different partitioning coefficients of monoesters and the medronic acid. The usefulness of HPCCC in the purification of monoesters was demonstrated with 29–64% yields. Additionally, according to a recent SciFinder search, compounds **2a**,**b**, **2d–g**, **3a** and **3c–e** have not been reported earlier in the literature.

## Supporting Information

File 1 Full experimental details and characterization data for all compounds.

File 2 ^1^H, ^13^C, and ^31^P NMR spectra and an example of HPCCC chromatogram.
