# Supplementary material for: Synthesis of medronic acid monoesters and their purification by high-performance countercurrent chromatography or by hydroxyapatite
Source: Beilstein J Org Chem. 2016 Oct 7;12:2145–9. doi: 10.3762/bjoc.12.204 (PMC5082484; doi:10.3762/bjoc.12.204)
Supplement: File 1 — Full experimental details and characterization data for all compounds. [file Beilstein_J_Org_Chem-12-2145-s001.pdf]

Supporting Information File 1

for

Synthesis of medronic acid monoesters and their purification by high-performance countercurrent chromatography or by hydroxyapatite

Elina Puljula*, Jouko Vepsäläinen and Petri A. Turhanen

Address: School of Pharmacy, University of Eastern Finland, Biocenter Kuopio, P.O.Box 1627, FI-70211 Kuopio, Finland

Email: Elina Puljula - elina.puljula@uef.fi

*Corresponding author

Full experimental details and characterization data for all compounds

Experimental procedures

General. All chemicals were purchased from commercial sources and were used without further purification. In any moisture sensitive reactions, the solvents were distilled prior to their use. Column chromatography was performed on silica gel (70–230 mesh). Alkyl tosylates were prepared from tosyl chloride and the respective alcohol, using pyridine as the base (as an example, the detailed procedure for the preparation of isopentenyl tosylate can be found in a recent report published by

Weisell et al. [1]). Compounds **2c** [2], **3b** [3], **3f** [4] and **3g** [5] have been reported in the literature previously. ^{1}H, ^{31}P, and ^{13}C NMR spectra were recorded on a 500 MHz spectrometer operating at 500.1, 202.5, and 125.8 MHz or on a 600 MHz spectrometer operating at 600.2, 243.0, and 150.9 MHz. Tetramethylsilane (TMS) and trimethylsilylpropionic acid sodium salt (TSP) were used as internal standards for ^{1}H and ^{13}C measurements and 85% H_3PO_4 as an external standard for ^{31}P measurements. A drop of sodium deuteroxide (NaOD) solution (6 M in D_2O) was added to all samples made in D_2O to ensure needed sharpness of ^{31}P signals. An exception was made for compound **3e**; ^{1}H and ^{13}C spectra were measured without NaOD to avoid the exchange of the alkyne proton to deuterium. The purification of monoesters was performed on an HPCCC instrument (Spectrum, Dynamic Extractions, Slough, UK). The analytical (22.5 mL) coils and HEMWat solvent system number one (water and 1-butanol, 1:1) were used in all separations. The reversed phase mode was applied, in other words, the upper phase of the solvent system was used as the stationary phase and the lower phase as the mobile phase. Chromatography was monitored by UV-detection at 200, 220 and 254 nm. High resolution mass spectra (HRMS) were recorded on a q-TOF or TOF mass spectrometer with ESI source.

General procedure: the synthesis of trimethyl monoalkyl esters. Equimolar amounts of **1** and the appropriate alkyl tosylate were refluxed in acetonitrile (30–40 mL). The reaction mixture was evaporated to dryness, 50 mL of ether were added and the mixture was kept in a freezer until a white solid was formed. The white solids were filtered off, and the ethereal phase was evaporated to dryness, leaving the crude product. The crude product was purified by silica column chromatography, with

a mixture of ethyl acetate and methanol as the eluent and TLC staining being done with potassium permanganate.

General procedure: the silylation of trimethyl monoalkyl esters. The reaction was performed under argon atmosphere. The ester was dissolved in dry acetonitrile and the mixture was cooled to −5 to 0 °C and subsequently 3.5–5 equiv of trimethylsilylbromide were added. The reaction temperature was kept at approximately −5 °C and the reaction was monitored by ^{1}H NMR (disappearance of methyl signals) and stopped after 2–5 hours by evaporation to dryness. The silyl esters were hydrolyzed by mixing the crude product with methanol for 15 minutes. Finally, the methanol was evaporated and the crude product was purified by HPCCC.

General procedure: the purification of monoalkyl esters by HPCCC with HEMWat solvent system 1. Samples were dissolved in 1 mL of the lower phase prior to the separation. Stationary phase retention was typically 33%. The flow rate was kept at 0.5 mL/min until the dynamic equilibrium was reached and for the first 10 minutes of the separation. After 10 minutes the flow was set to 1 mL/min. The fraction volume was 0.5–1.0 mL. A typical separation lasted for 40 minutes.

Procedure example: the purification of compound 3g by hydroxyapatite. The monoester **3g** (10 mg) as its disodium salt form containing 10% of medronate as an impurity was dissolved in dry methanol (12 mL). Hydroxyapatite (50 mg) was added and the mixture was stirred for 20 minutes at room temperature. Hydroxyapatite was filtered off and the mixture was evaporated to dryness, giving 7 mg of the pure product. The amount of hydroxyapatite was estimated prior to the experiment by determining the amount of medronate that the hydroxyapatite could bind [6]. The

hydroxyapatite used in the experiment could bind 86 nmol of medronate per 1 mg of hydroxyapatite.

[(Dimethoxyphosphino)methyl]phosphonic acid monomethyl ester *N,N,N*-tributyl-*N*-methyl ammonium salt (**1**) was prepared by following the procedure described by Vepsäläinen et al. [7]. from tetramethyl methylenebisphosphonate and tributylamine. Equimolar amounts of tetramethyl methylenebisphosphonate (3.0 g, 0.0129 mol) and tributylamine (2.40 g, 0.0129 mol) were refluxed in acetonitrile for seven days. The reaction mixture was evaporated to dryness and washed with a mixture of ether and *n*-hexane (60 mL, 1:1) to remove traces of unreacted tetramethyl methylenebisphosphonate. Compound **1** was obtained as a viscous oil. Yield 4.38 g (81%). ^{1}H NMR (600 MHz, $CDCl_3$) δ 3.77 (d, J = 11.2Hz, 6H), 3.61 (d, J = 10.8Hz, 3H), 3.44-3.39 (m, 6H), 3.30 (s, 3H), 2.31 (dd, J = 20.8Hz, 18.3Hz, 2H), 1.69-1.61 (m, 6H), 1.49-1.40 (m, 6H), 1.04-0.96 (m, 9H); ^{13}C NMR (151 MHz, $CDCl_3$) δ 61.0, 52.5 (d, J = 6.3Hz), 51.8 (d, J = 6.0Hz), 48.9, 24.6 (dd, J = 130.7Hz, 117Hz), 24.4, 19.7, 13.7; ^{31}P NMR (202 MHz, $CDCl_3$) δ 29.91 (d, J = 7.0Hz), 8.66 (d, J = 7.0Hz). HRMS (ESI-qTOF) m/z: $[M-H]^-$ Calcd for $C_4H_{11}O_6P_2$ 217.0031; Found 217.0043.

Dimethyl {[butoxy(methoxy)phosphoryl]methyl}phosphonate (**2a**) was prepared by following the general procedure described earlier in this paper from ammonium salt **1** (530 mg, 1.27 mmol) and butyl tosylate (290 mg, 1.27 mmol). The purification by silica column chromatography with ethyl acetate:methanol (8:2, R_f = 0.6) as the eluent gave the pure product as a colorless oil. Yield 271 mg (78%). ^{1}H NMR (500 MHz, $CDCl_3$) δ 4.19-4.05 (m, 2H), 3.82 (d, J = 11.4 Hz, 3H), 3.82 (d, J = 11.4 Hz, 6H), 2.46 (t, J = 21.1Hz, 2H), 1.73-1.64 (m, 2H), 1.47-1.37 (m, 2H), 0.94 (t, J = 7.4

Hz, 3H); ^{13}C NMR (126 MHz, $CDCl_3$) δ 66.5 (d, J = 6.7Hz), 53.2 (d, J = 6.4Hz), 53.1 (d, J = 6.4Hz), 32.5 (d, J = 6.4Hz), 24.2 (t, J = 136.8Hz), 18.7, 13.6; ^{31}P NMR (202 MHz, $CDCl_3$) δ 23.37 (d, J = 6.1Hz), 21.76 (d, J = 6.1Hz). HRMS (ESI-qTOF) m/z: $[M+H]^+$ Calcd for $C_8H_{21}O_6P_2$ 275.0813; Found 275.0816.

Dimethyl {[octyloxy(methoxy)phosphoryl]methyl}phosphonate (**2b**) was prepared by following the general procedure described earlier in this paper from ammonium salt **1** (530 mg, 1.27 mmol) and octyl tosylate (361 mg, 1.27 mmol). The purification by silica column chromatography with ethyl acetate:methanol (9:1, R_f = 0.5) as the eluent gave the pure product as a colorless oil. Yield 289 mg (69%). ^{1}H NMR (500 MHz, $CDCl_3$) δ 4.16-4.07 (m, 2H), 3.82 (d, J = 11.3Hz, 3H), 3.81 (d, J = 11.3Hz, 6H), 2.46 (t, 21.4Hz, 2H), 1.74-1.65 (m, 2H), 1.42-1.21 (m, 10H), 0.94-0.84 (m, 3H); ^{13}C NMR (126 MHz, $CDCl_3$) δ 66.9 (d, J = 6.8Hz), 53.2 (d, J = 6.4Hz), 53.1 (d, J = 6.4Hz), 31.8, 30.5 (d, J = 6.05Hz), 29.2, 29.1, 25.4, 24.2 (t, J = 137.5Hz), 22.6, 14.1; ^{31}P NMR (202 MHz, $CDCl_3$) δ 23.38 (d, J = 6.1Hz), 21.74 (d, J = 6.1Hz). HRMS (ESI-qTOF) m/z: $[M+H]^+$ Calcd for $C_{12}H_{29}O_6P_2$ 331.1439; Found 331.1443.

Dimethyl {[isopropoxy(methoxy)phosphoryl]methyl}phosphonate (**2c**) was prepared by following the general procedure described earlier in this paper from ammonium salt **1** (718 mg, 1.72 mmol) and isopropyl tosylate (369 mg, 1.72 mmol). The purification by silica column chromatography with ethyl acetate:methanol (8:2, R_f = 0.4) as the eluent gave the pure product as a colorless oil. Yield 316 mg (71%). ^{1}H NMR (500 MHz, $CDCl_3$) δ 4.84-4.75 (m, 2H), 3.82 (d, J = 11.2 Hz, 3H), 3.81 (d, J = 11.3 Hz, 3H), 3.80 (d, J = 11.3 Hz, 3H), 2.44 (t, J = 21.1Hz, 2H), 1.363 (d, J = 6.2 Hz, 3H), 1.358 (d, J = 6.2 Hz, 3H); ^{13}C NMR (126 MHz, $CDCl_3$) δ 71.9 (d, J = 6.6Hz), 53.2 (d, J = 6.4 Hz), 53.1 (d, J = 6.4 Hz), 52.9 (d, J = 6.3 Hz), 24.8 (t, J = 137.9Hz),

24.1 (d, J = 3.7 Hz), 23.8 (d, J = 5.5 Hz); ^{31}P NMR (202 MHz, $CDCl_3$) δ 23.45 (d, J = 6.3 Hz), 20.67 (d, J = 6.3 Hz). HRMS (ESI-qTOF) m/z: $[M+H]^+$ Calcd for $C_7H_{19}O_6P_2$ 261.0657; Found 261.0659.

Dimethyl {[(but-3-en-1-yloxy)(methoxy)phosphoryl]methyl}phosphonate (**2d**) was prepared by following the general procedure described earlier in this paper from ammonium salt **1** (500 mg, 1.20 mmol) and butenyl tosylate (254 mg, 1.20 mmol). The purification by silica column chromatography with ethyl acetate:methanol (8:2, R_f = 0.4) as the eluent gave the pure product as a colorless oil. Yield 246 mg (75%). ^{1}H NMR (500 MHz, $CDCl_3$) δ 5.85-5.76 (m, 1H), 5.18-5.09 (m, 2H), 4.21-4.14 (m, 2H), 3.82 (d, J = 11.3Hz, 3H), 3.82 (d, J = 11.3Hz, 3H), 3.81 (d, J = 11.3Hz, 3H), 2.47 (t, 21.1Hz, 2H), 2.49-2.43 (m, 2H); ^{13}C NMR (126 MHz, $CDCl_3$) δ 133.4, 117.8, 65.8 (d, J = 6.5 Hz), 53.2 (d, J = 6.2 Hz), 53.2 (d, J = 5.9 Hz), 53.1 (d, J = 6.2 Hz), 34.8 (d, J = 6.2Hz), 53.2 (t, J = 137.6Hz); ^{31}P NMR (202 MHz, $CDCl_3$) δ 23.30 (d, J = 5.9 Hz), 21.94 (d, J = 5.9 Hz). HRMS (ESI-qTOF) m/z: $[M+H]^+$ Calcd for $C_8H_{19}O_6P_2$ 273.0657; Found 273.0659.

Dimethyl {[(but-3-yn-1-yloxy)(methoxy)phosphoryl]methyl}phosphonate (**2e**) was prepared by following the general procedure described earlier in this paper from ammonium salt **1** (500 mg, 1.20 mmol) and butynyl tosylate (252 mg, 1. 20 mmol). The purification by silica column chromatography with ethyl acetate:methanol (8:2, R_f = 0.4) as the eluent gave the pure product as a colorless oil. Yield 197 mg (61%). ^{1}H NMR (500 MHz, $CDCl_3$) δ 4.28-4.17 (m, 2H), 3.83 (d, *J* = 11.4 Hz, 3H), 3.83 (d, *J* = 11.3 Hz, 3H), 3.82 (d, *J* = 11.3 Hz, 3H), 2.61 (td, *J* = 6.7 Hz, 2.6 Hz, 2H), 2.49 (t, *J* = 21.2 Hz, 2H), 2.03 (t, *J* = 2.6 Hz, 1H); ^{13}C NMR (126 MHz, $CDCl_3$) δ 79.6, 70.4, 64.4 (d, J = 6.2 Hz), 53.2 (d, J = 5.9 Hz), 53.2 (d, J = 5.9 Hz), 53.1 (d, J = 6.5 Hz), 24.3 (t,

J = 137.7 Hz), 20.9 (d, J = 6.6 Hz); ^{31}P NMR (202 MHz, $CDCl_3$) δ 21.76 (d, J = 5.4 Hz), 20.93 (d, J = 5.4 Hz). HRMS (ESI-qTOF) m/z: $[M+H]^+$ Calcd for $C_8H_{17}O_6P_2$ 271.0500; Found 271.0502.

Dimethyl {[(isopentyloxy)(methoxy)phosphoryl]methyl}phosphonate (**2f**) was prepared by following the general procedure described earlier in this paper from ammonium salt **1** (600 mg, 1.44 mmol) and isopentyl tosylate (383 mg, 1.58 mmol). The purification by silica column chromatography with ethyl acetate:methanol (8:1, R_f = 0.5) as the eluent gave the pure product as a colorless oil. Yield 247 mg (60%). ^{1}H NMR (500 MHz, $CDCl_3$) δ 4.19-4.11 (m, 2H), 3.86-3.76 (m, 9H), 2.45 (t, J = 21.1Hz, 2H), 1.80-1.70 (m, 1H), 1.63-1.54 (m, 2H), 0.93 (d, J = 6.6Hz); ^{13}C NMR (126 MHz, $CDCl_3$) δ 65.3 (d, J = 6.6Hz), 53.2 (d, J = 7.0Hz), 53.1 (d, J = 6.7Hz), 53.1 (d, J = 6.3Hz), 39.1 (d, J = 6.2Hz), 24.6, 24.2 (t, J = 138.0Hz), 22.4, 22.4; ^{31}P NMR (202 MHz, $CDCl_3$) δ 23.37 (d, J = 5.8Hz), 21.77 (d, J = 5.8Hz). HRMS (ESI-qTOF) m/z: $[M+H]^+$ Calcd for $C_9H_{23}O_6P_2$ 289.0970; Found 289.0977.

Dimethyl {[(3-methylbut-3-en-1-yl)oxy(methoxy)phosphoryl]methyl}phosphonate (**2g**) was prepared by following the general procedure described earlier in this paper from ammonium salt **1** (750 mg, 1.80 mmol) and isopentenyl tosylate (407 mg, 1.80 mmol). The purification by silica column chromatography with ethyl acetate:methanol (8:2, R_f = 0.5) as the eluent gave the pure product as a colorless oil. Yield 375 mg (73%). ^{1}H NMR (600 MHz, $CDCl_3$) δ 4.84 (s, 1H), 4.78 (s, 1H), 4.26-4.21 (m, 2H), 3.82 (d, J = 11.3Hz, 3H), 3.82 (d, J = 11.3Hz, 3H), 3.81 (d, J = 11.4Hz, 3H), 2.46 (t, J = 21.1Hz, 2H), 2.44-2.40 (m, 2H), 1.77 (s, 3H); ^{13}C NMR (151 MHz, $CDCl_3$) δ 141.0, 112.7, 64.8 (d, J = 6.5Hz), 53.2 (d, J = 6.5Hz), 53.1 (d, J = 6.9Hz), 53.1 (d, J = 6.8Hz), 38.5 (d, J = 6.3Hz), 24.2 (t, J = 137.6Hz), 22.4; ^{31}P NMR

(243 MHz, $CDCl_3$) δ 22.06 (d, J = 5.8Hz), 20.65 (d, J = 5.8Hz). HRMS (ESI-qTOF) m/z: $[M+H]^+$ Calcd for $C_9H_{21}O_6P_2$ 287.0813; Found 287.0816.

{[Butoxy(hydroxy)phosphoryl]methyl}phosphonic acid (**3a**) was prepared by following the general procedure described earlier in this paper. Butyl trimethyl methylenebisphosphonate (**2a**, 170 mg, 0.62 mmol) and trimethylsilylbromide (378 mg, 2.47 mmol) were stirred in dry acetonitrile for 4 hours. Purification of the crude product (139 mg) by HPCCC resulted in 92 mg (64%) of the pure product as a white solid, mp 128-130°C. ^{1}H NMR (500 MHz, D_2O) δ 3.88 (q, J = 6.9 Hz, 2H), 2.06 (t, *J* = 19.6 Hz, 2H), 1.67-1.54 (m, 2H), 1.44-1.31 (m, 2H), 0.92 (t, *J* = 7.4 Hz, 3H); ^{13}C NMR (126 MHz, D_2O) δ 67.5 (d, J = 5.7 Hz), 35.2 (d, J = 5.9 Hz), 30.9 (dd, J = 125.4 Hz, 120.6 Hz), 21.2, 16.0; ^{31}P NMR (202 MHz, D_2O) δ 22.32 (d, J = 8.6 Hz), 11.87 (d, J = 8.6 Hz). HRMS (ESI-TOF) m/z: $[M-H]^-$ Calcd for $C_5H_{13}O_6P_2$ 231.0187; Found 231.0206.

{[Octyloxy(hydroxy)phosphoryl]methyl}phosphonic acid (**3b**) was prepared by following the general procedure described earlier in this paper. Octyl trimethyl methylenebisphosphonate (**2b**, 244 mg, 0.74 mmol) and trimethylsilylbromide (451 mg, 2.95 mmol) were stirred in dry acetonitrile for 4 hours. Purification of the crude product (205 mg) by HPCCC resulted in 111 mg (52%) of the pure product as a white solid, mp 133-135°C. ^{1}H NMR (500 MHz, D_2O) δ 3.85 (q, J = 6.9 Hz, 2H), 2.00 (t, *J* = 19.5 Hz, 2H), 1.68-1.57 (m, 2H), 1.41-1.22 (m, 10H), 0.93-0.81 (m, 3H); ^{13}C NMR (126 MHz, D_2O) δ 67.6 (d, J = 5.6Hz), 34.0, 33.1 (d, J = 6.0 Hz), 31.4, 31.4 (dd, J = 125.0Hz, 119.2Hz), 31.3, 27.9, 24.9, 16.2; ^{31}P NMR (202 MHz, D_2O) δ 22.37 (d, J = 8.5 Hz), 12.15 (d, J = 8.5 Hz). HRMS (ESI-TOF) m/z: $[M-H]^-$ Calcd for $C_9H_{21}O_6P_2$ 287.0813; Found 287.0826.

{[Isopropoxy(hydroxy)phosphoryl]methyl}phosphonic acid (**3c**) was prepared by following the general procedure described earlier in this paper. Isopropyl trimethyl methylenebisphosphonate (**2c**, 302 mg, 1.16 mmol) and trimethylsilylbromide (889 mg, 5.80 mmol) were stirred in dry acetonitrile for 2 hours, resulting in 254 mg of crude product. Purification of 80 mg of the crude product by HPCCC led to 39 mg (49%) of the pure product as a white solid, mp 128-130°C. ^{1}H NMR (600 MHz, D_2O) δ 4.51-4.42 (m, 1H), 2.00 (t, J = 19.8Hz, 2H), 1.26 (d, J = 6.2Hz, 6H); ^{13}C NMR (151 MHz, D_2O) δ 71.1 (d, J = 5.7Hz), 32.5 (dd, J = 127.5Hz, 118.8Hz), 26.5 (d, J = 3.9Hz); ^{31}P NMR (243 MHz, D_2O) δ 20.99 (d, J = 8.1Hz), 12.00 (d, J = 8.1Hz). HRMS (ESI-TOF) m/z: $[M-H]^-$ Calcd for $C_4H_{11}O_6P_2$ 217.0031; Found 217.0061.

{[(But-3-en-1-yloxy)(hydroxy)phosphoryl]methyl}phosphonic acid (**3d**). Butenyl trimethyl methylenebisphosphonate (**2d**, 237 mg, 0.87 mmol) and trimethylsilylbromide (667 mg, 4.35 mmol) were stirred in dry acetonitrile for 2 hours at −5 °C. The reaction mixture was transferred to a beaker with 10 mL of ice cold water and 2 equiv of sodium hydroxide (2 M) were added. The solvents were evaporated by freeze drying and the crude product (322 mg) was obtained. Purification of 160 mg of the crude product by HPCCC resulted in 35 mg (29%) of the pure product as an amorphous solid. ^{1}H NMR (500 MHz, D_2O) δ 5.96-5.87 (m, 1H), 5.23-5.08 (m, 2H), 3.93 (q, J = 6.8Hz, 2H), 2.46-2.34 (m, 2H), 2.01 (t, J = 19.6Hz, 2H); ^{13}C NMR (126 MHz, D_2O) δ 138.4, 119.6, 66.5 (d, J = 5.5Hz), 37.5 (d, J = 6.1Hz), 31.3 (dd, J =126.0Hz, 119.1Hz); ^{31}P NMR (202 MHz, D_2O) δ 22.30 (d, J = 8.4Hz), 11.80 (d, J = 8.4Hz). HRMS (ESI-TOF) m/z: $[M-H]^-$ Calcd for $C_5H_{11}O_6P_2$ 229.0031; Found 229.0046.

{[(But-3-yn-1-yloxy)(hydroxy)phosphoryl]methyl}phosphonic acid (**3e**) Butynyl trimethyl methylenebisphosphonate (**2e**, 190 mg, 0.70 mmol) and trimethylsilylbromide (538 mg, 3.52 mmol) were stirred in dry acetonitrile for 2 hours at −5 °C. The reaction mixture was transferred to a beaker with 10 mL of ice cold water and 2 equiv of sodium hydroxide (2 M) were added. The pure product was crystallized in its disodium form as a white solid (130 mg, 68%) from water with acetone, mp >250°C. ^{1}H NMR (600 MHz, D_2O) δ 4.06-3.98 (m, 2H), 2.56 (td, J = 6.6Hz, 2.6Hz, 2H), 2.39 (t, J = 2.6Hz, 1H), 2.32 (t, 20.2Hz, 2H); ^{13}C NMR (151 MHz, D_2O) δ 85.0, 73.5, 65.8 (d, J = 4.9Hz), 29.8 (t, J = 125.0Hz), 23.3 (d, J = 6.2Hz); ^{31}P NMR (243 MHz, D_2O) δ 22.57 (d, J = 9.1Hz), 11.66 (d, J = 9.1Hz). HRMS (ESI-TOF) m/z: $[M-H]^-$ Calcd for $C_5H_9O_6P_2$ 226.9874; Found 226.9876.

{[(Isopentyloxy)(hydroxy)phosphoryl]methyl}phosphonic acid (**3f**) was prepared by following the general procedure described earlier in this paper. Isopentyl trimethyl methylenebisphosphonate (**2f**, 243 mg, 0.84 mmol) and trimethylsilylbromide (645 mg, 4.22 mmol) were stirred in dry acetonitrile for 2 hours, resulting in 206 mg of crude product. Purification of 137 mg of the crude product by HPCCC resulted in 80 mg (58%) of the pure product as a white solid, mp 95-97°C. ^{1}H NMR (500 MHz, D_2O) δ 3.96-3.82 (m, 2H), 1.99 (t, J = 19.6 Hz, 2H), 1.75-1.65 (m, 1H), 1.58-1.46 (m, 2H), 0.91 (d, J = 6.7Hz, 6H); ^{13}C NMR (126 MHz, D_2O) δ 66.0 (d, J = 5.6Hz), 42.0 (d, J = 5.8Hz), 31.5 (dd, J = 126.1Hz, 118.8Hz), 27.1, 24.7; ^{31}P NMR (202 MHz, D_2O) δ 22.31 (d, J = 8.5Hz), 11.91 (d, J = 8.5Hz). HRMS (ESI-TOF) m/z: $[M-H]^-$ Calcd for $C_6H_{15}O_6P_2$ 245.0344; Found 245.0388.

{[(3-Methylbut-3-en-1-yl)oxy (hydroxy)phosphoryl]methyl}phosphonic acid (**3g**). Isopentenyl trimethyl methylenebisphosphonate (**2g**, 180 mg, 0.63 mmol) and

trimethylsilylbromide (337 mg, 2.20 mmol) were mixed in dry acetonitrile for 4 hours at −5 °C. The reaction mixture was evaporated, 10 mL of methanol were added and evaporated after 30 minutes. Then, 10 mL of methanol were added, the mixture was cooled to 0 °C, 1 equiv of NaOH (10 M) was added, the solvents were evaporated and the crude product (167 mg) was obtained. Purification of 64 mg of the crude product by HPCCC led to 20 mg (31%) of the pure product as a white solid, mp >250°C. The compound started foaming at 189°C, but did not melt. ^{1}H NMR (500 MHz, D_2O) δ 4.86 (d, J = 14.2Hz, 2H), 4.00 (q, J = 6.8Hz, 2H), 2.39 (t, J = 6.5Hz, 2H), 2.00 (t, J = 19.5Hz, 2H), 1.78 (s, 3H); ^{13}C NMR (126 MHz, D_2O) δ 146.9, 114.3, 65.4 (d, J = 5.4Hz), 41.2 (d, J = 6.2Hz), 31.5 (dd, J = 126.0Hz, 118.3Hz), 24.6; ^{31}P NMR (202 MHz, D_2O) δ 22.40 (d, J = 8.7Hz), 11.93 (d, J = 8.7Hz). HRMS (ESI-TOF) m/z: $[M-H]^-$ Calcd for $C_6H_{13}O_6P_2$ 243.0187; Found 243.0164.

References

1. Weisell, J.; Vepsäläinen, J.; Turhanen, P. A. *Beilstein J. Org. Chem.* **2015***, 11*, 2189-2193.

2. Vepsäläinen, J.; Pohjala, E.; Nupponen, H.; Vainiotalo, P.; Ahlgren, M. *Phosphorus Sulfur Silicon Relat. Elem.* **1992***, 70*, 183-203.

3. Gorican, H.; Grdenic, D. *Proc. Chem. Soc. , London.* **1960**, 288-289.

4. Timofeeva, V. K. *Tsvetn. Met. (Moscow).* **1965***, 38*, 74-75.

5. Belmant, C.; Bonneville, M.; Peyrat, M. A.; Fournie, J.; Kozikowski, A. P. U.S. Patent 6,624,151 B1, Sep 23, 2003.

6. Puljula, E.; Turhanen, P.; Vepsäläinen, J.; Monteil, M.; Lecouvey, M.; Weisell, J. *ACS Med. Chem. Lett.* **2015***, 6*, 397-401.

7. Vepsäläinen, J. J.; Kivikoski, J.; Ahlgrén, M.; Nupponen, H. E.; Pohjala, E. K. *Tetrahedron.* **1995***, 51*, 6805-6818.
