# Supplementary material for: Synthesis of medronic acid monoesters and their purification by high-performance countercurrent chromatography or by hydroxyapatite
Source: Beilstein J Org Chem. 2016 Oct 7;12:2145–9. doi: 10.3762/bjoc.12.204 (PMC5082484; doi:10.3762/bjoc.12.204)

Supporting Information File 2

for

Synthesis of medronic acid monoesters and their purification by high-performance countercurrent chromatography or by hydroxyapatite

Elina Puljula*, Jouko Vepsäläinen and Petri A. Turhanen

Address: School of Pharmacy, University of Eastern Finland, Biocenter Kuopio, P.O.Box 1627, FI-70211 Kuopio, Finland

Email: Elina Puljula - elina.puljula@uef.fi

* Corresponding author

^{1}H, ^{13}C, and ^{31}P NMR spectra and an example of HPCCC chromatogram

EP-071015-2 10 1 "E:\NMR 2015"

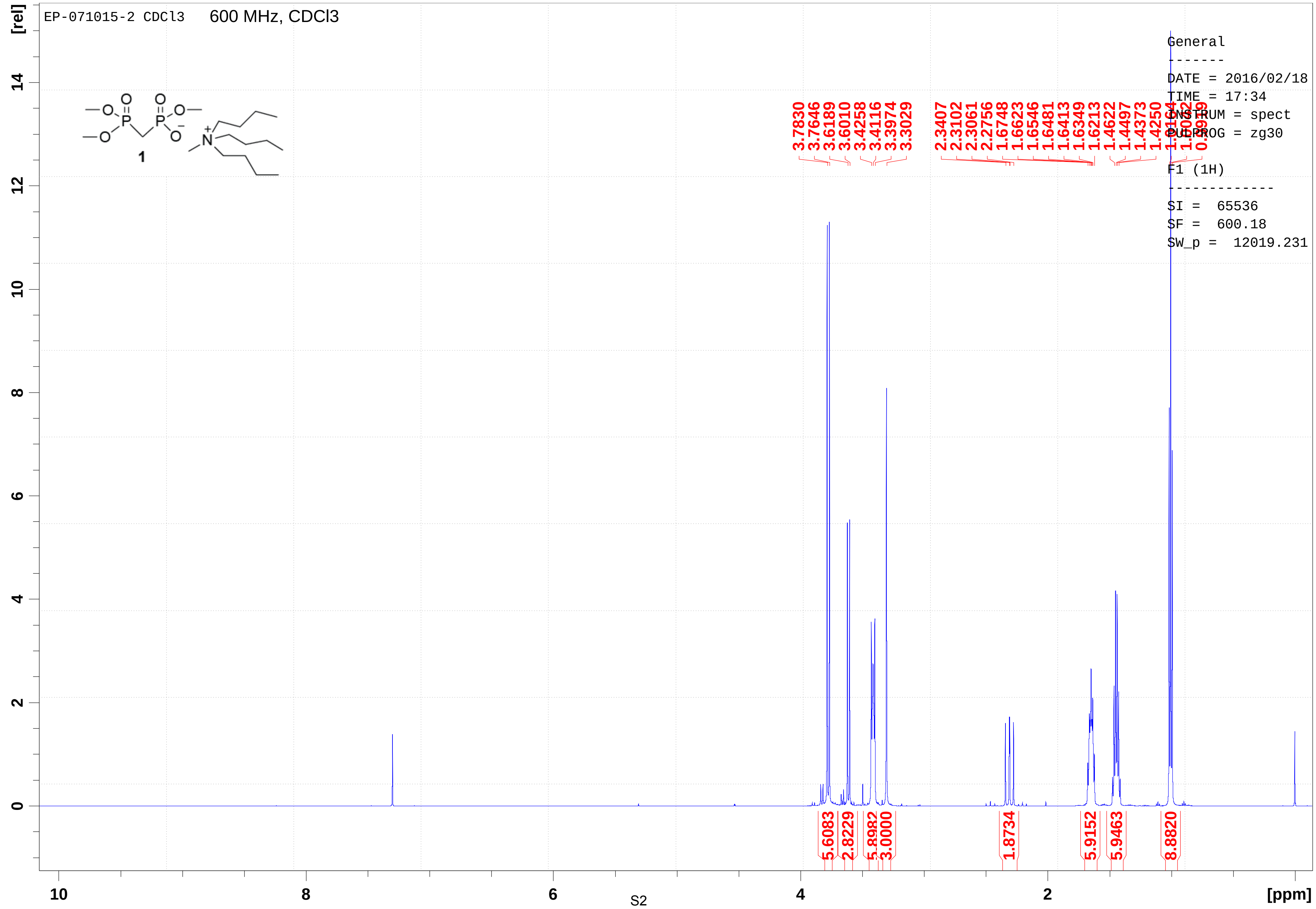

EP-071015-2 12 1 "E:\NMR 2015"

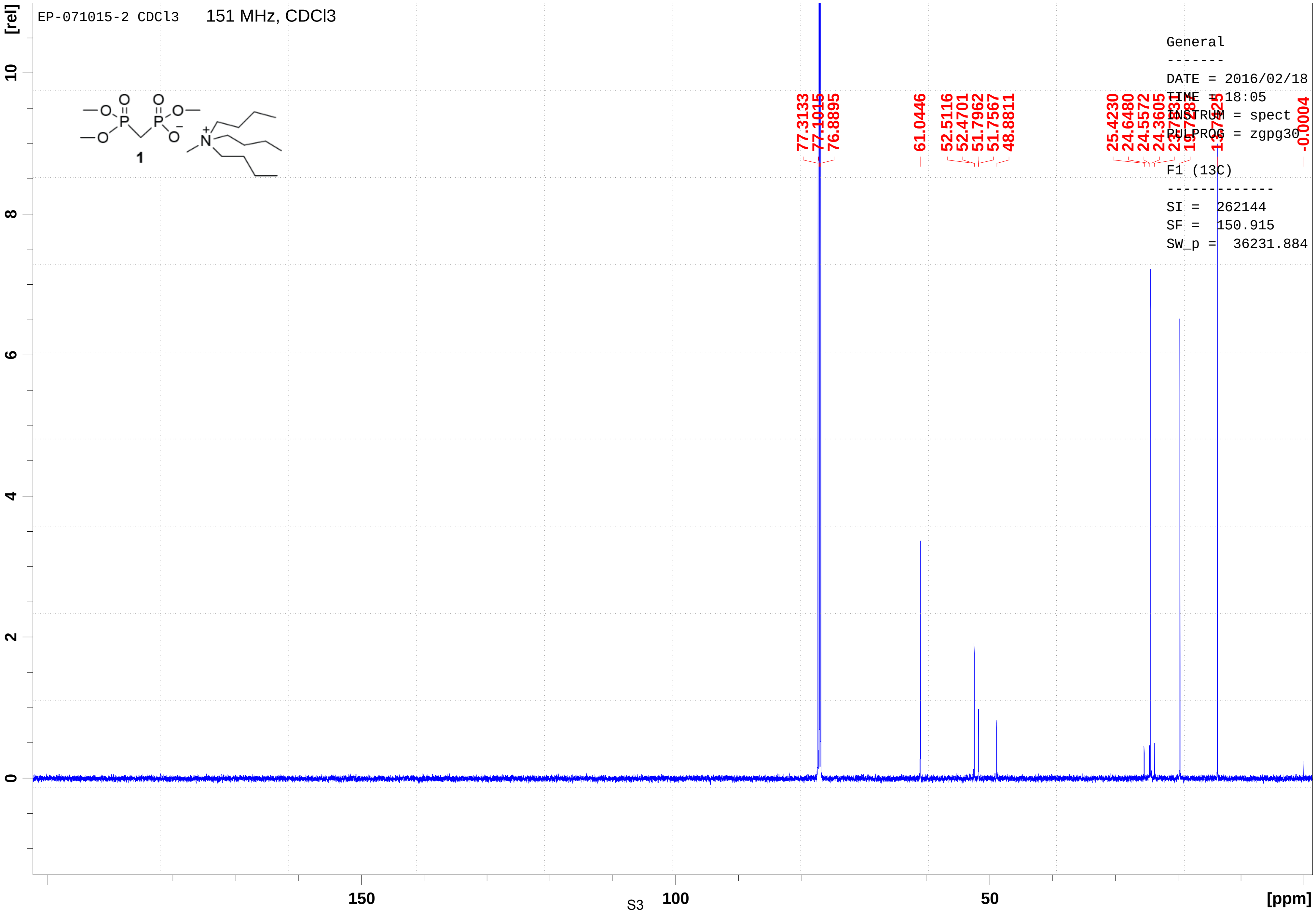

150725-1 12 1 "E:\NMR 2015"

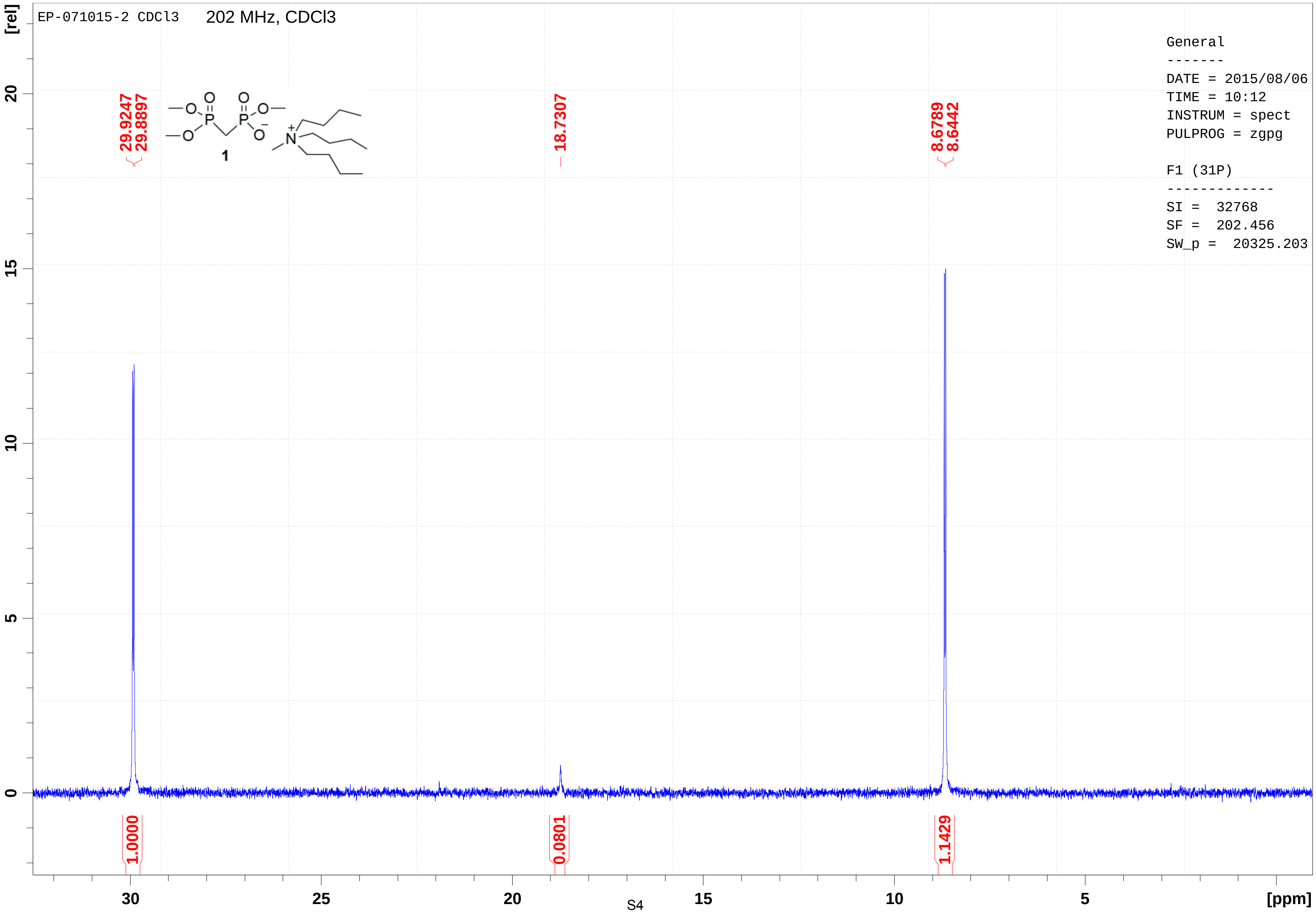

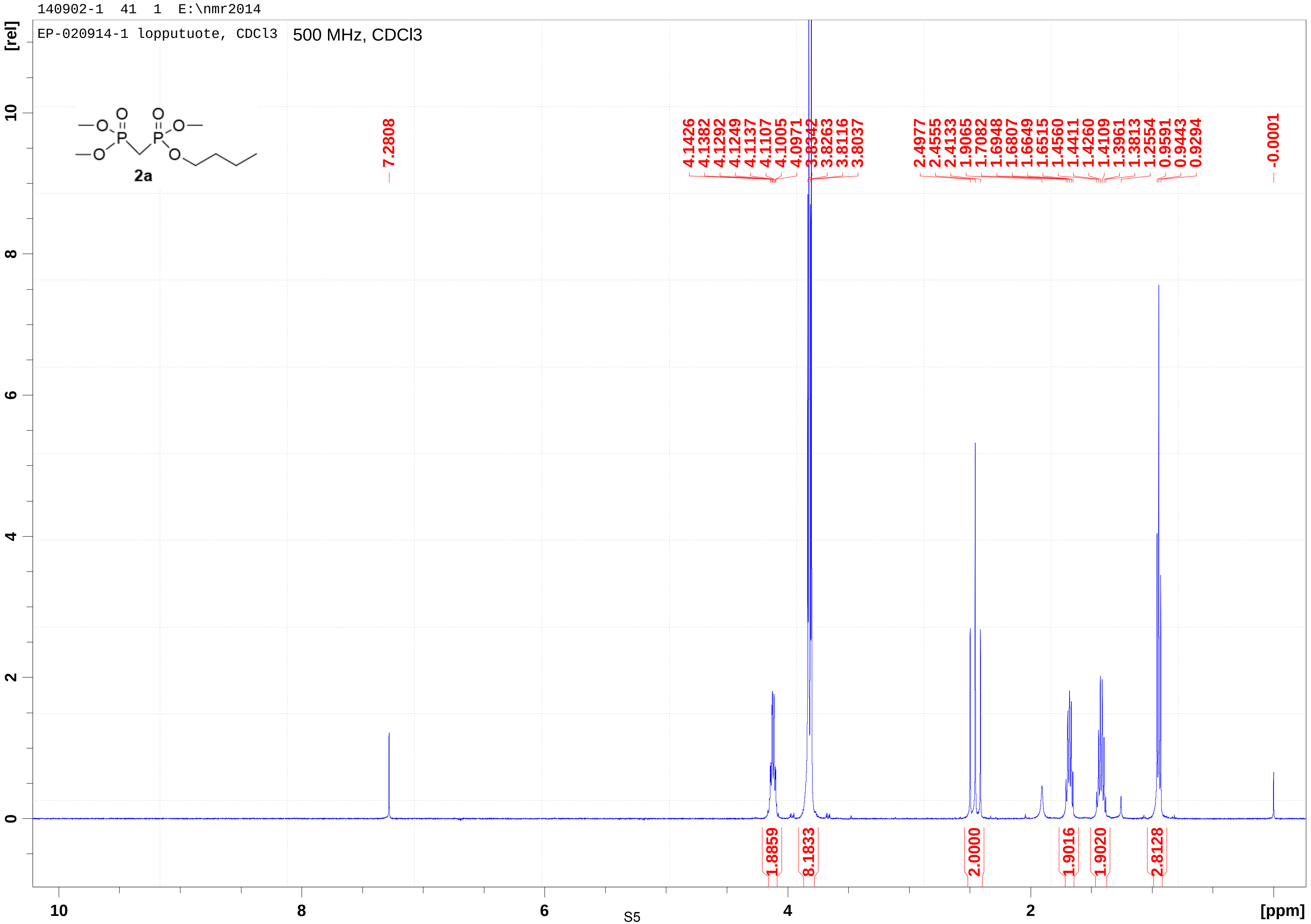
140902-1 41 1 E:\nmr2014
EP-020914-1 lopputuote, CDCl3
500 MHz, CDCl3
2a
7.2808
4.1426
4.1382
4.1292
4.1249
4.1137
4.1107
4.1005
4.0971
3.8342
3.8263
3.8116
3.8037
2.4977
2.4555
2.4133
1.9065
1.7082
1.6948
1.6807
1.6649
1.6515
1.4560
1.4411
1.4260
1.4109
1.3961
1.3813
1.2554
0.9591
0.9443
0.9294
-0.0001
1.8859
8.1833
2.0000
1.9016
1.9020
2.8128
[rel]
[ppm]

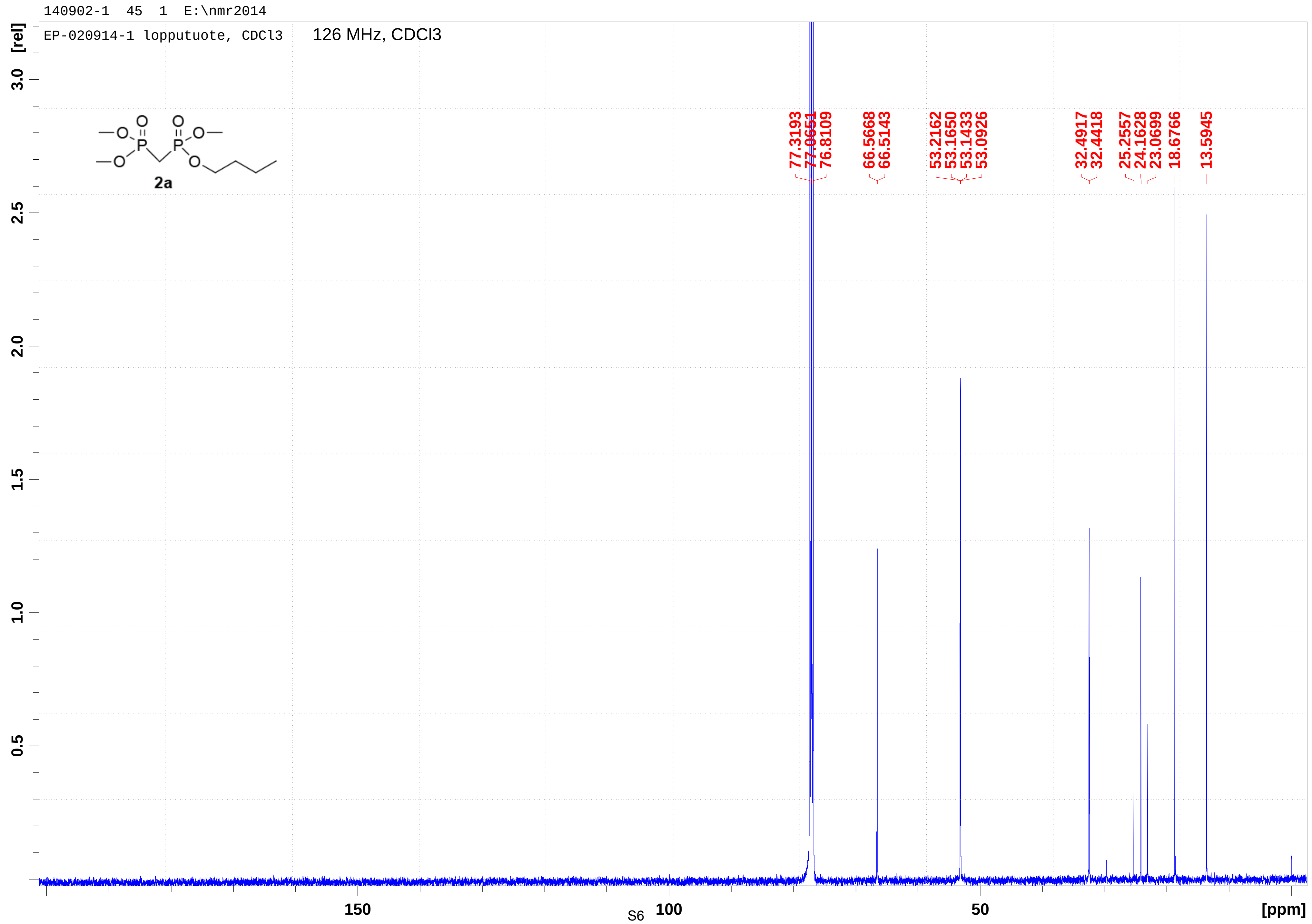

140902-1 45 1 E:\nmr2014
EP-020914-1 lopputuote, CDCl3
126 MHz, CDCl3
2a
77.3193
77.0651
76.8109
66.5668
66.5143
53.2162
53.1650
53.1433
53.0926
32.4917
32.4418
25.2557
24.1628
23.0699
18.6766
13.5945
[rel]
3.0
2.5
2.0
1.5
1.0
0.5
150
100
50
[ppm]

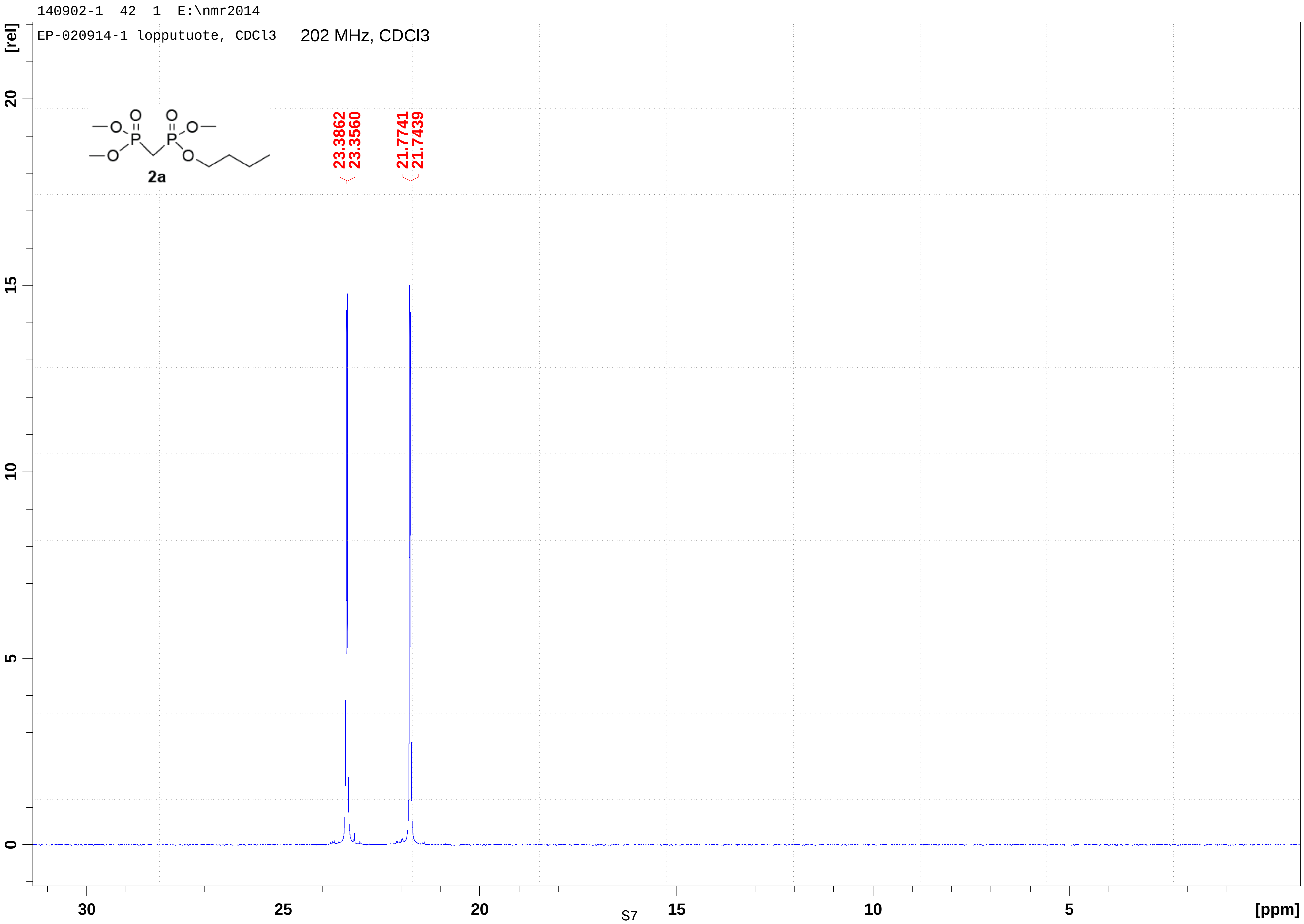
140902-1 42 1 E:\nmr2014
EP-020914-1 lopputuote, CDCl3
202 MHz, CDCl3
2a
23.3862
23.3560
21.7741
21.7439
[rel]
[ppm]

140902-2 41 1 E:\nmr2014

EP-020914-2 lopputuote, CDCl3 500 MHz, CDCl3

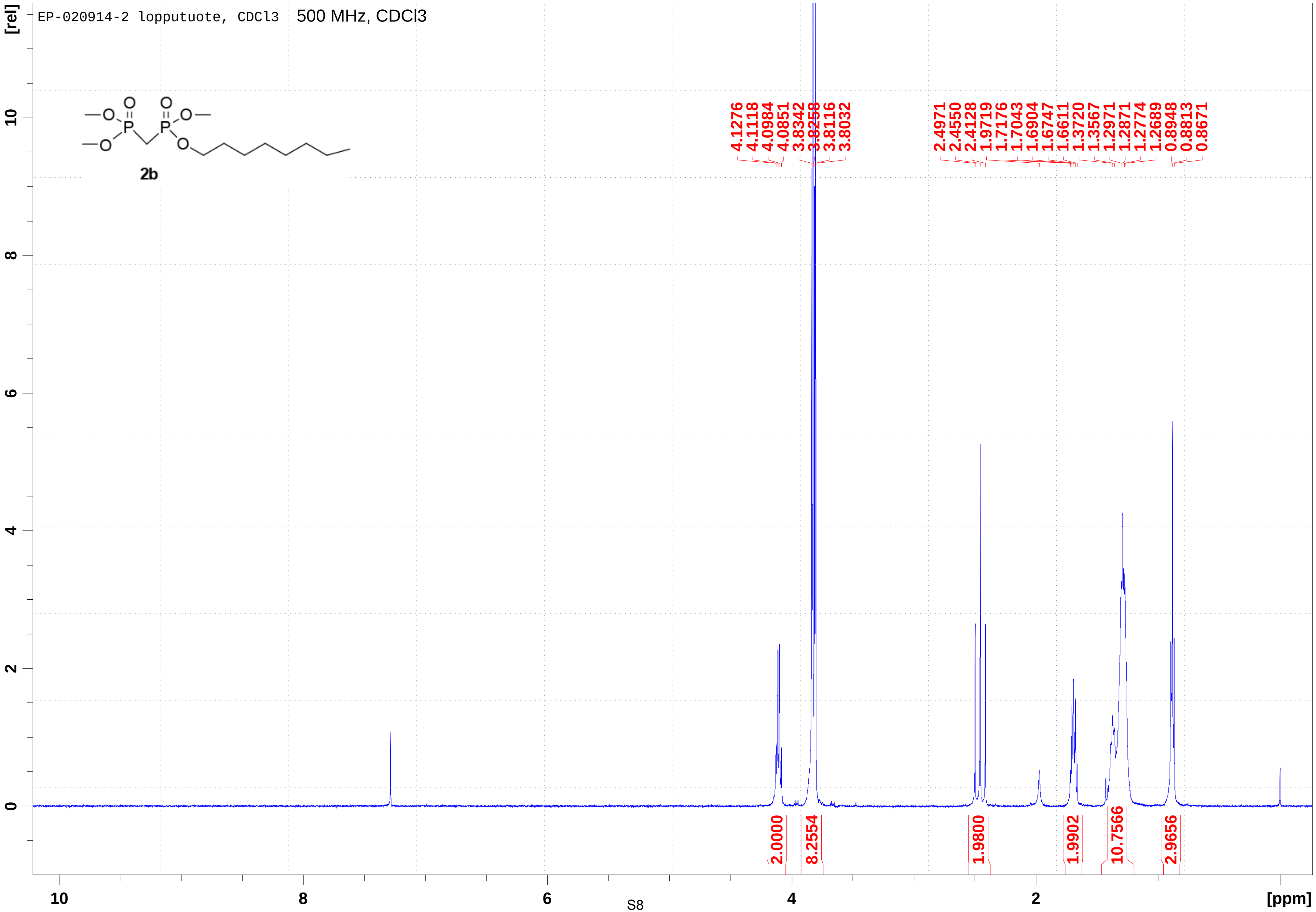

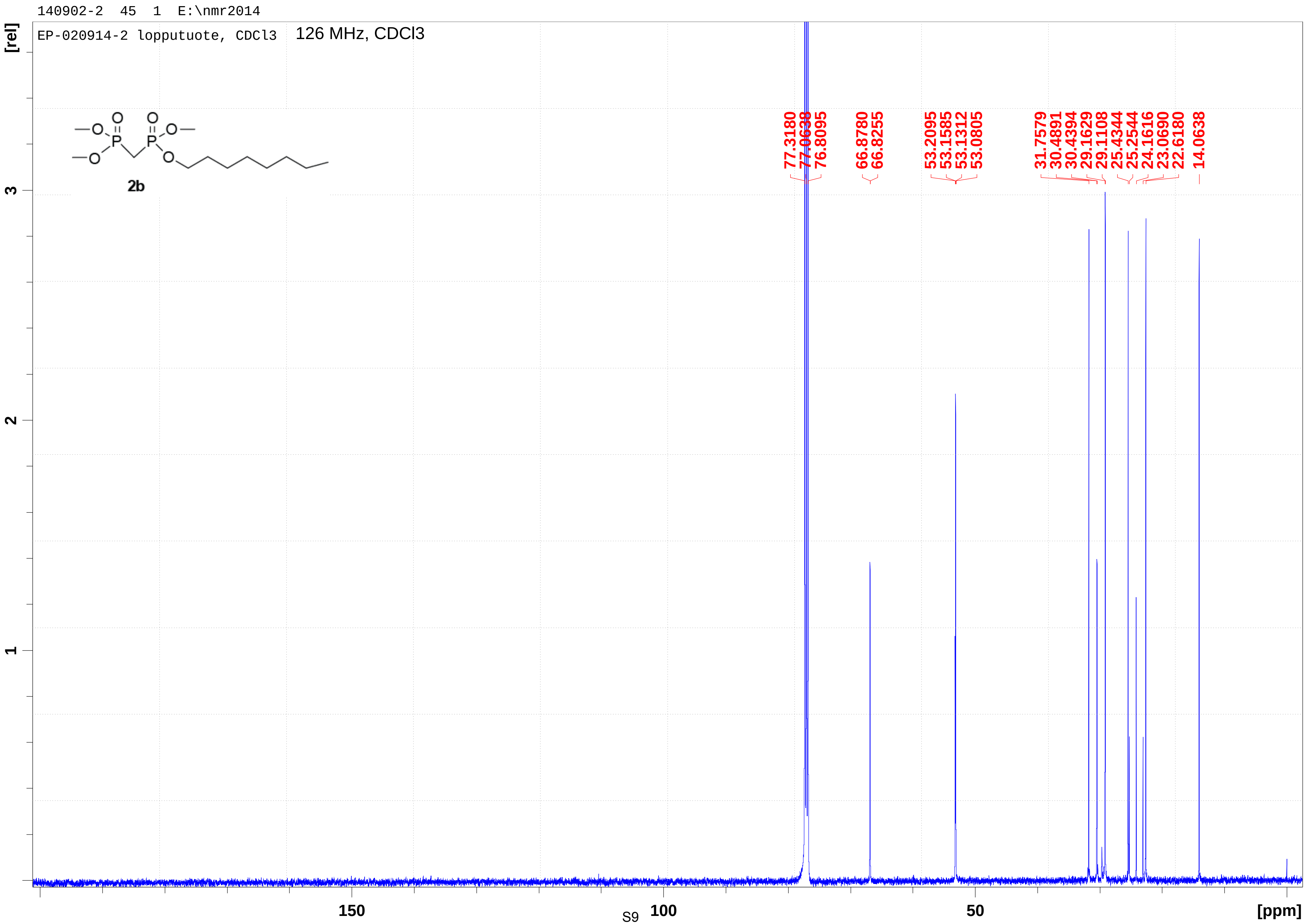
140902-2 45 1 E:\nmr2014
EP-020914-2 lopputuote, CDCl3
126 MHz, CDCl3
2b
77.3180
77.0638
76.8095
66.8780
66.8255
53.2095
53.1585
53.1312
53.0805
31.7579
30.4891
30.4394
29.1629
29.1108
25.4344
25.2544
24.1616
23.0690
22.6180
14.0638
[rel]
3
2
1
150
100
50
[ppm]

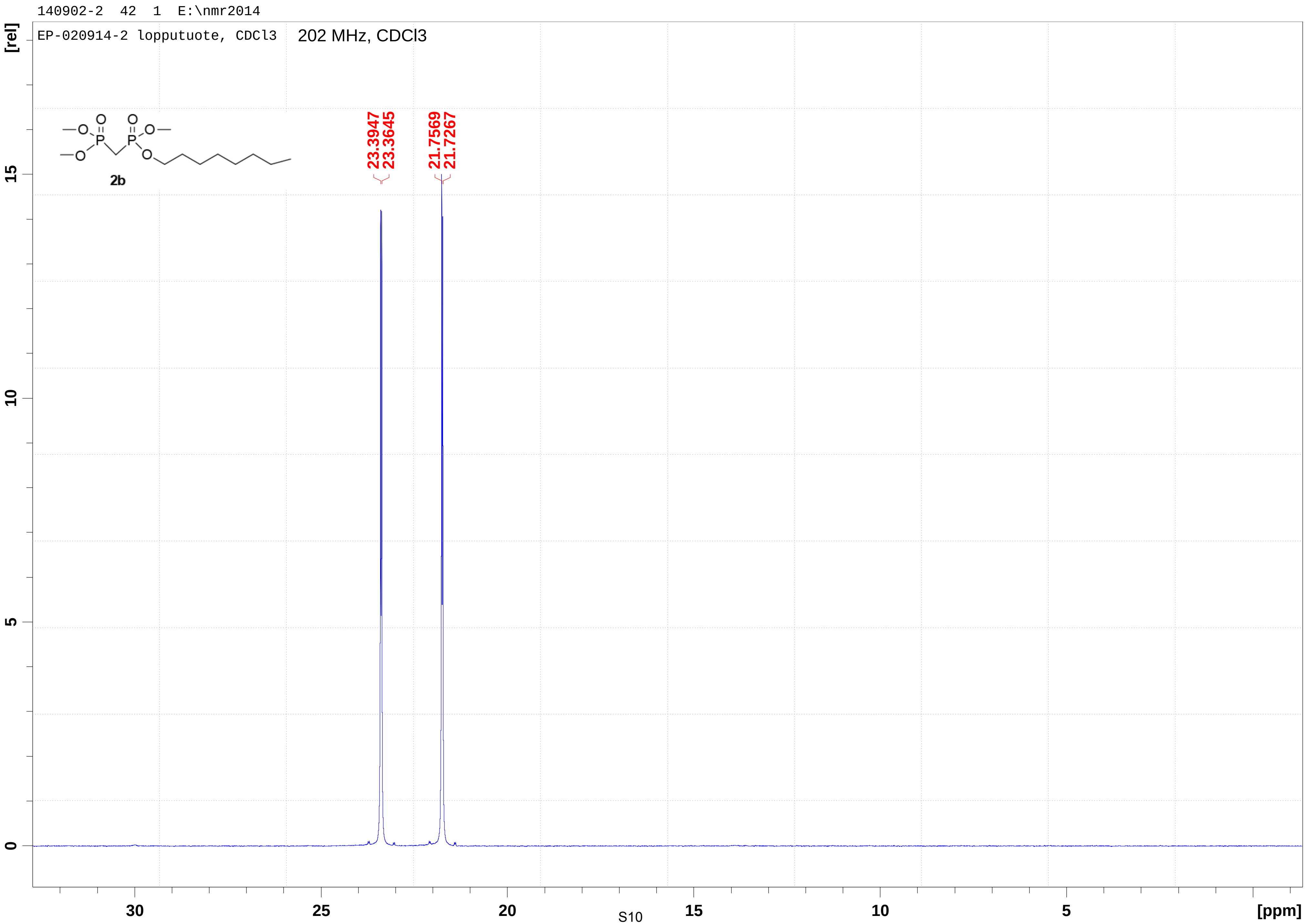
140902-2 42 1 E:\nmr2014
EP-020914-2 lopputuote, CDCl3
202 MHz, CDCl3
2b
23.3947
23.3645
21.7569
21.7267
[rel]
15
10
5
0
30
25
20
15
10
5
[ppm]

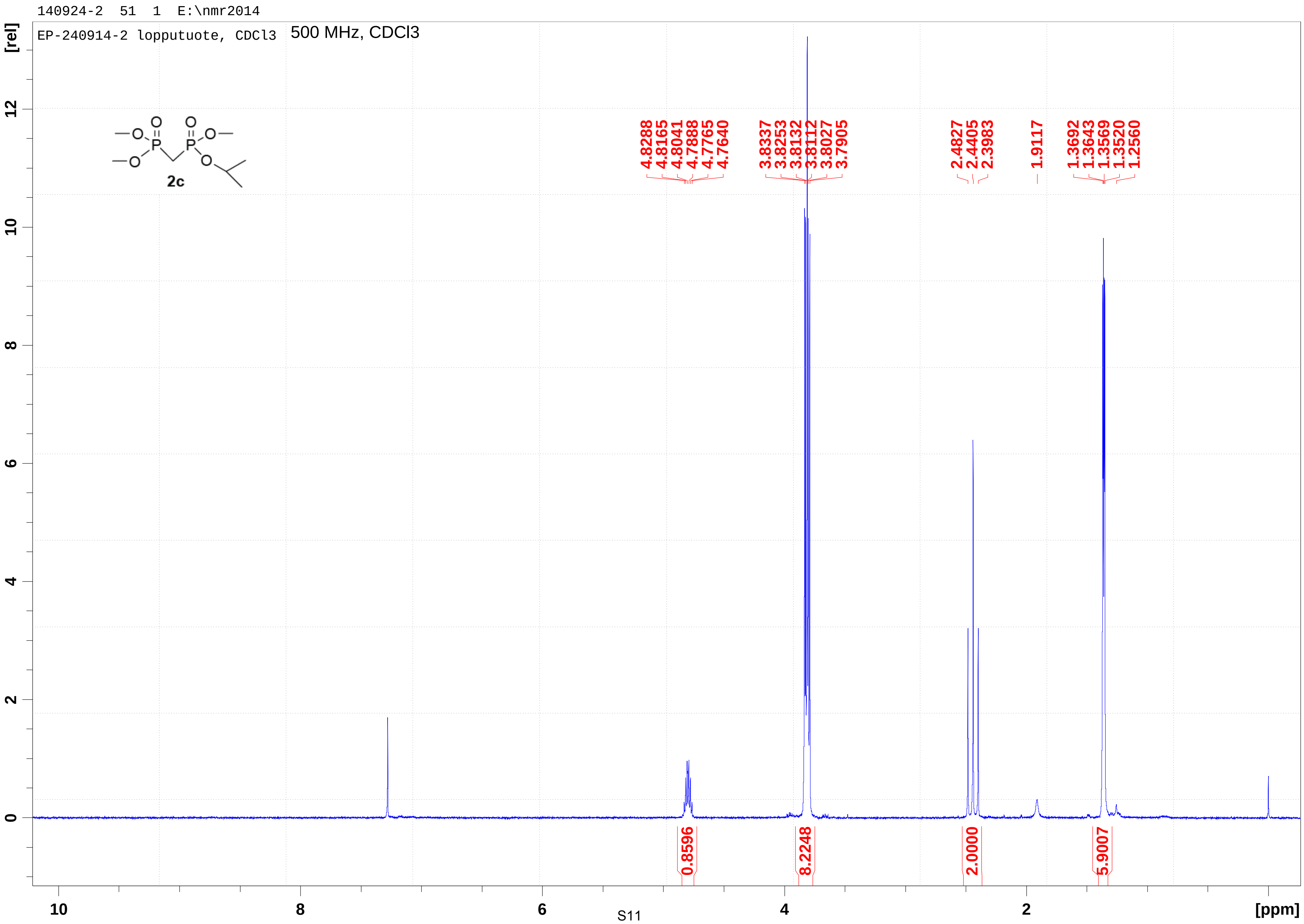
140924-2 51 1 E:\nmr2014
EP-240914-2 lopputuote, CDCl3
500 MHz, CDCl3
2c
4.8288
4.8165
4.8041
4.7888
4.7765
4.7640
3.8337
3.8253
3.8132
3.8112
3.8027
3.7905
2.4827
2.4405
2.3983
1.9117
1.3692
1.3643
1.3569
1.3520
1.2560
0.8596
8.2248
2.0000
5.9007
[rel]
[ppm]

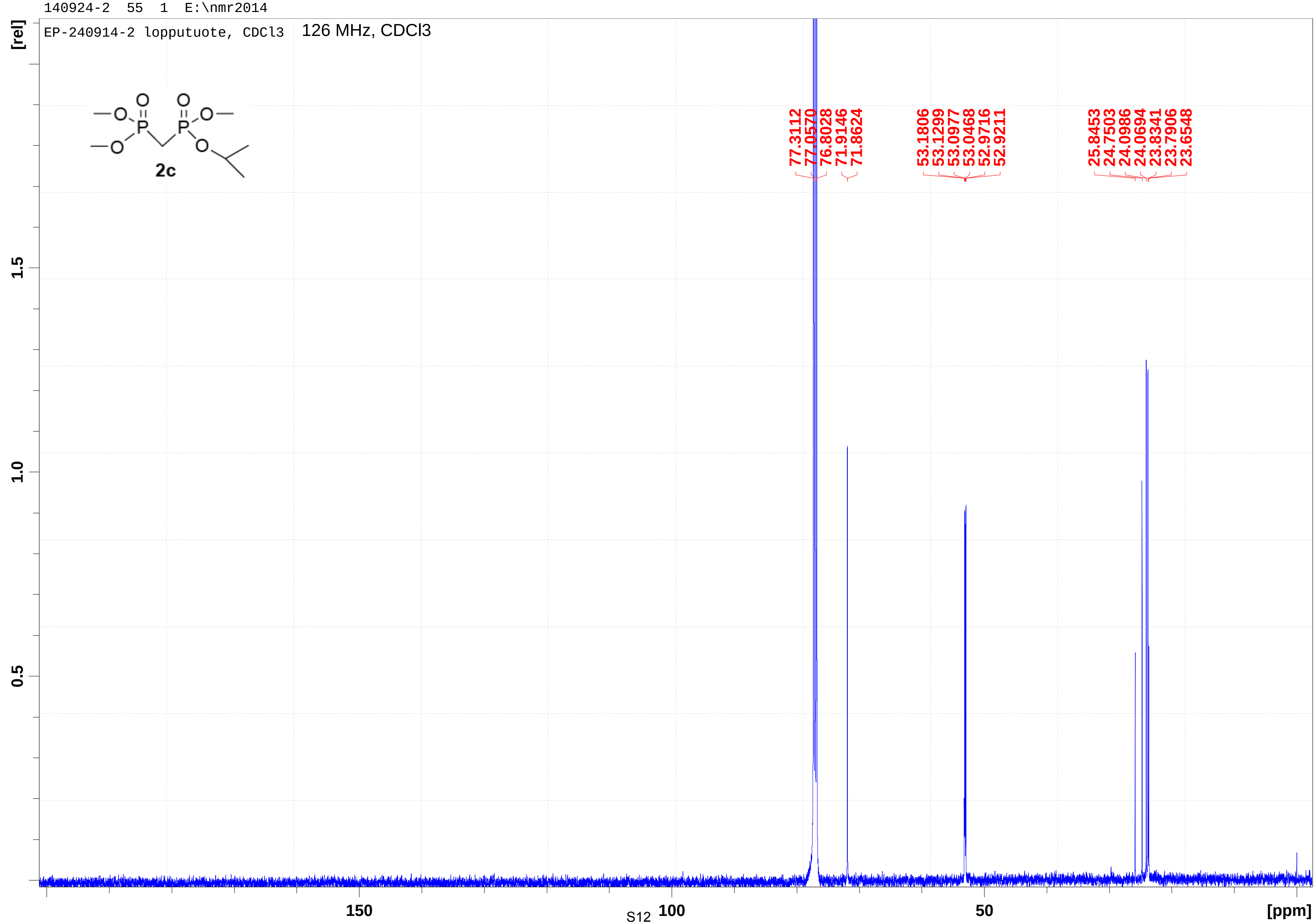
140924-2 55 1 E:\nmr2014
EP-240914-2 lopputuote, CDCl3
126 MHz, CDCl3
2c
77.3112
77.0570
76.8028
71.9146
71.8624
53.1806
53.1299
53.0977
53.0468
52.9716
52.9211
25.8453
24.7503
24.0986
24.0694
23.8341
23.7906
23.6548
[rel]
1.5
1.0
0.5
150
100
50
[ppm]

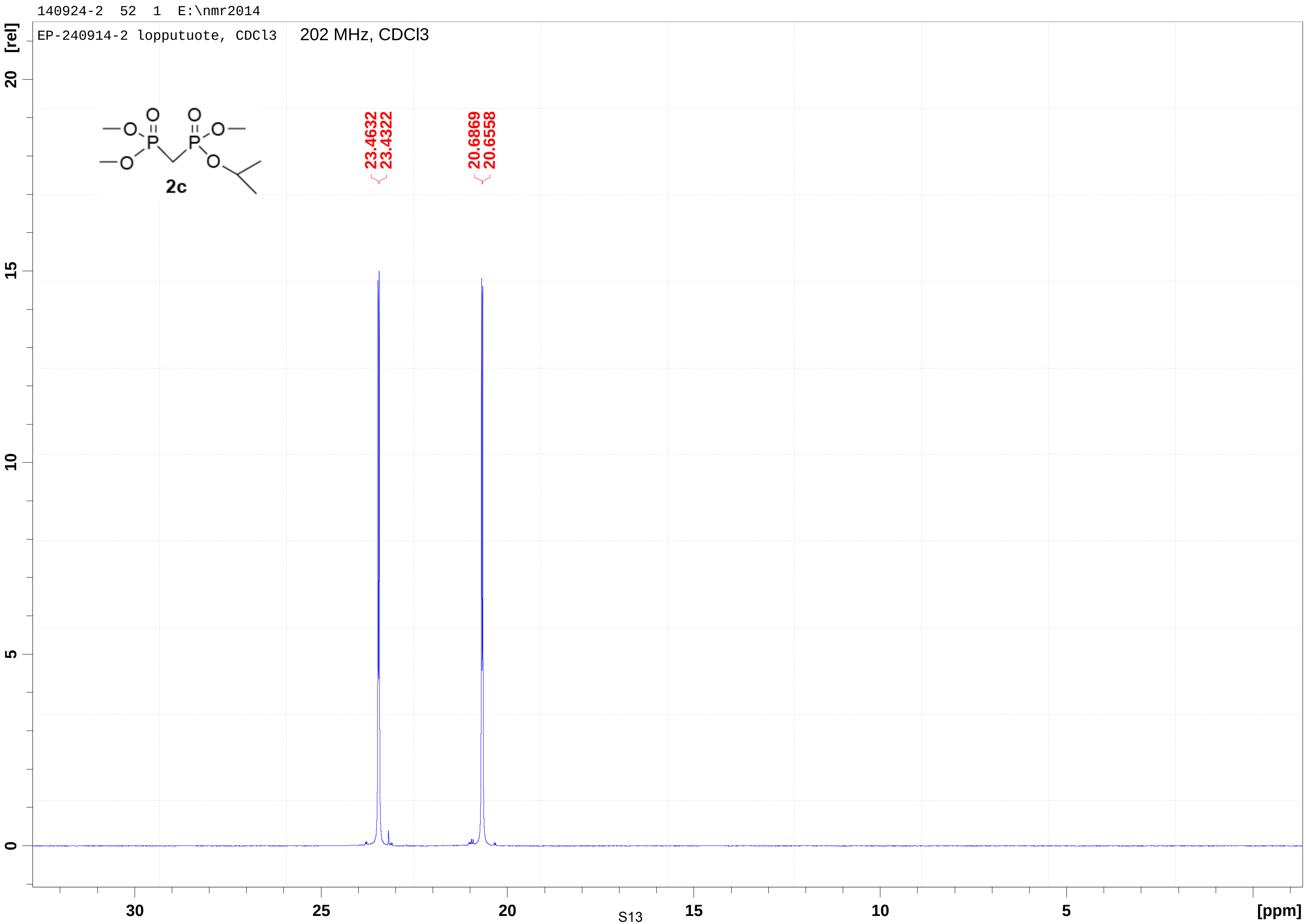
140924-2 52 1 E:\nmr2014
EP-240914-2 lopputuote, CDCl3
202 MHz, CDCl3
2c
23.4632
23.4322
20.6869
20.6558
[rel]
20
15
10
5
0
30
25
20
15
10
5
[ppm]

141027-1 41 1 E:\nmr2014

EP-271014-1 lopputuote, CDCl3

500 MHz, CDCl3

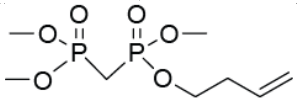

2d

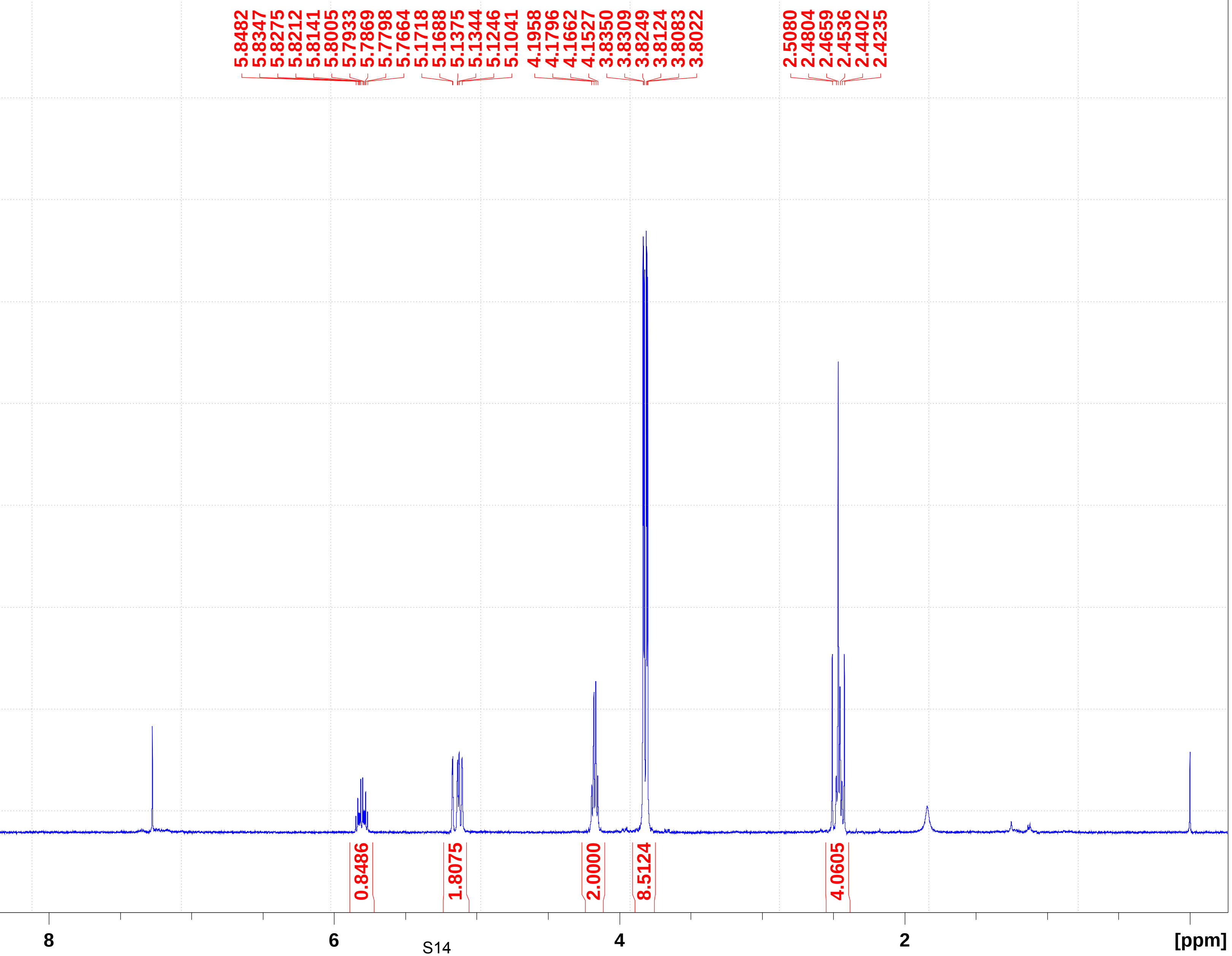

141027-1 45 1 E:\nmr2014

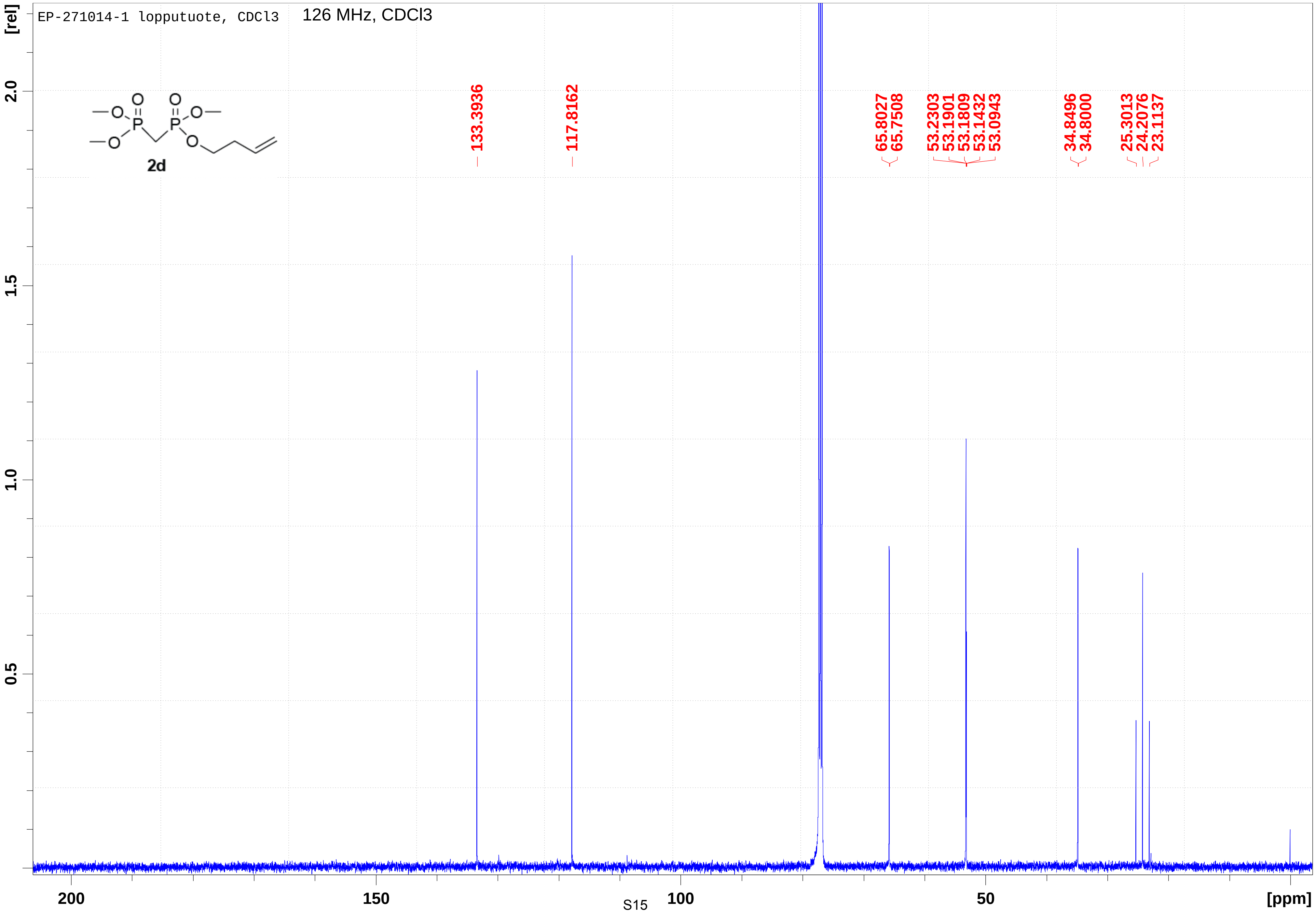

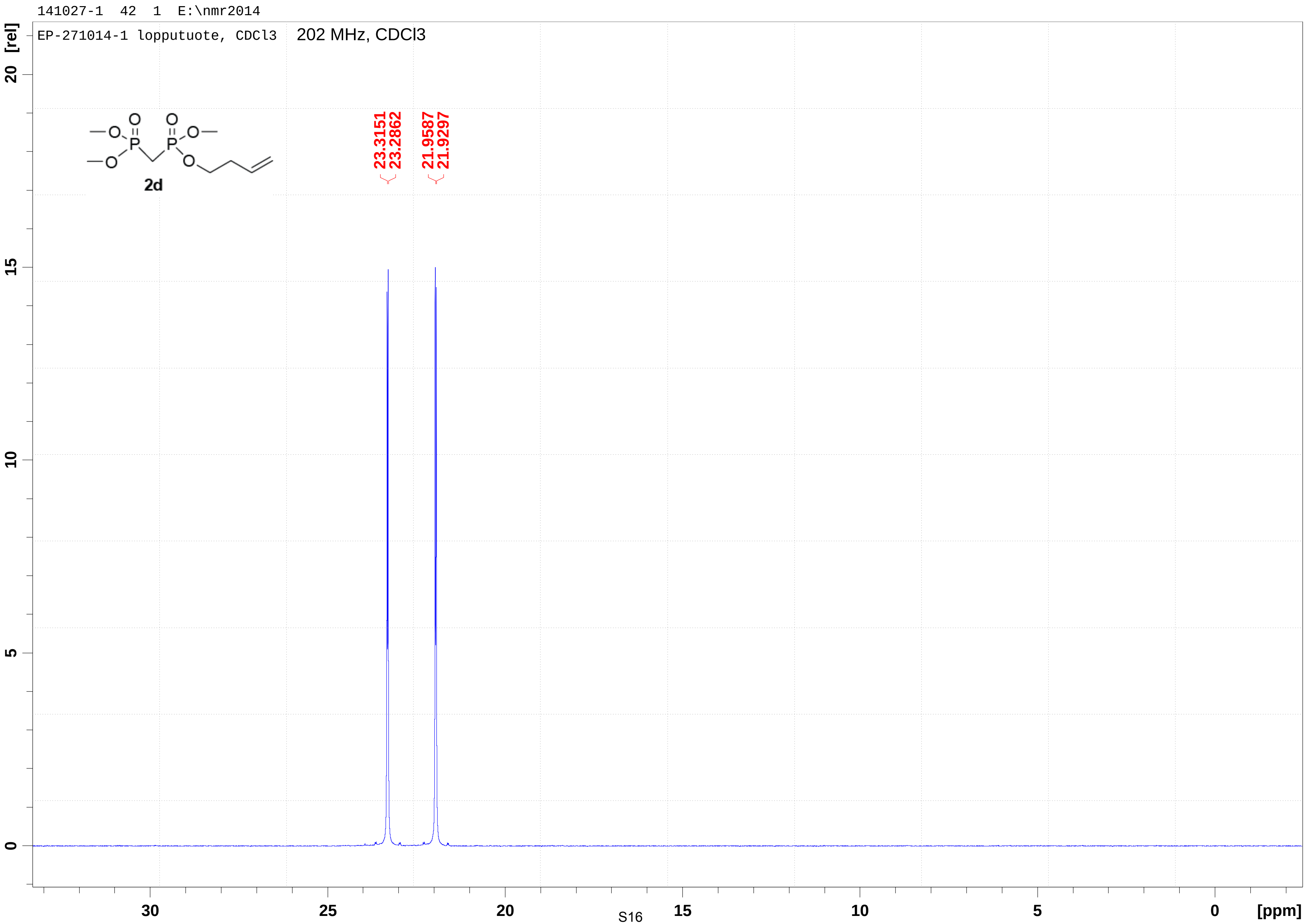

141027-1 42 1 E:\nmr2014
EP-271014-1 lopputuote, CDCl3
202 MHz, CDCl3
2d
23.3151
23.2862
21.9587
21.9297
[rel]
[ppm]

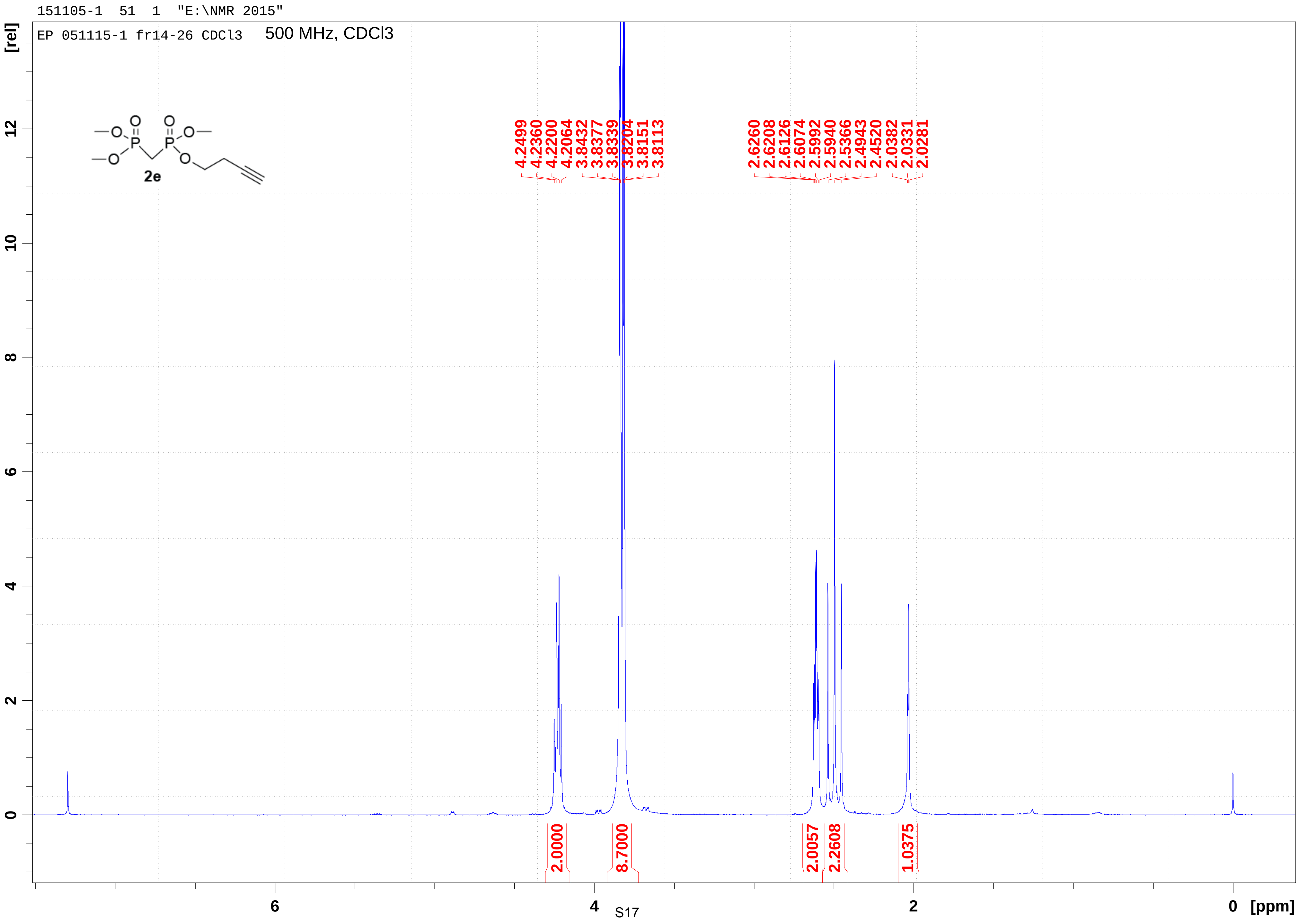
151105-1 51 1 "E:\NMR 2015"
EP 051115-1 fr14-26 CDCl3
500 MHz, CDCl3
2e
4.2499
4.2360
4.2200
4.2064
3.8432
3.8377
3.8339
3.8204
3.8151
3.8113
2.6260
2.6208
2.6126
2.6074
2.5992
2.5940
2.5366
2.4943
2.4520
2.0382
2.0331
2.0281
2.0000
8.7000
2.0057
2.2608
1.0375
[rel]
[ppm]

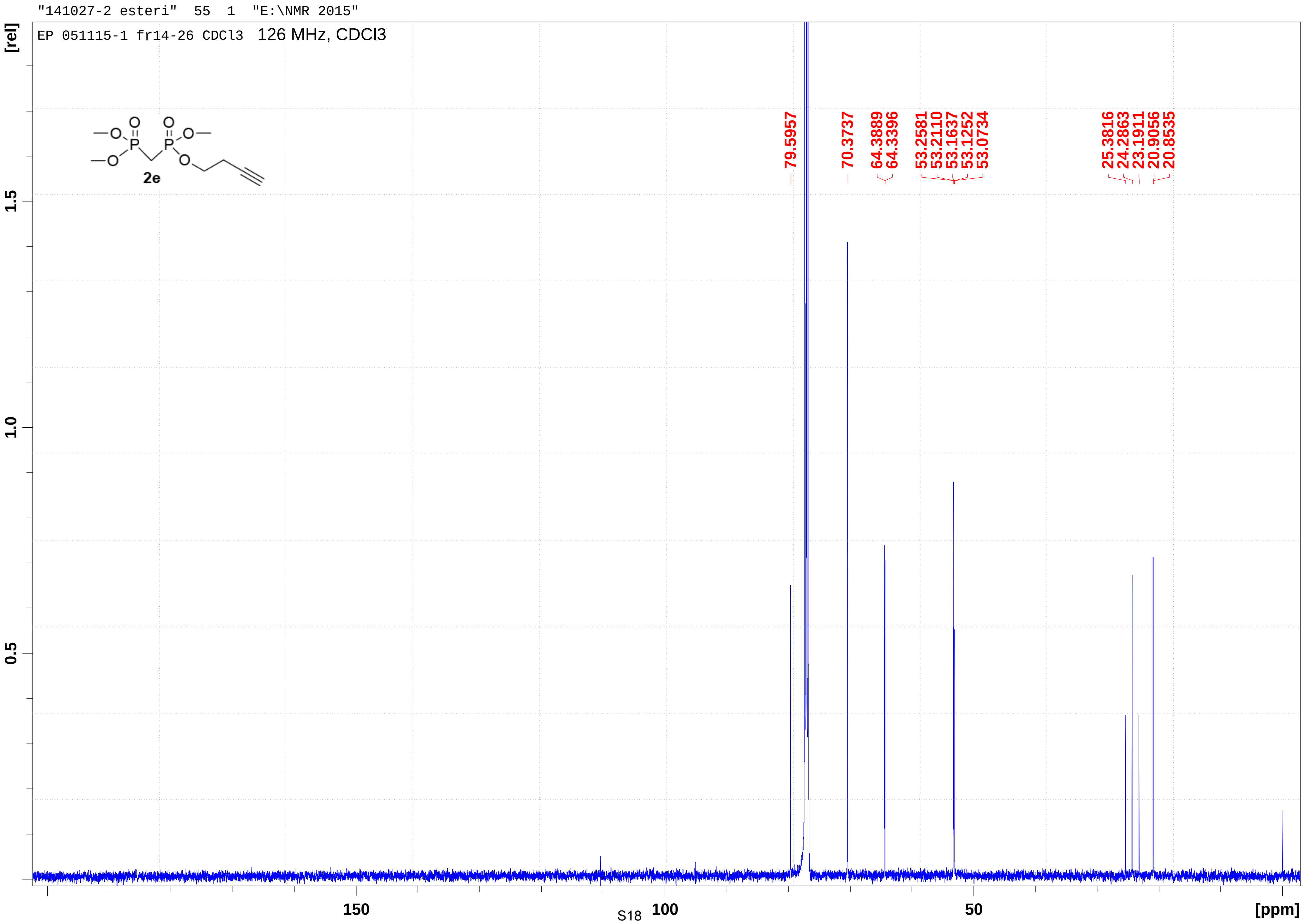
"141027-2 esteri" 55 1 "E:\NMR 2015"
EP 051115-1 fr14-26 CDCl3
126 MHz, CDCl3
2e
79.5957
70.3737
64.3889
64.3396
53.2581
53.2110
53.1637
53.1252
53.0734
25.3816
24.2863
23.1911
20.9056
20.8535
[rel]
1.5
1.0
0.5
150
100
50
[ppm]

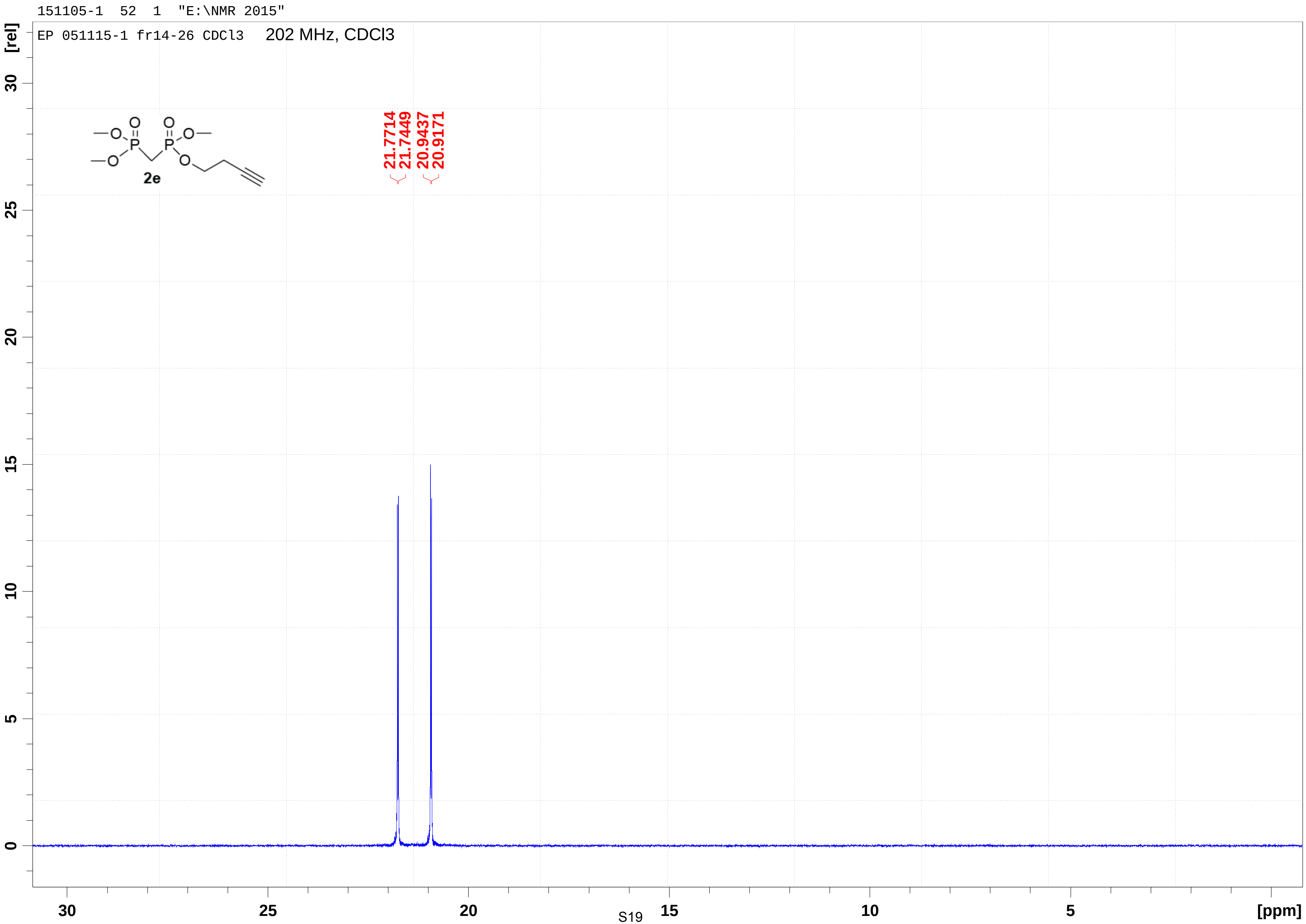
151105-1 52 1 "E:\NMR 2015"
EP 051115-1 fr14-26 CDCl3
202 MHz, CDCl3
2e
21.7714
21.7449
20.9437
20.9171
[rel]
[ppm]

MS-240414-1 22 1 "E:\NMR 2015"
PT/MS-240414-1 cdcl3 fr. 12-22 500 MHz, CDCl3

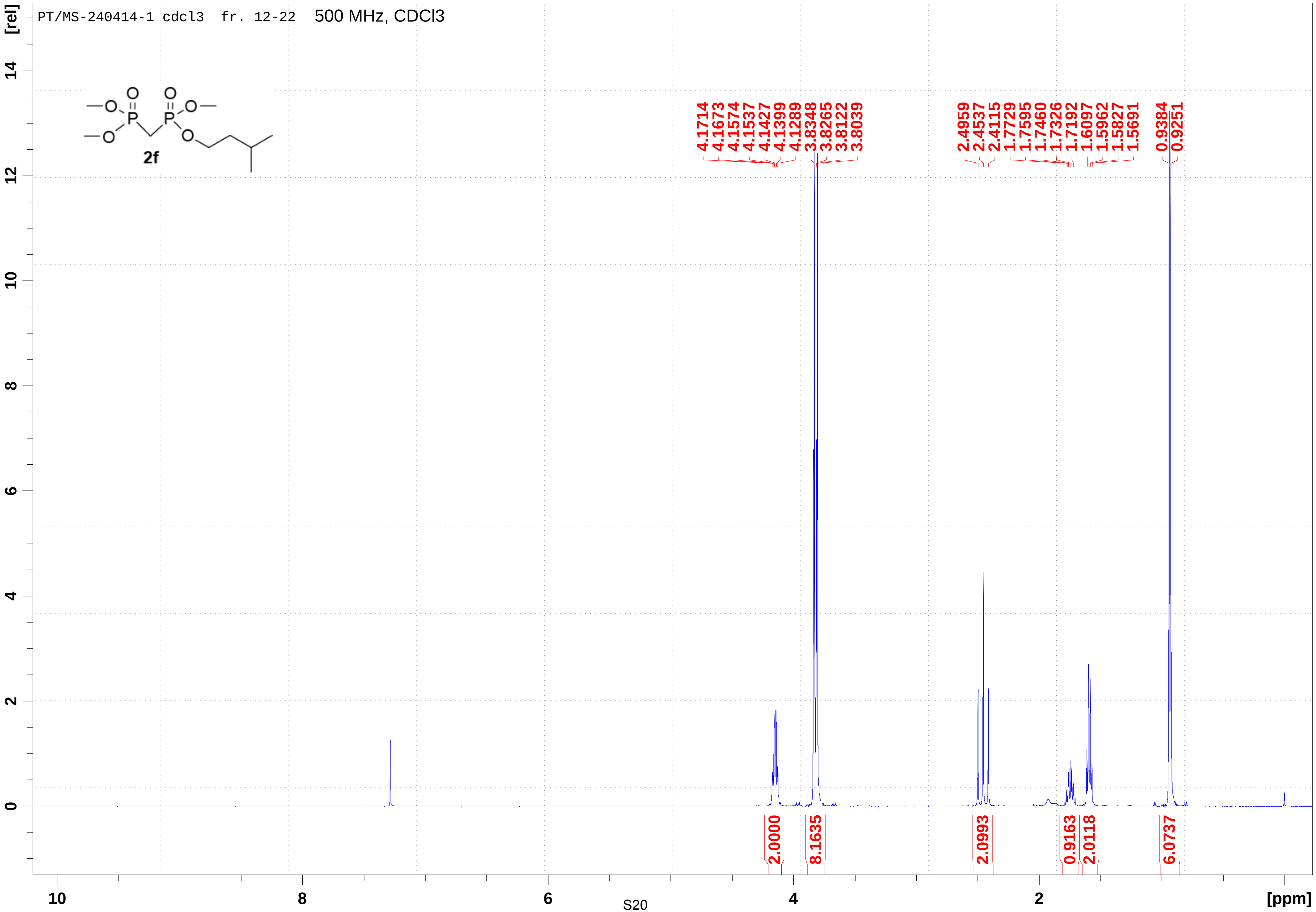

MS-240414-1 33 1 "E:\NMR 2015"

PT/MS-240414-1 cdcl3 fr. 12-22 126 MHz, CDCl3

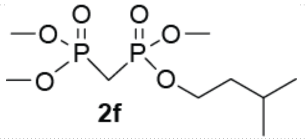

2f

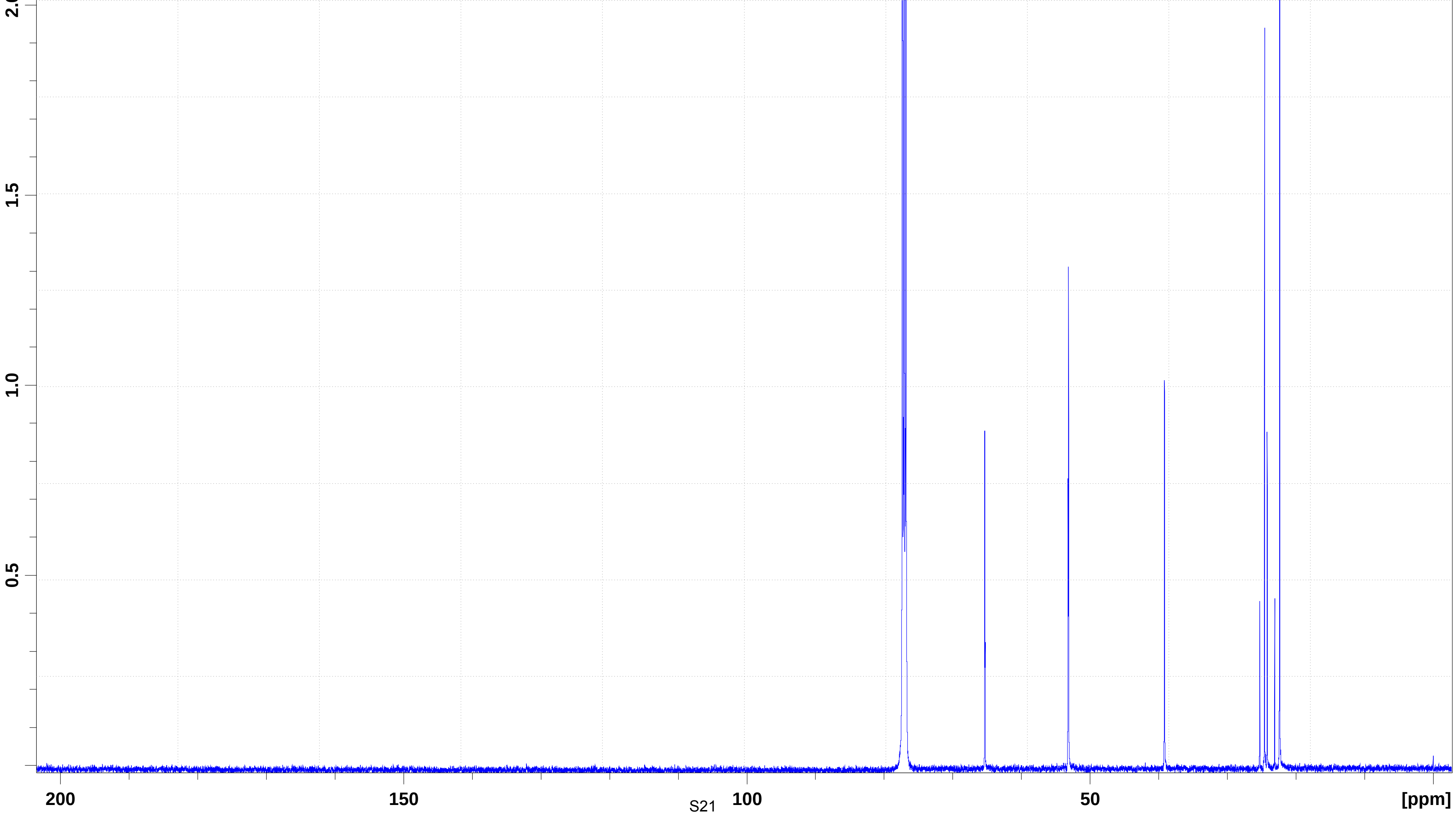

MS-240414-1 11 1 "E:\NMR 2015"
PT/MS-240414-1 cdcl3 fr. 12-22 202 MHz, CDCl3

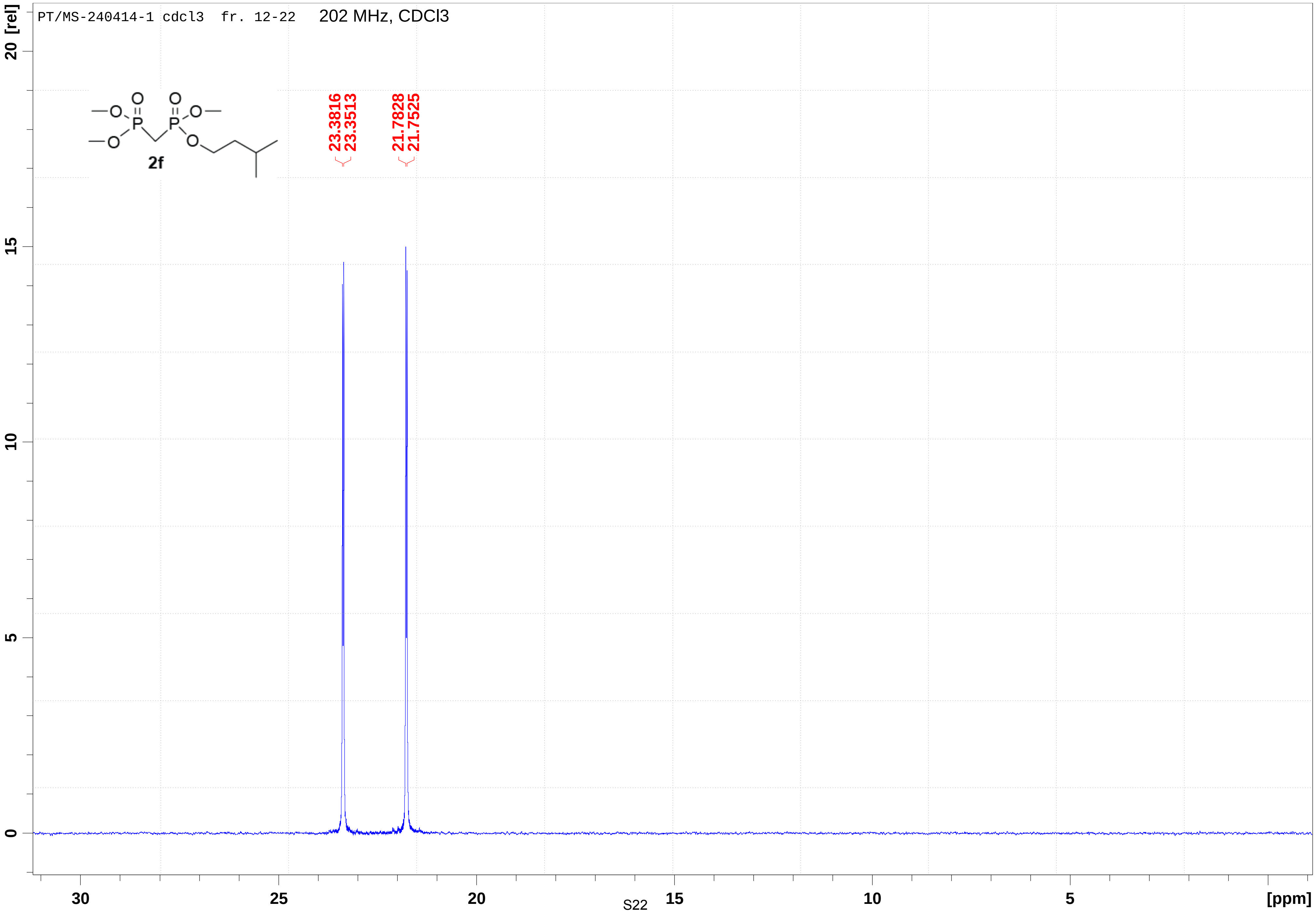

EPNL-081215-1 10 1 "E:\NMR 2015"

EPNL-081215-1 CDCl3 600 MHz, CDCl3

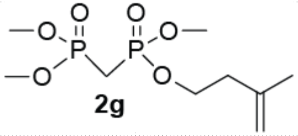

2g

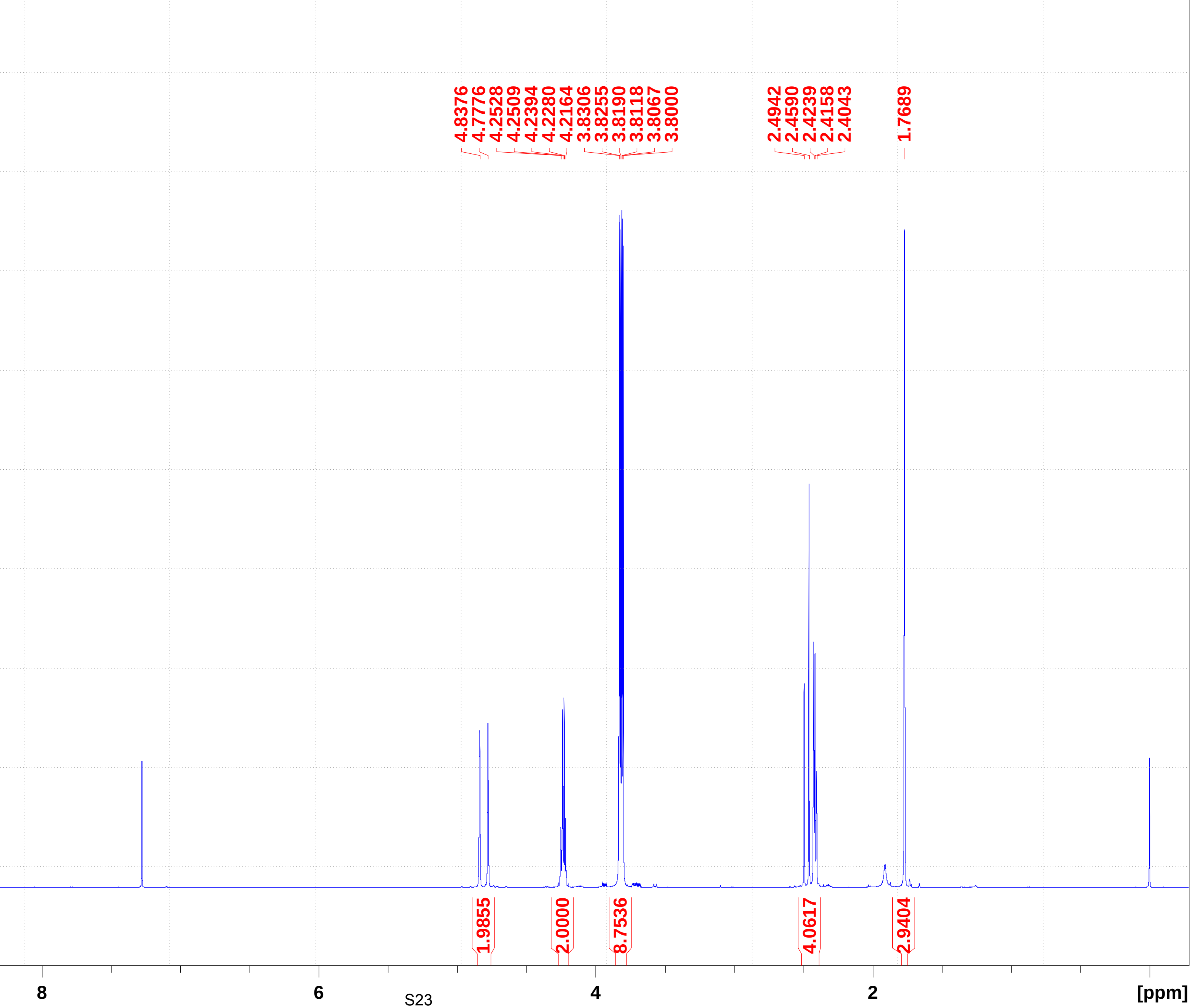

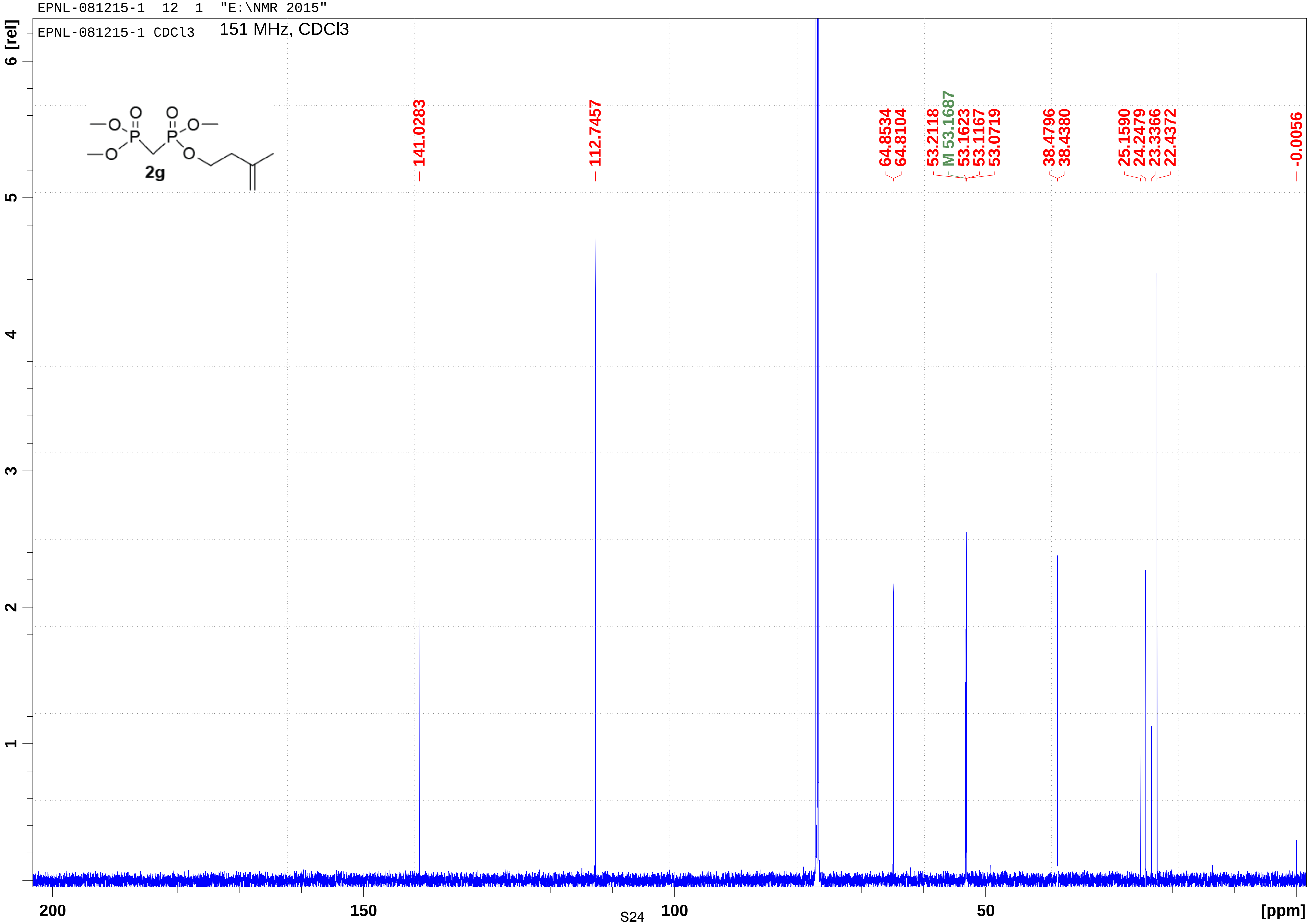
EPNL-081215-1 12 1 "E:\NMR 2015"
EPNL-081215-1 CDCl3
151 MHz, CDCl3
2g
141.0283
112.7457
64.8534
64.8104
53.2118
M 53.1687
53.1623
53.1167
53.0719
38.4796
38.4380
25.1590
24.2479
23.3366
22.4372
-0.0056
6 [rel]
5
4
3
2
1
200
150
100
50
[ppm]

EPNL-081215-1 11 1 "E:\NMR 2015"

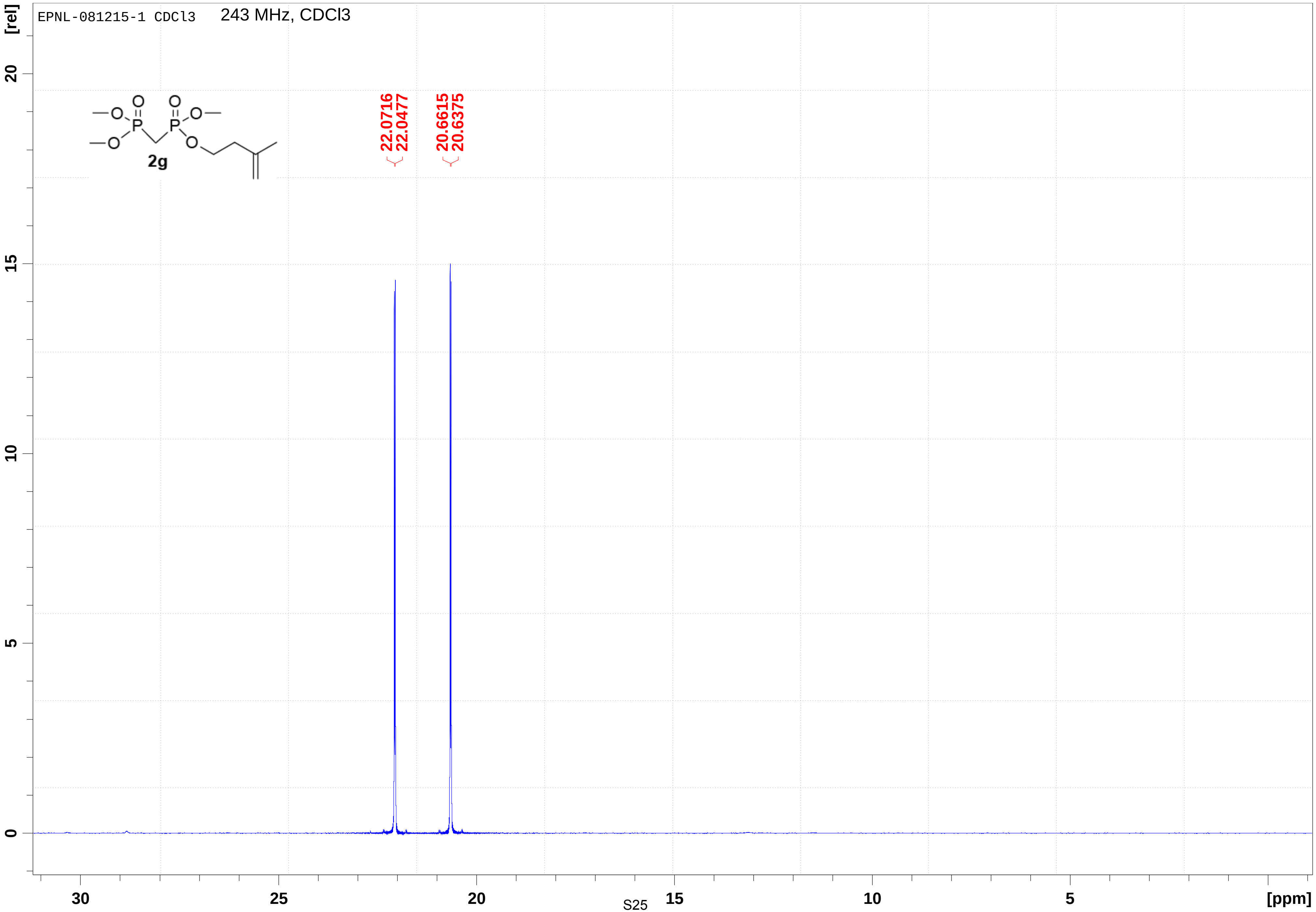

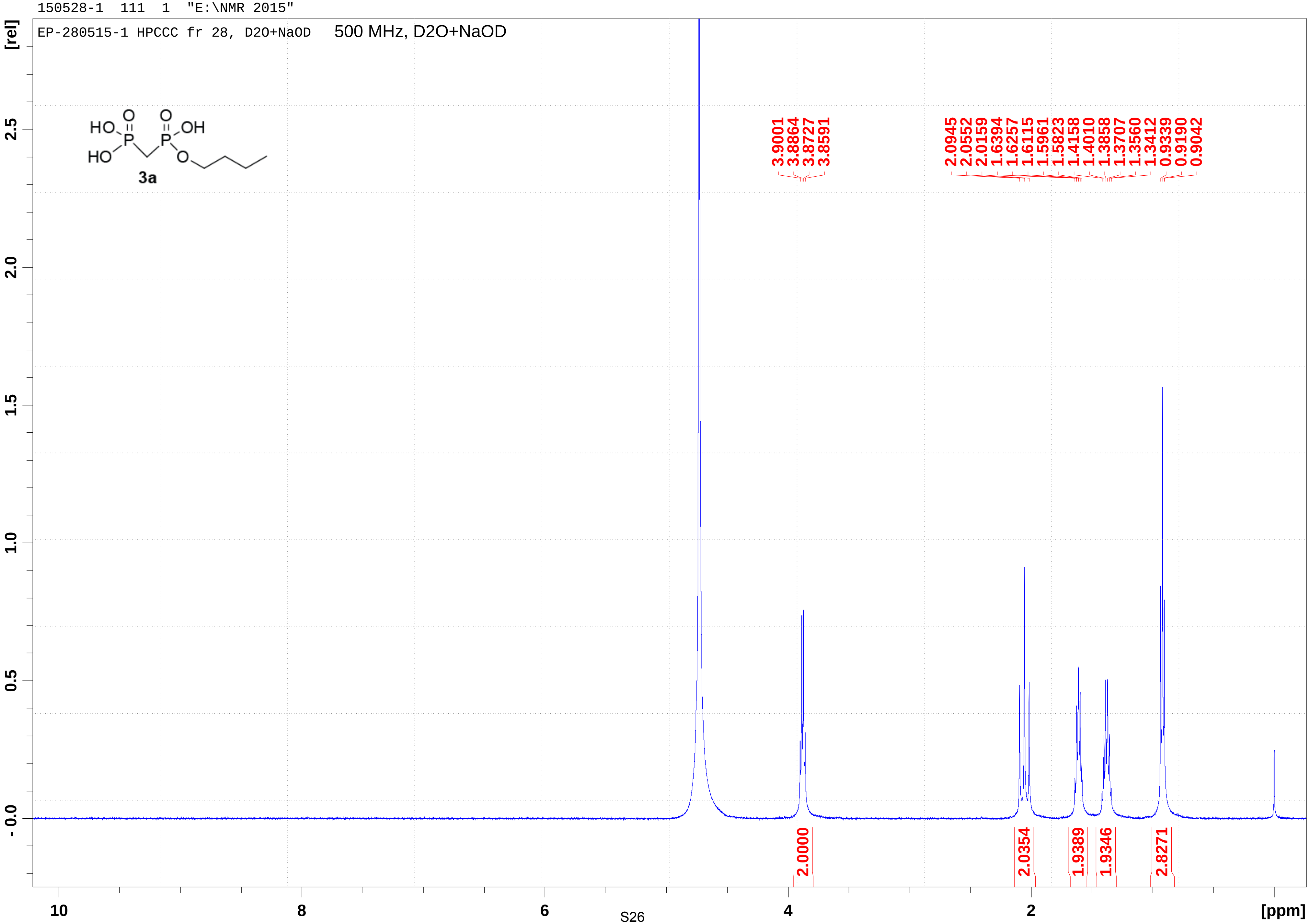
150528-1 111 1 "E:\NMR 2015"
EP-280515-1 HPCCC fr 28, D2O+NaOD
500 MHz, D2O+NaOD
HO
HO
P
O
P
O
OH
O
3a
3.9001
3.8864
3.8727
3.8591
2.0945
2.0552
2.0159
1.6394
1.6257
1.6115
1.5961
1.5823
1.4158
1.4010
1.3858
1.3707
1.3560
1.3412
0.9339
0.9190
0.9042
2.0000
2.0354
1.9389
1.9346
2.8271
[rel]
2.5
2.0
1.5
1.0
0.5
- 0.0
10
8
6
4
2
[ppm]

150528-1 116 1 "E:\NMR 2015"

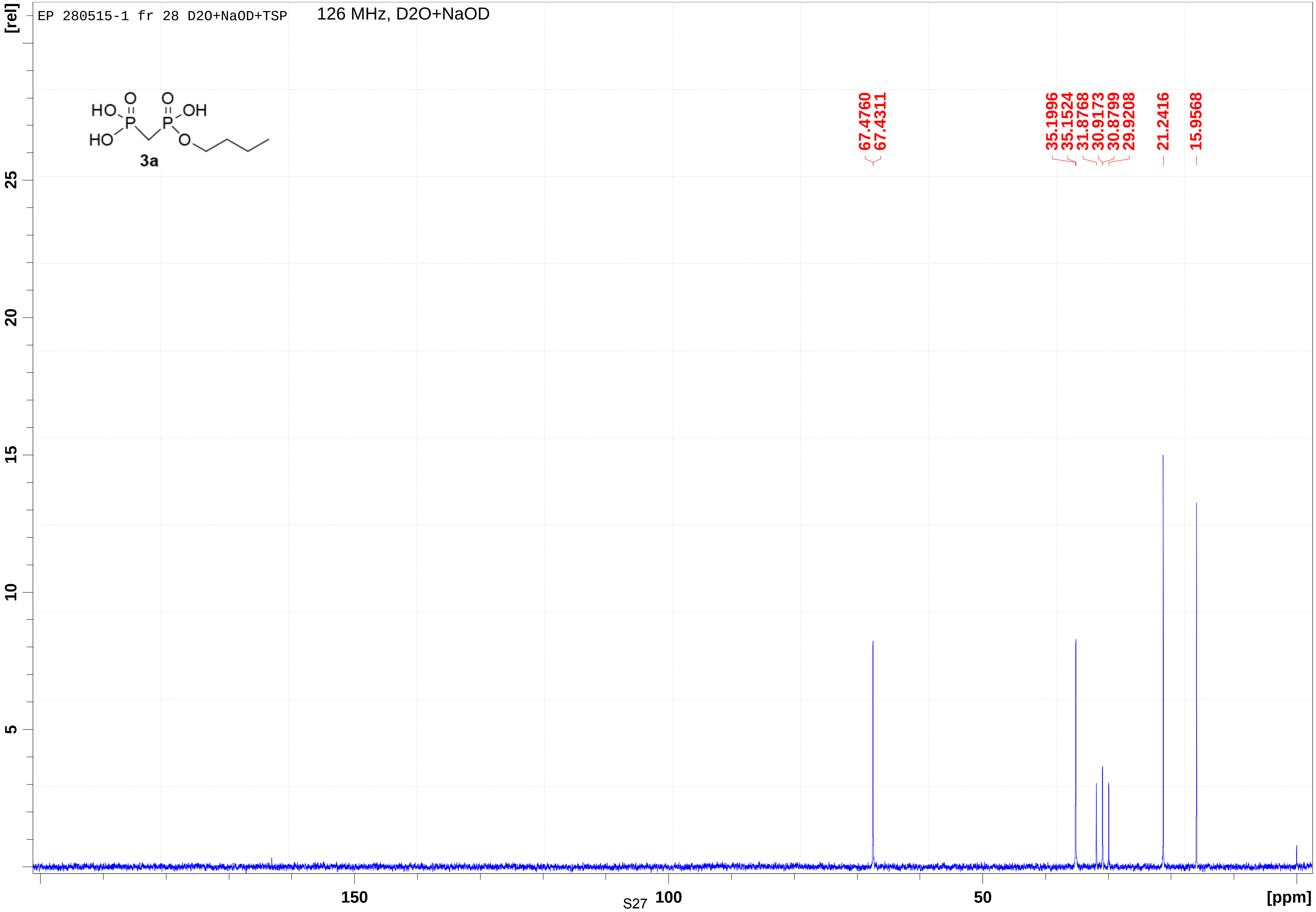

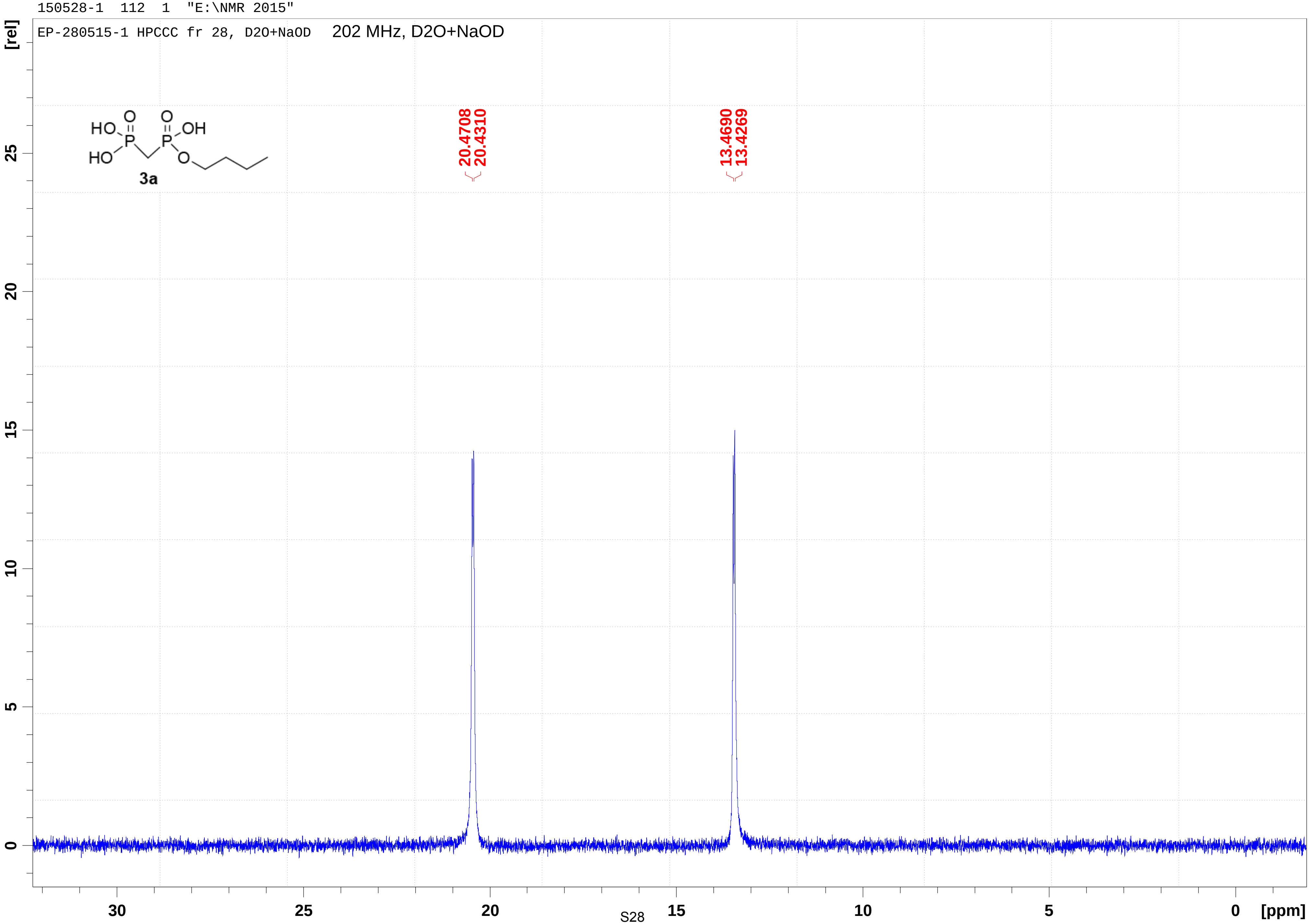

150528-1 112 1 "E:\NMR 2015"
EP-280515-1 HPCCC fr 28, D2O+NaOD
202 MHz, D2O+NaOD
3a
20.4708
20.4310
13.4690
13.4269
[rel]
[ppm]

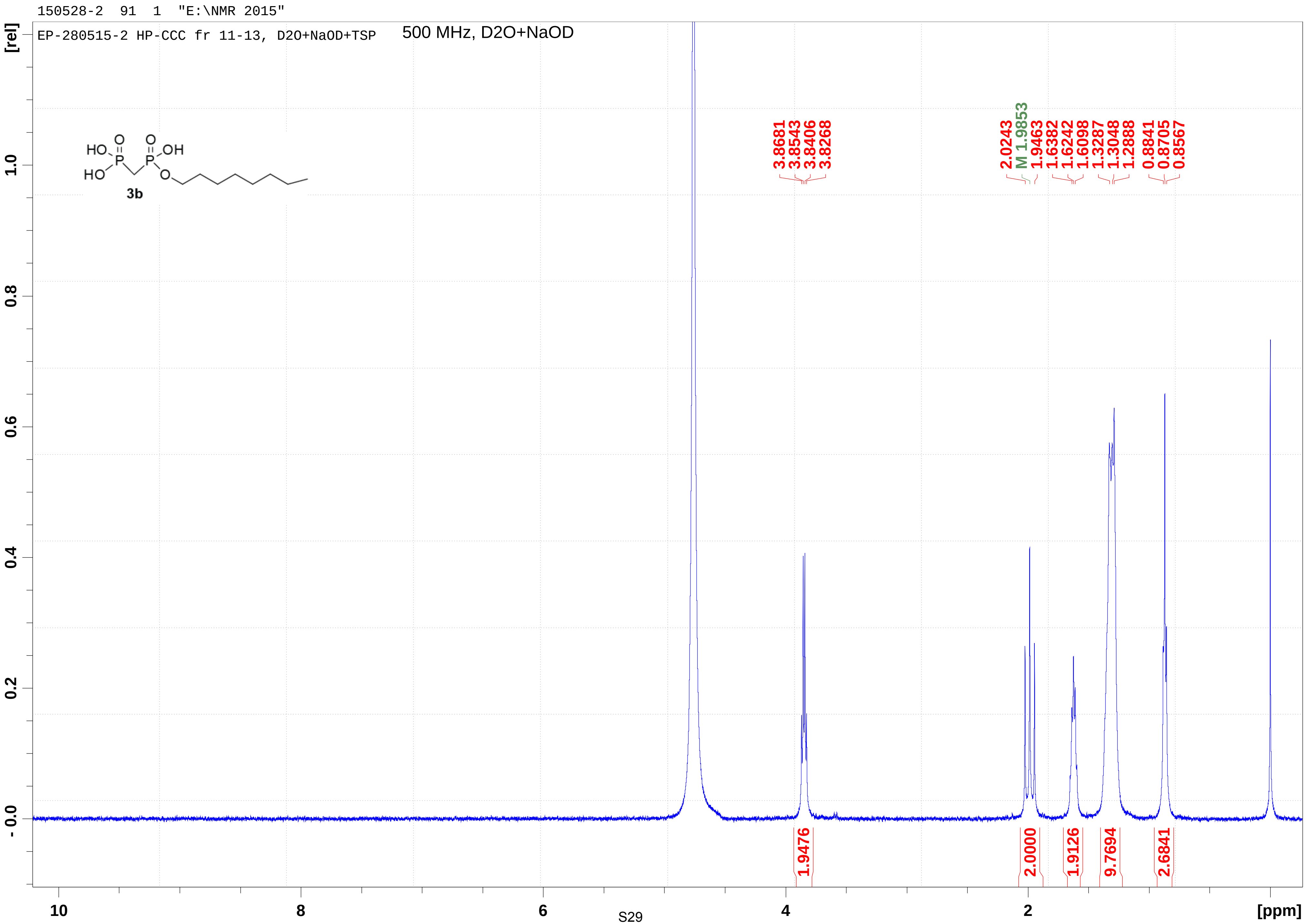

150528-2 91 1 "E:\NMR 2015"
EP-280515-2 HP-CCC fr 11-13, D2O+NaOD+TSP
500 MHz, D2O+NaOD
3b
3.8681
3.8543
3.8406
3.8268
2.0243
M 1.9853
1.9463
1.6382
1.6242
1.6098
1.3287
1.3048
1.2888
0.8841
0.8705
0.8567
1.9476
2.0000
1.9126
9.7694
2.6841
[rel]
[ppm]

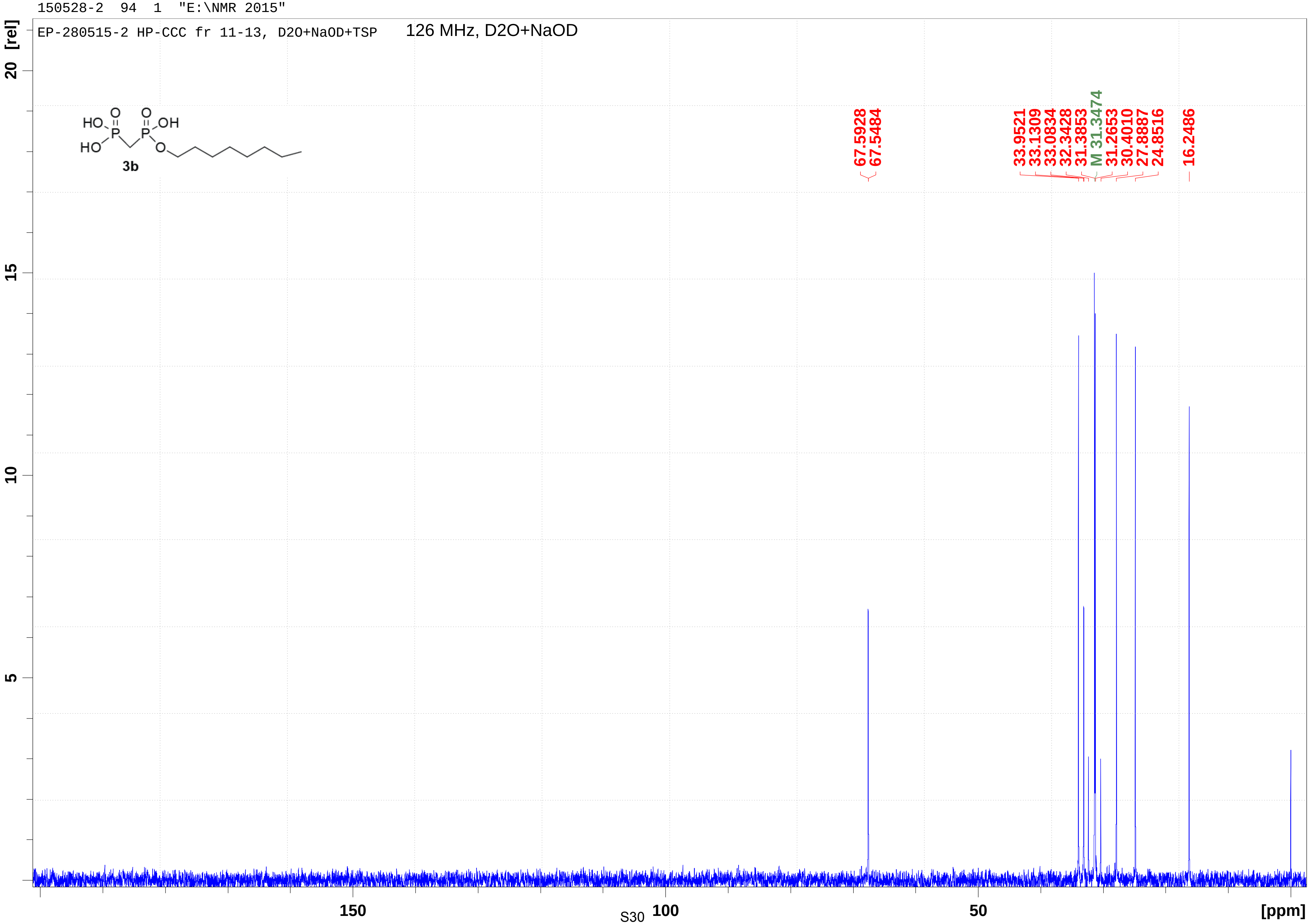
150528-2 94 1 "E:\NMR 2015"
EP-280515-2 HP-CCC fr 11-13, D2O+NaOD+TSP
126 MHz, D2O+NaOD
HO
HO
O
P
O
P
OH
O
3b
67.5928
67.5484
33.9521
33.1309
33.0834
32.3428
31.3853
M 31.3474
31.2653
30.4010
27.8887
24.8516
16.2486
20 [rel]
15
10
5
150
100
50
[ppm]

150528-2 92 1 "E:\NMR 2015"

EP-280515-2 HP-CCC fr 11-13, D2O+NaOD+TSP 202 MHz, D2O+NaOD

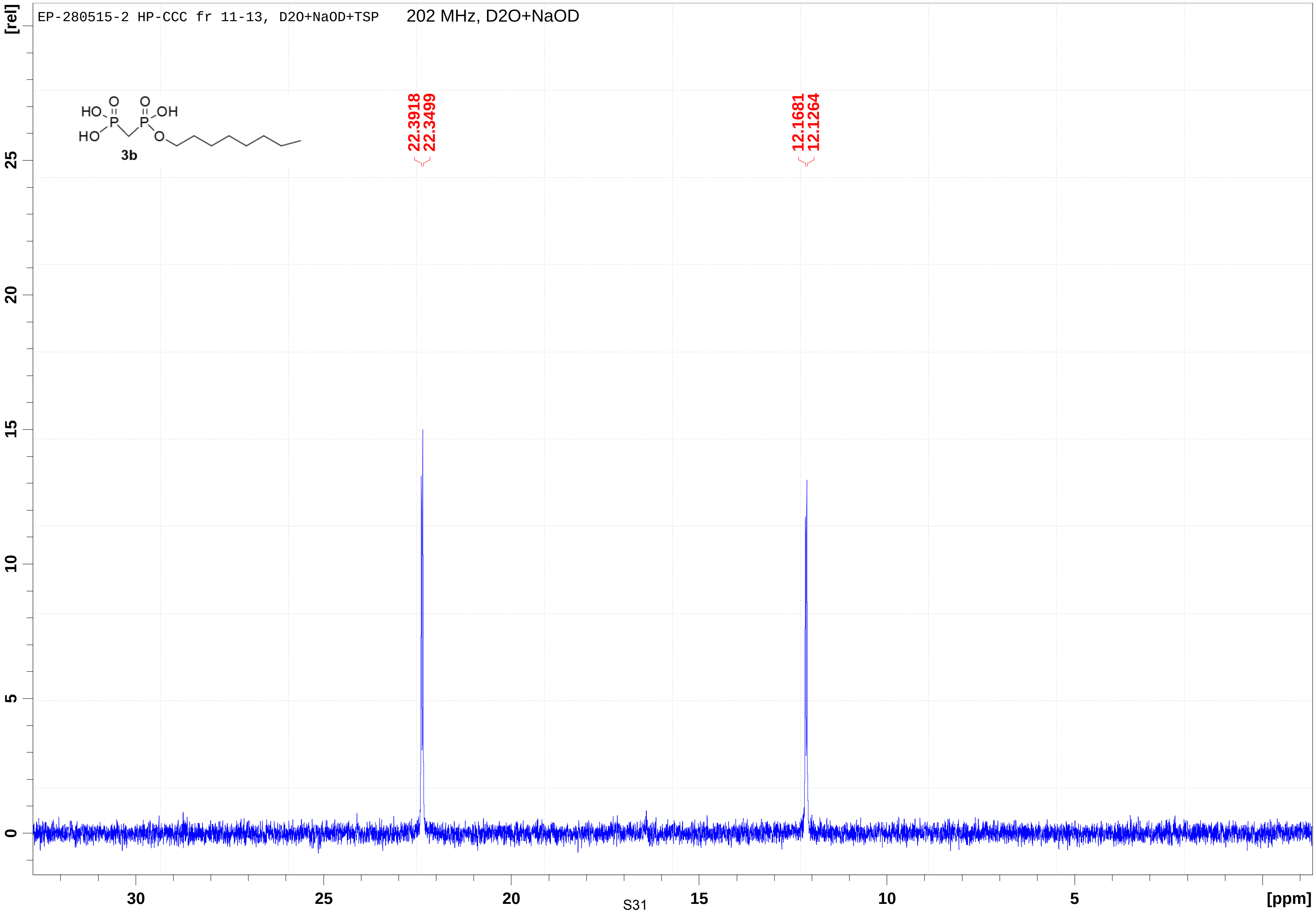

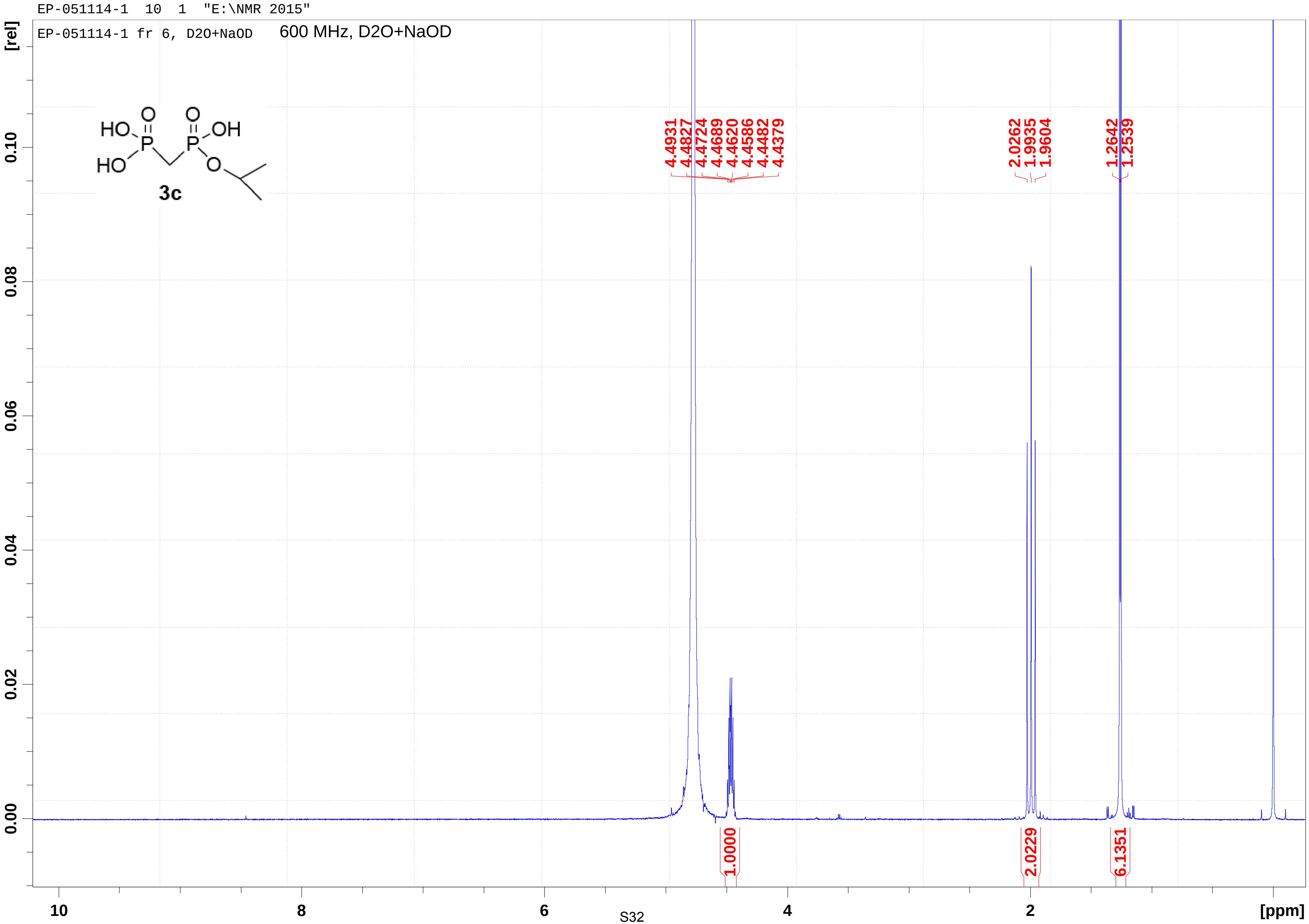

EP-051114-1 10 1 "E:\NMR 2015"
EP-051114-1 fr 6, D2O+NaOD
600 MHz, D2O+NaOD
3c
4.4931
4.4827
4.4724
4.4689
4.4620
4.4586
4.4482
4.4379
2.0262
1.9935
1.9604
1.2642
1.2539
1.0000
2.0229
6.1351
[rel]
[ppm]

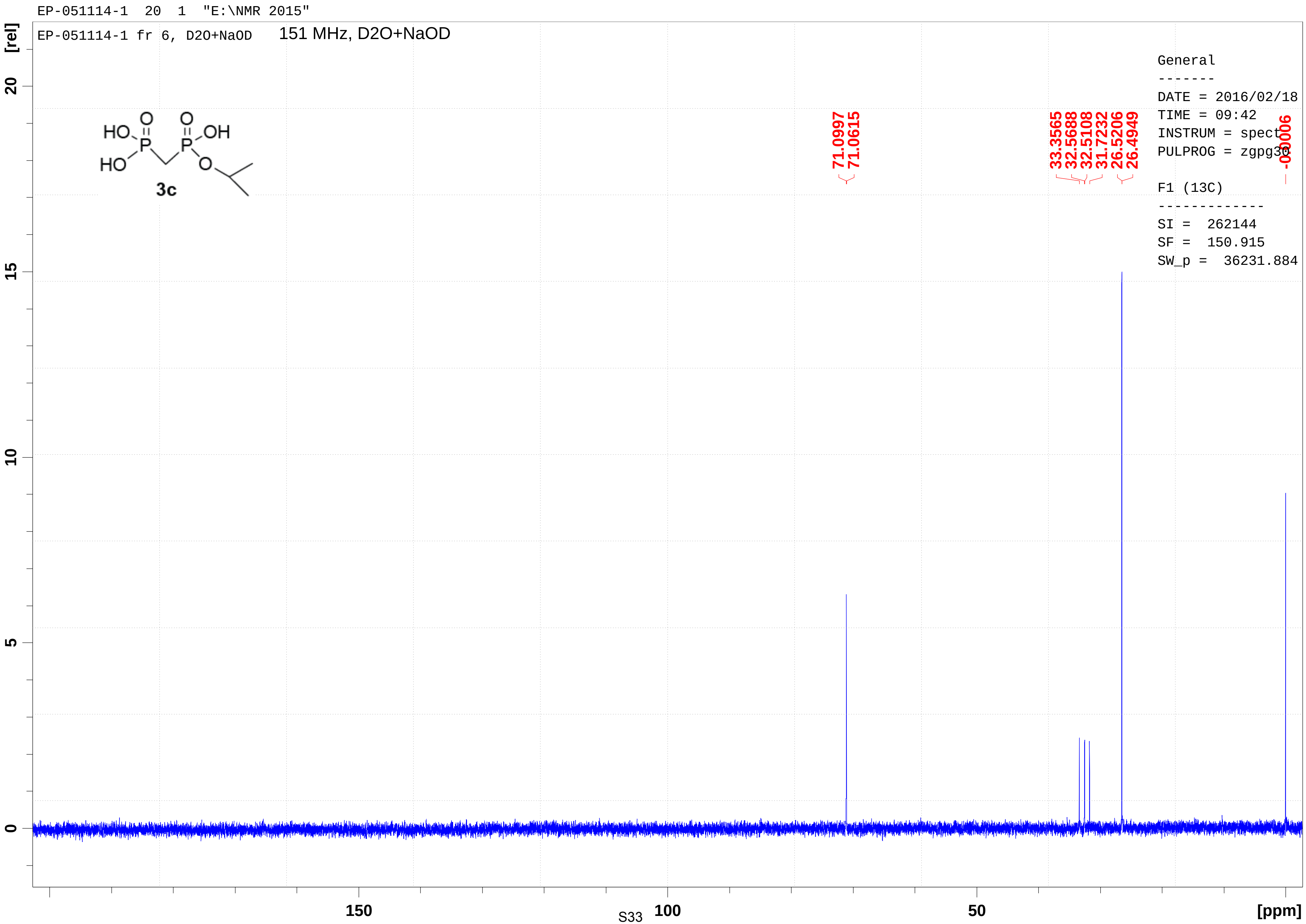

EP-051114-1 20 1 "E:\NMR 2015"
EP-051114-1 fr 6, D2O+NaOD
151 MHz, D2O+NaOD
3c
71.0997
71.0615
33.3565
32.5688
32.5108
31.7232
26.5206
26.4949
-0.0006
General
DATE = 2016/02/18
TIME = 09:42
INSTRUM = spect
PULPROG = zgpg30
F1 (13C)
SI = 262144
SF = 150.915
SW_p = 36231.884
[rel]
[ppm]

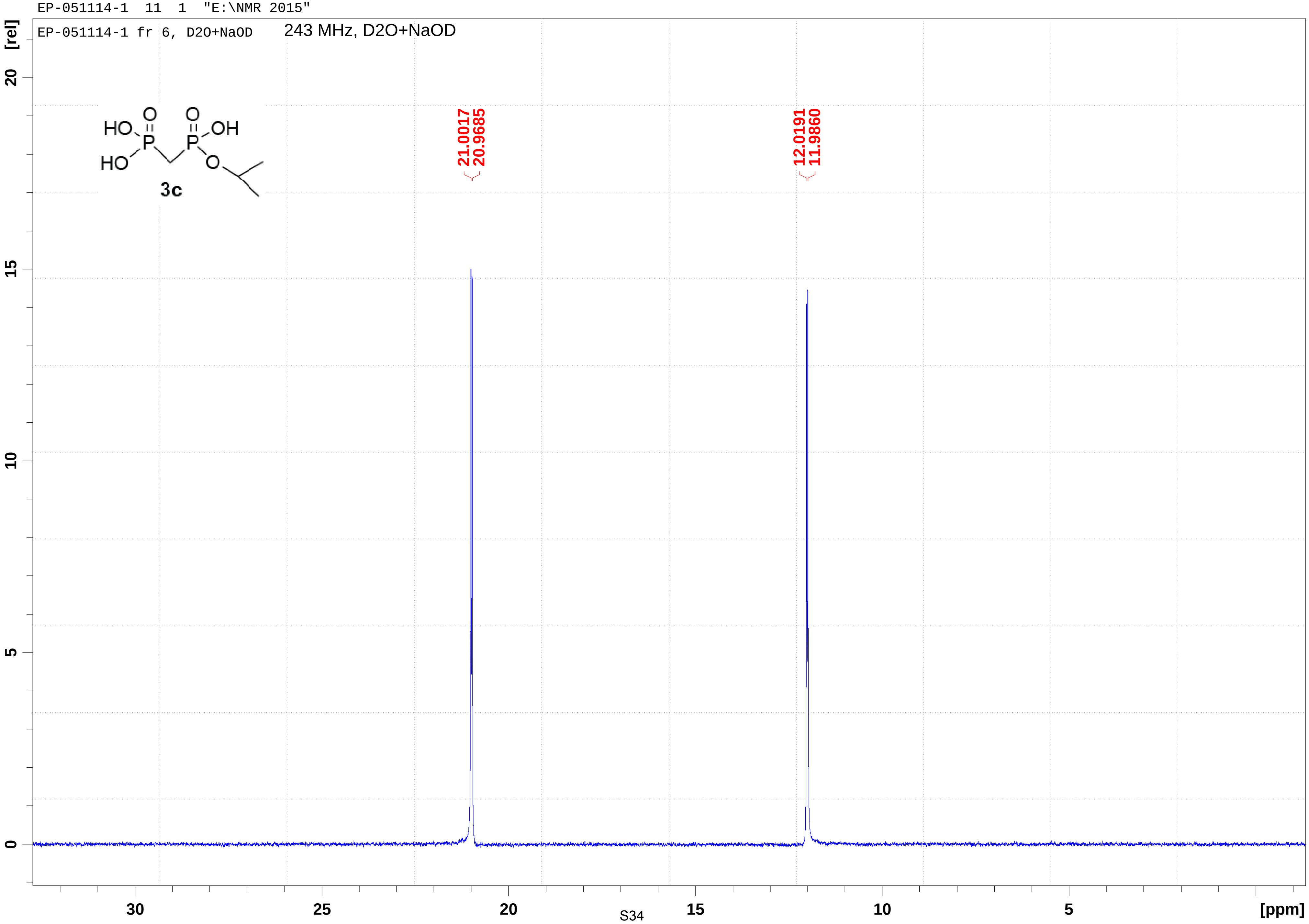
EP-051114-1 11 1 "E:\NMR 2015"
EP-051114-1 fr 6, D2O+NaOD
243 MHz, D2O+NaOD
HO
HO
O
O
P
P
OH
O
3c
21.0017
20.9685
12.0191
11.9860
[rel]
20
15
10
5
0
30
25
20
15
10
5
[ppm]

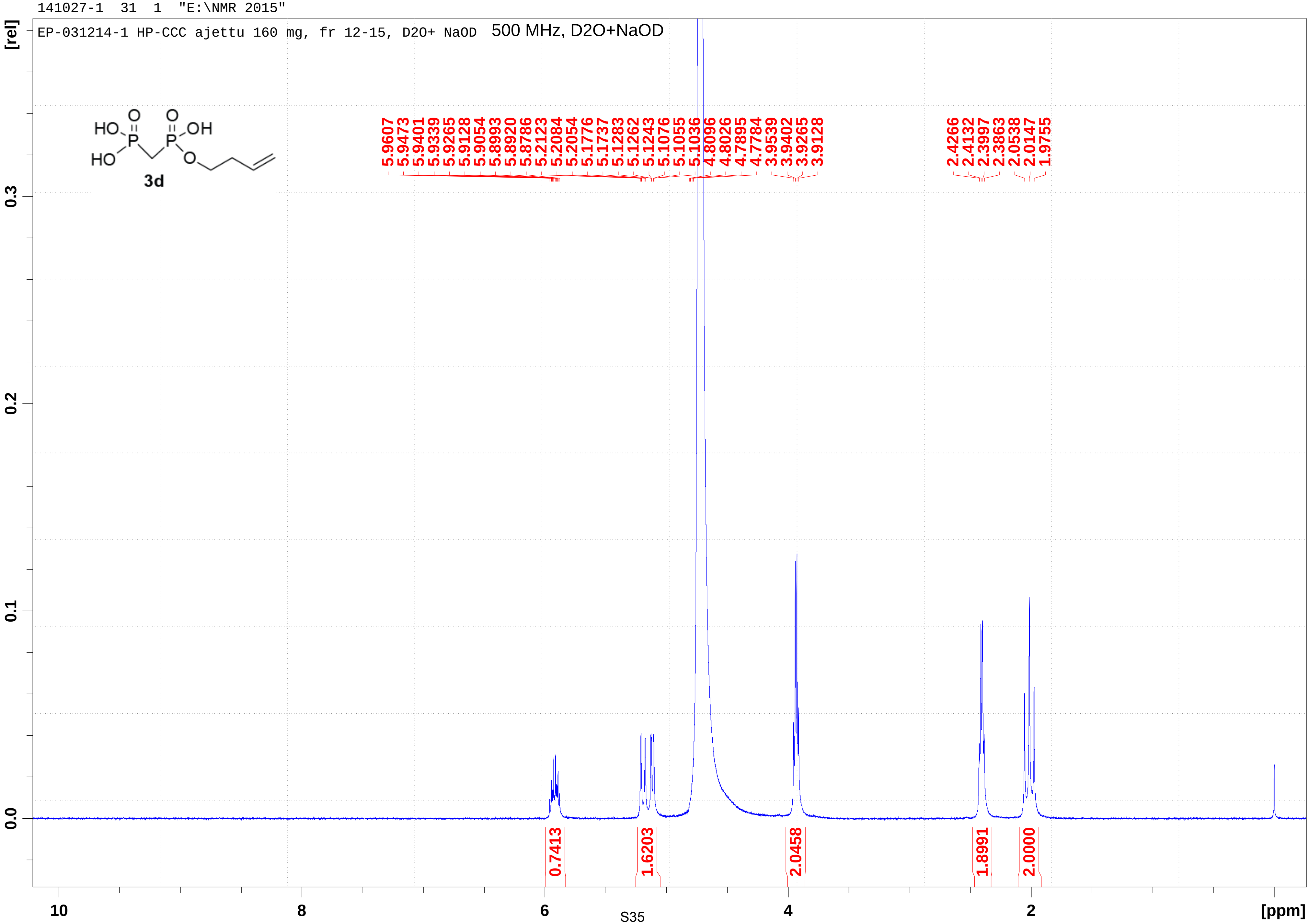
141027-1 31 1 "E:\NMR 2015"
EP-031214-1 HP-CCC ajettu 160 mg, fr 12-15, D2O+ NaOD 500 MHz, D2O+NaOD
3d
5.9607 5.9473 5.9401 5.9339 5.9265 5.9128 5.9054 5.8993 5.8920 5.8786 5.2123 5.2084 5.2054 5.1776 5.1737 5.1283 5.1262 5.1243 5.1076 5.1055 5.1036 4.8096 4.8026 4.7895 4.7784 3.9539 3.9402 3.9265 3.9128
2.4266 2.4132 2.3997 2.3863 2.0538 2.0147 1.9755
0.7413
1.6203
2.0458
1.8991
2.0000
[rel]
0.0
0.1
0.2
0.3
10
8
6
4
2
[ppm]

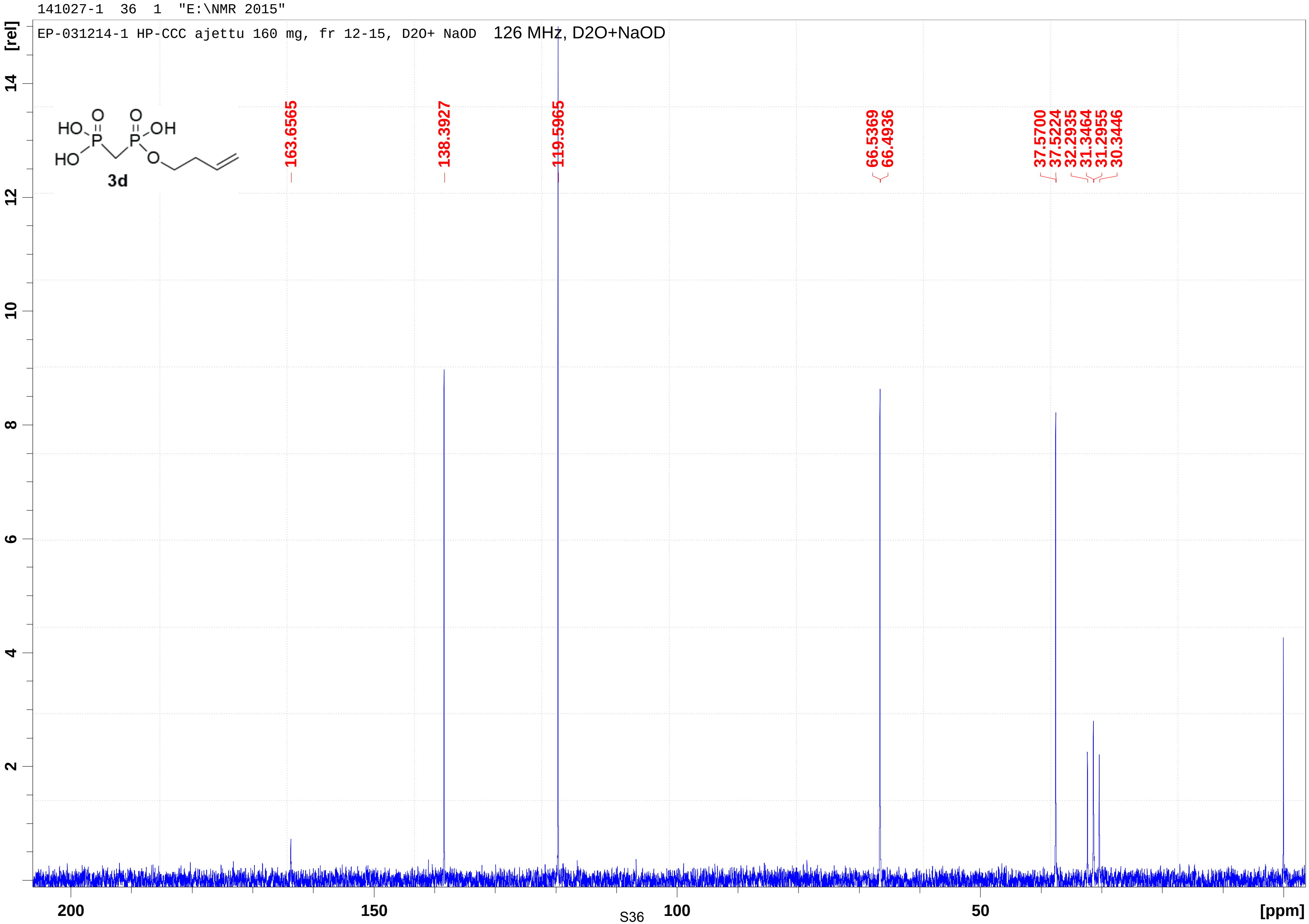
141027-1 36 1 "E:\NMR 2015"
EP-031214-1 HP-CCC ajettu 160 mg, fr 12-15, D2O+ NaOD
126 MHz, D2O+NaOD
HO
HO
O
P
O
P
OH
O
3d
163.6565
138.3927
119.5965
66.5369
66.4936
37.5700
37.5224
32.2935
31.3464
31.2955
30.3446
[rel]
14
12
10
8
6
4
2
200
150
100
50
[ppm]

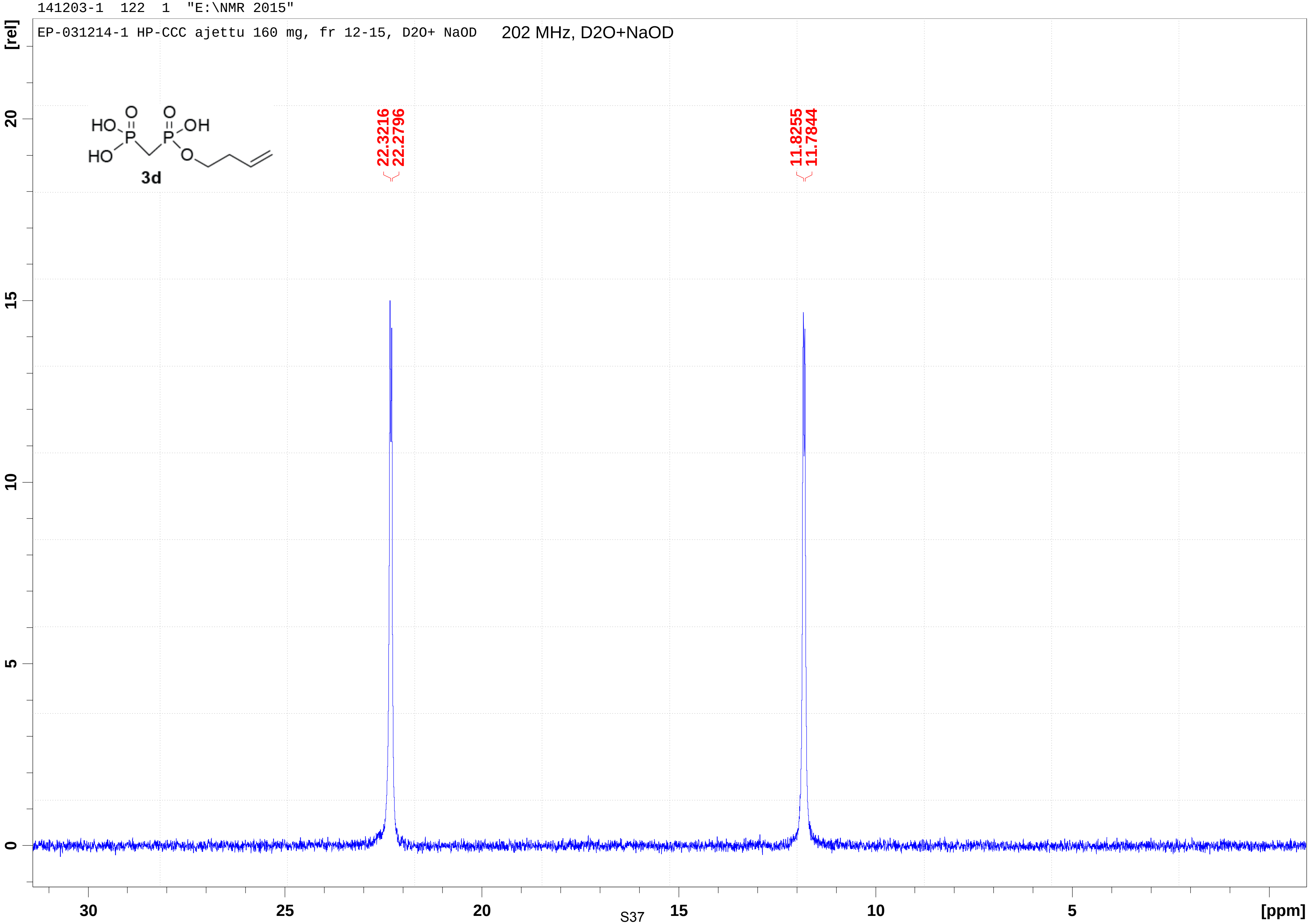
141203-1 122 1 "E:\NMR 2015"
EP-031214-1 HP-CCC ajettu 160 mg, fr 12-15, D2O+ NaOD
202 MHz, D2O+NaOD
3d
22.3216
22.2796
11.8255
11.7844
[rel]
[ppm]

EP-271014-2 20 1 "E:\NMR 2015"

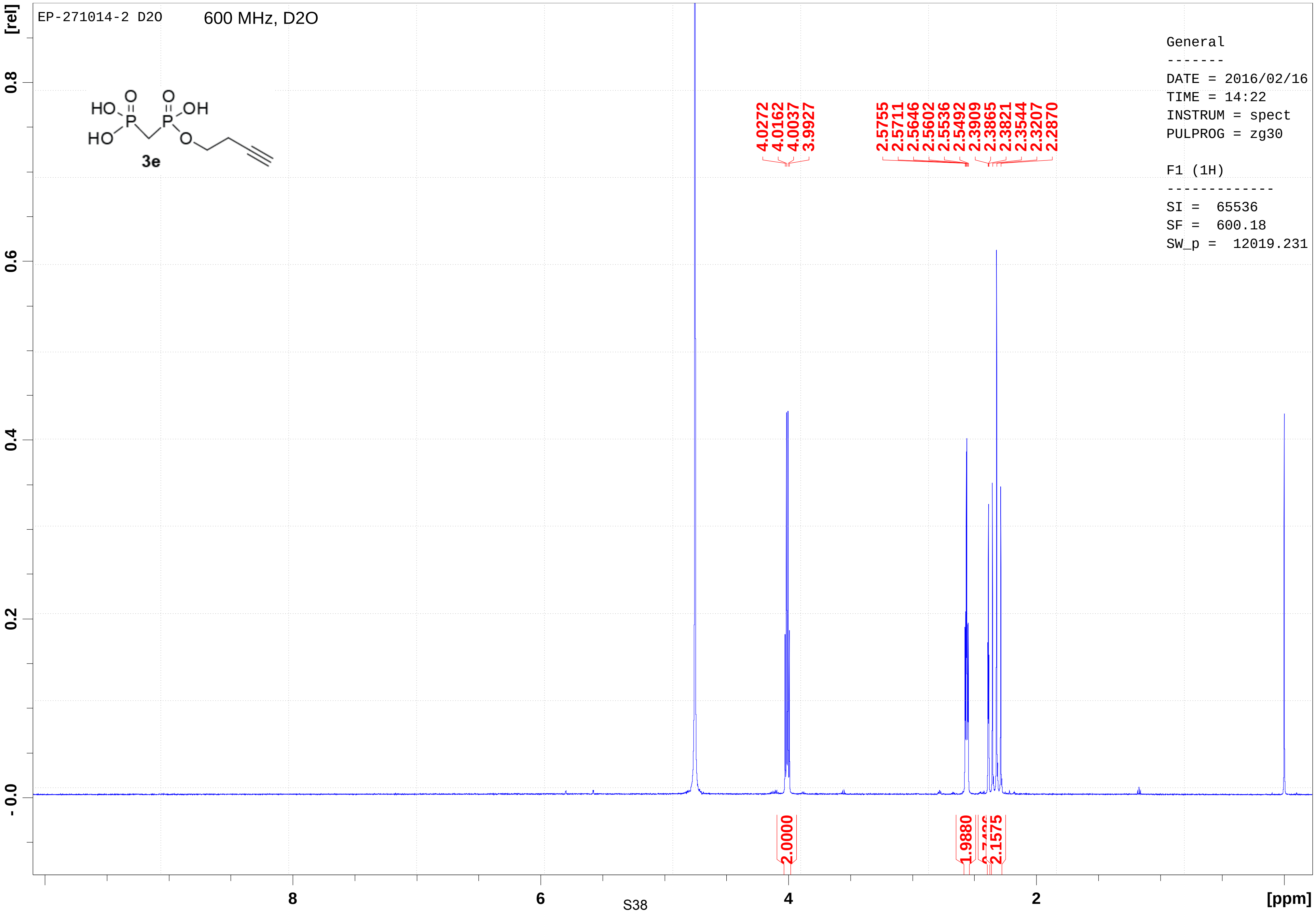

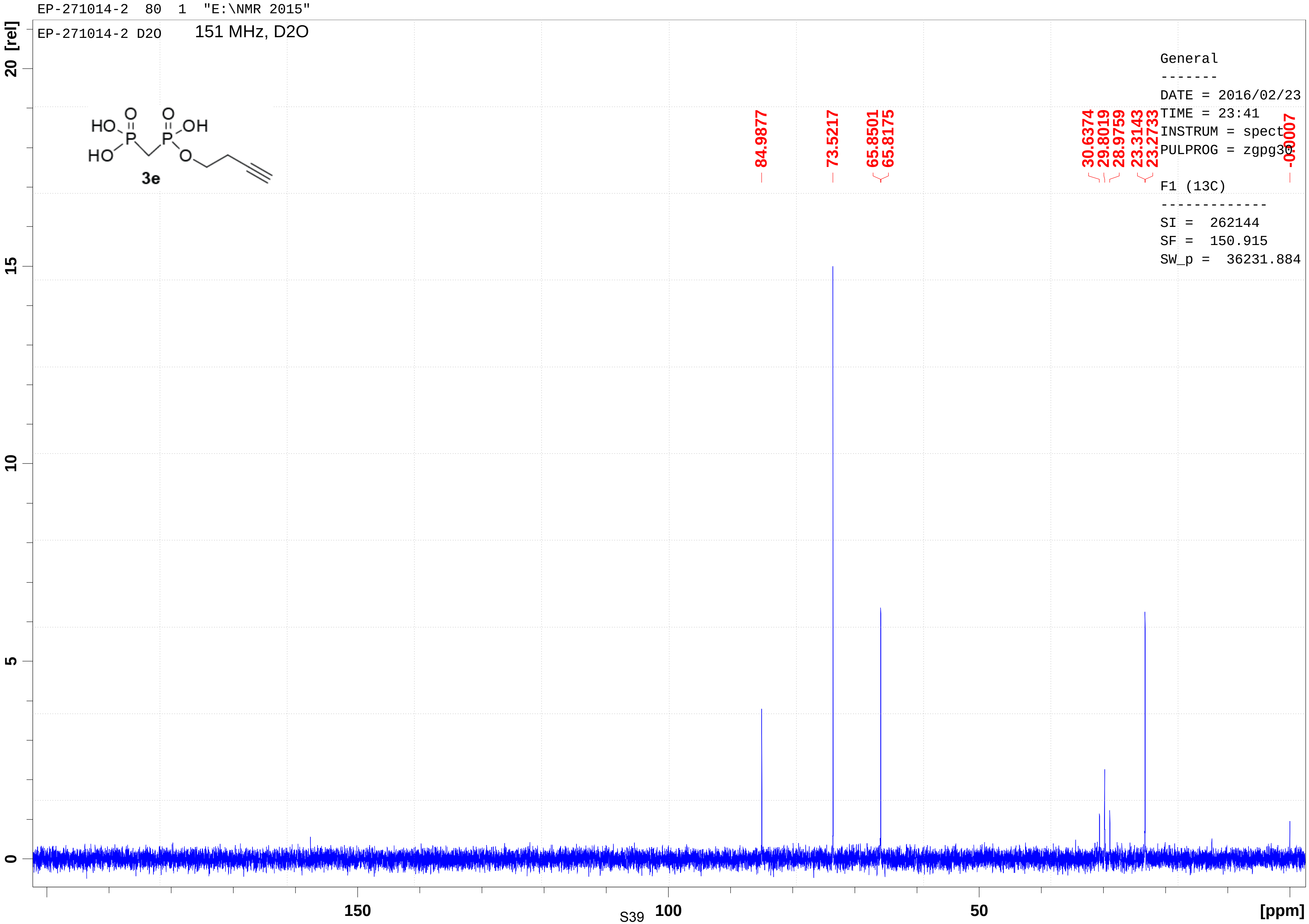
EP-271014-2 80 1 "E:\NMR 2015"
EP-271014-2 D2O
151 MHz, D2O
HO
HO
P
O
P
O
OH
O
3e
84.9877
73.5217
65.8501
65.8175
30.6374
29.8019
28.9759
23.3143
23.2733
-0.0007
General

DATE = 2016/02/23
TIME = 23:41
INSTRUM = spect
PULPROG = zgpg30
F1 (13C)

SI = 262144
SF = 150.915
SW_p = 36231.884
20 [rel]
15
10
5
0
150
100
50
[ppm]

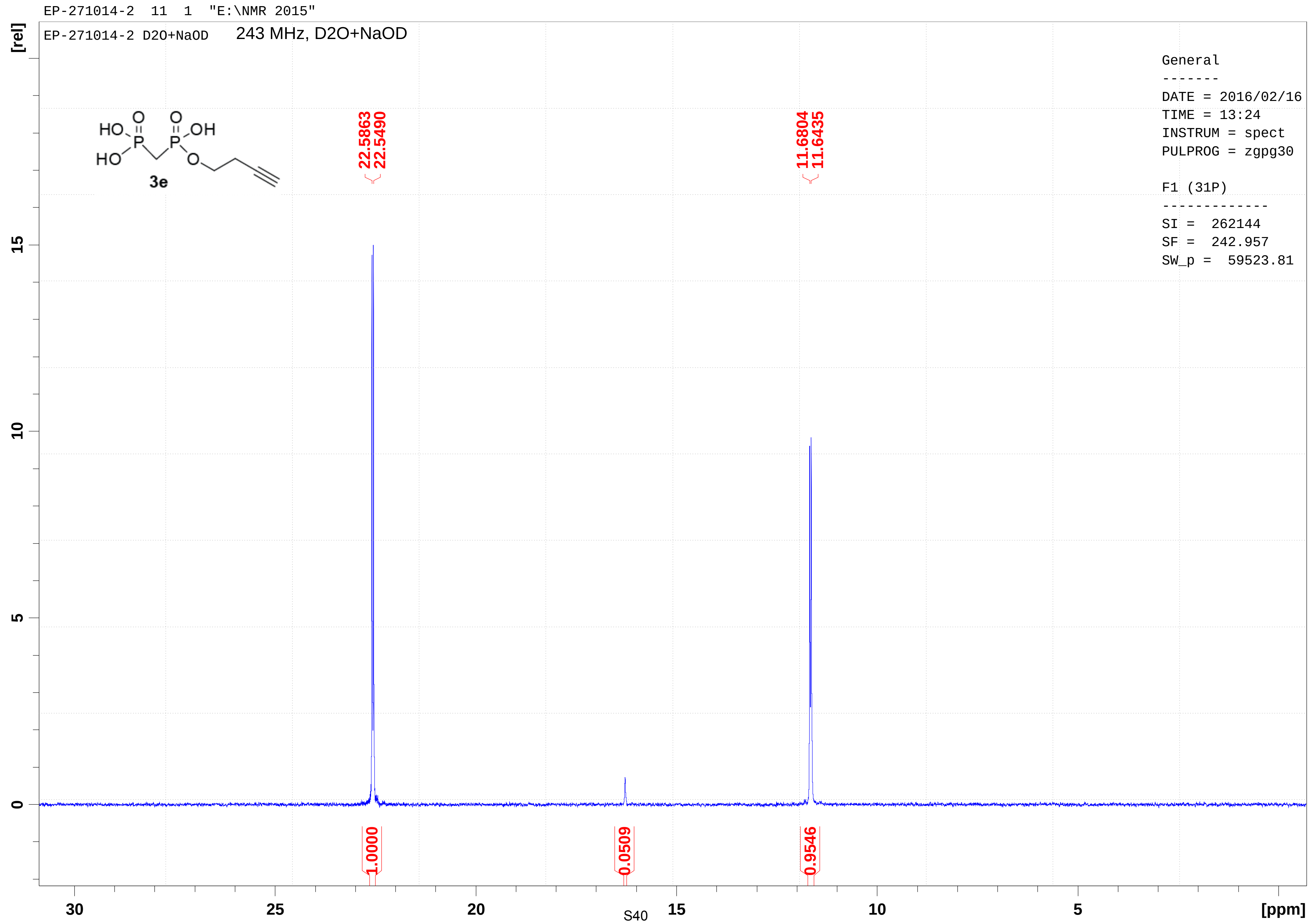
EP-271014-2 11 1 "E:\NMR 2015"
EP-271014-2 D2O+NaOD
243 MHz, D2O+NaOD
3e
22.5863
22.5490
11.6804
11.6435
General

DATE = 2016/02/16
TIME = 13:24
INSTRUM = spect
PULPROG = zgpg30
F1 (31P)

SI = 262144
SF = 242.957
SW_p = 59523.81
1.0000
0.0509
0.9546
[rel]
[ppm]

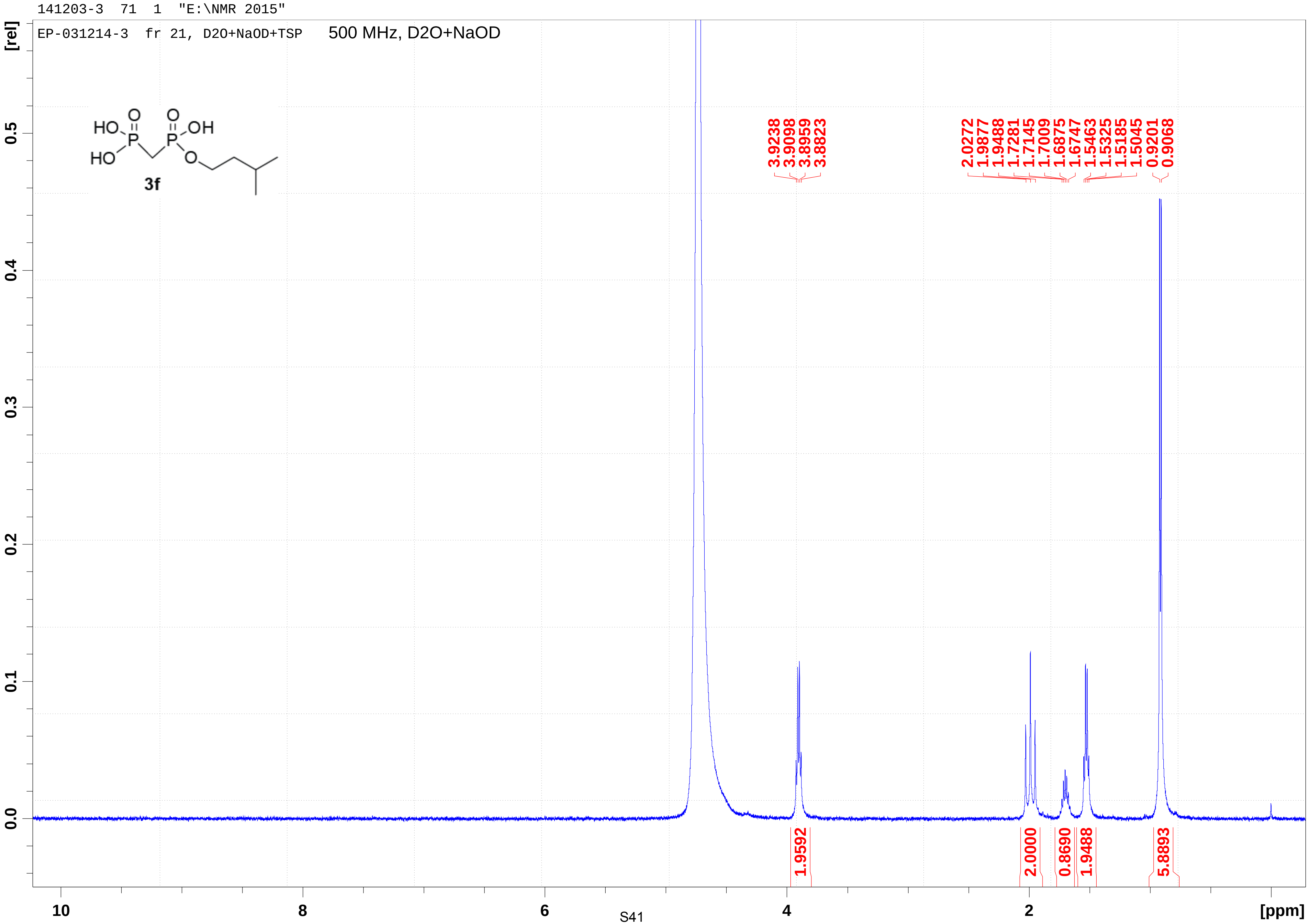

141203-3 71 1 "E:\NMR 2015"
EP-031214-3 fr 21, D2O+NaOD+TSP
500 MHz, D2O+NaOD
3f
3.9238
3.9098
3.8959
3.8823
2.0272
1.9877
1.9488
1.7281
1.7145
1.7009
1.6875
1.6747
1.5463
1.5325
1.5185
1.5045
0.9201
0.9068
1.9592
2.0000
0.8690
1.9488
5.8893
[rel]
[ppm]

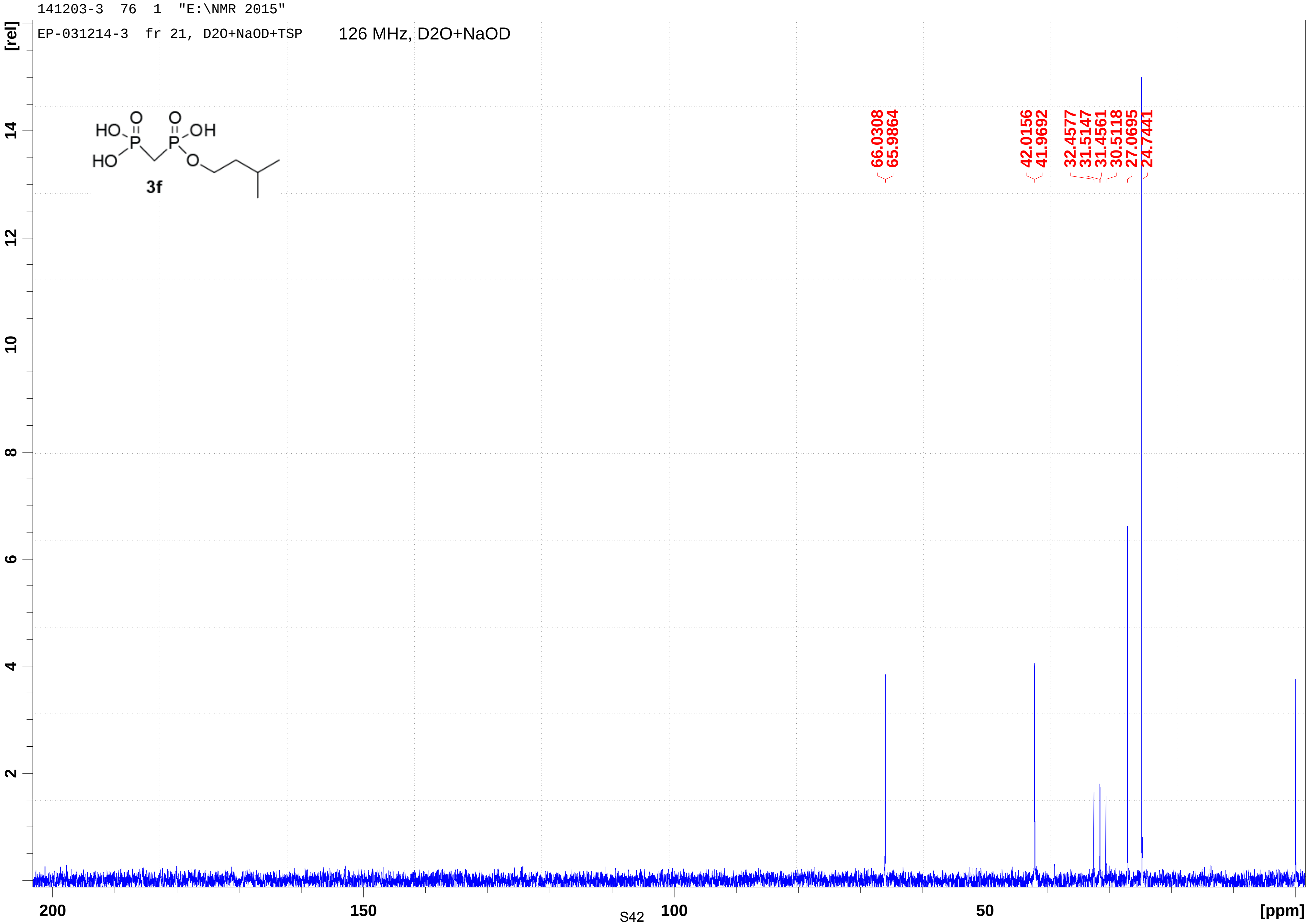
141203-3 76 1 "E:\NMR 2015"
EP-031214-3 fr 21, D2O+NaOD+TSP
126 MHz, D2O+NaOD
3f
HO
HO
OH
P
P
O
O
O
66.0308
65.9864
42.0156
41.9692
32.4577
31.5147
31.4561
30.5118
27.0695
24.7441
[rel]
14
12
10
8
6
4
2
200
150
100
50
[ppm]

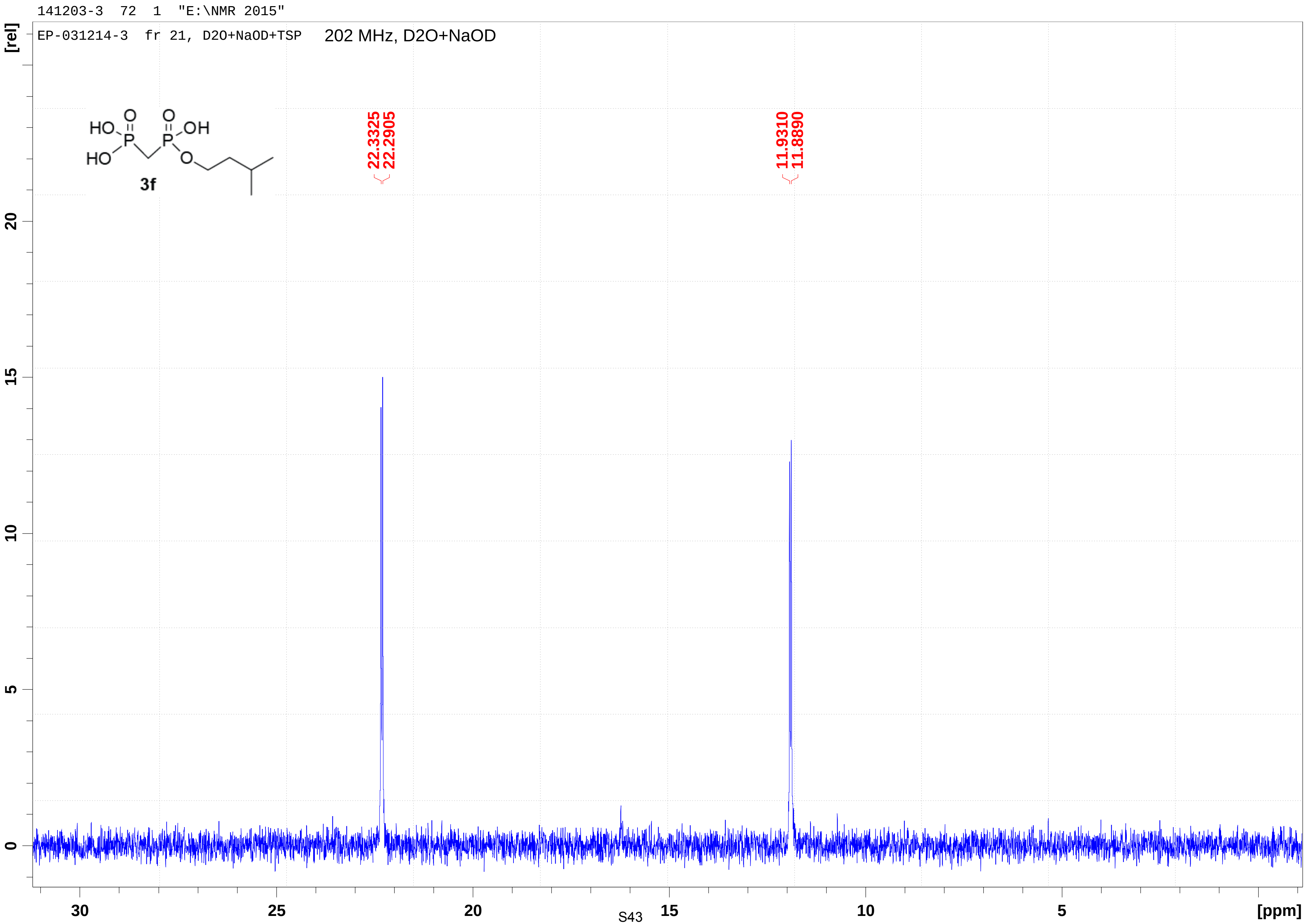
141203-3 72 1 "E:\NMR 2015"
EP-031214-3 fr 21, D2O+NaOD+TSP
202 MHz, D2O+NaOD
3f
22.3325
22.2905
11.9310
11.8890
[rel]
[ppm]

151021-1 71 1 "E:\NMR 2015"

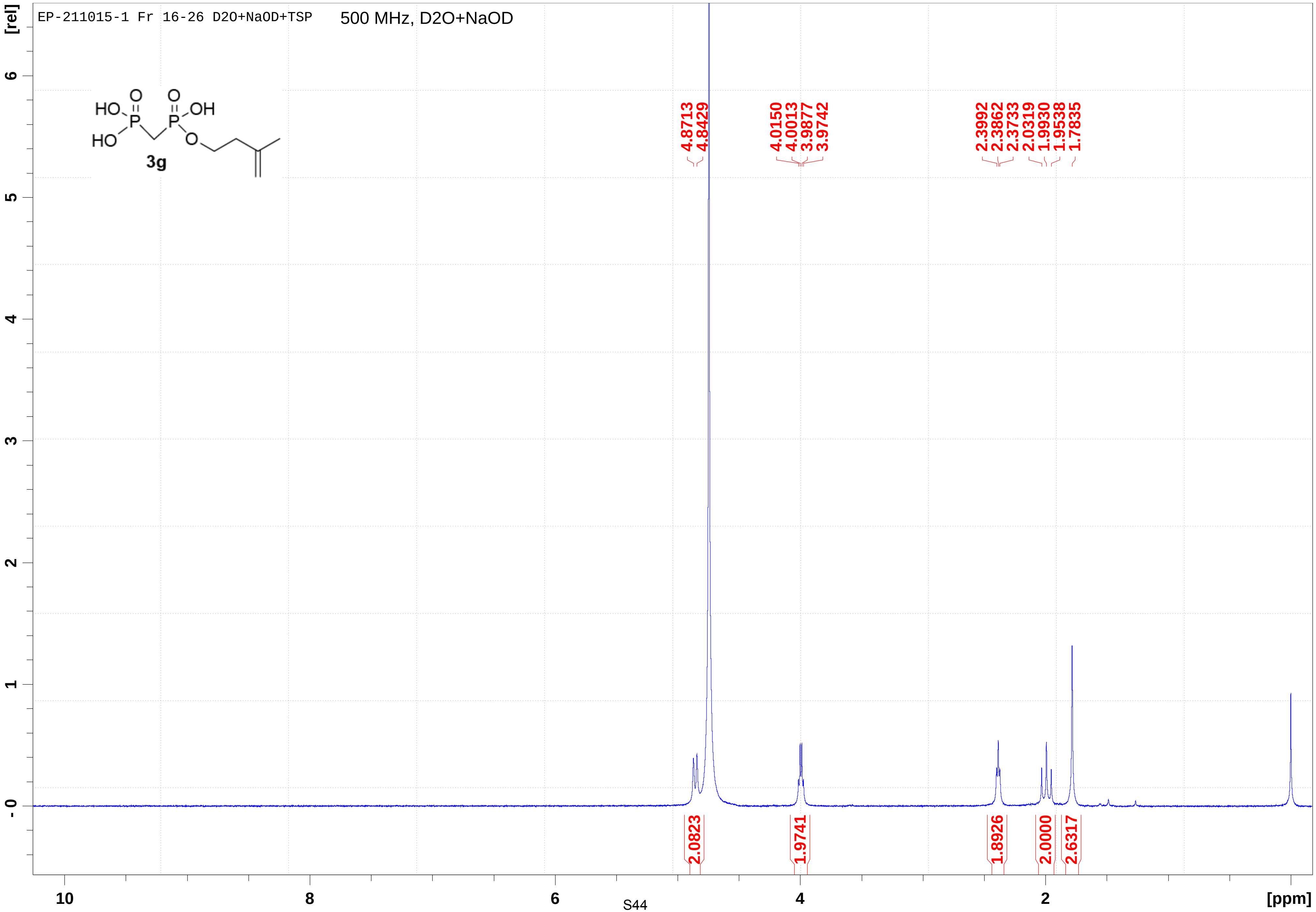

151021-1 73 1 "E:\NMR 2015"

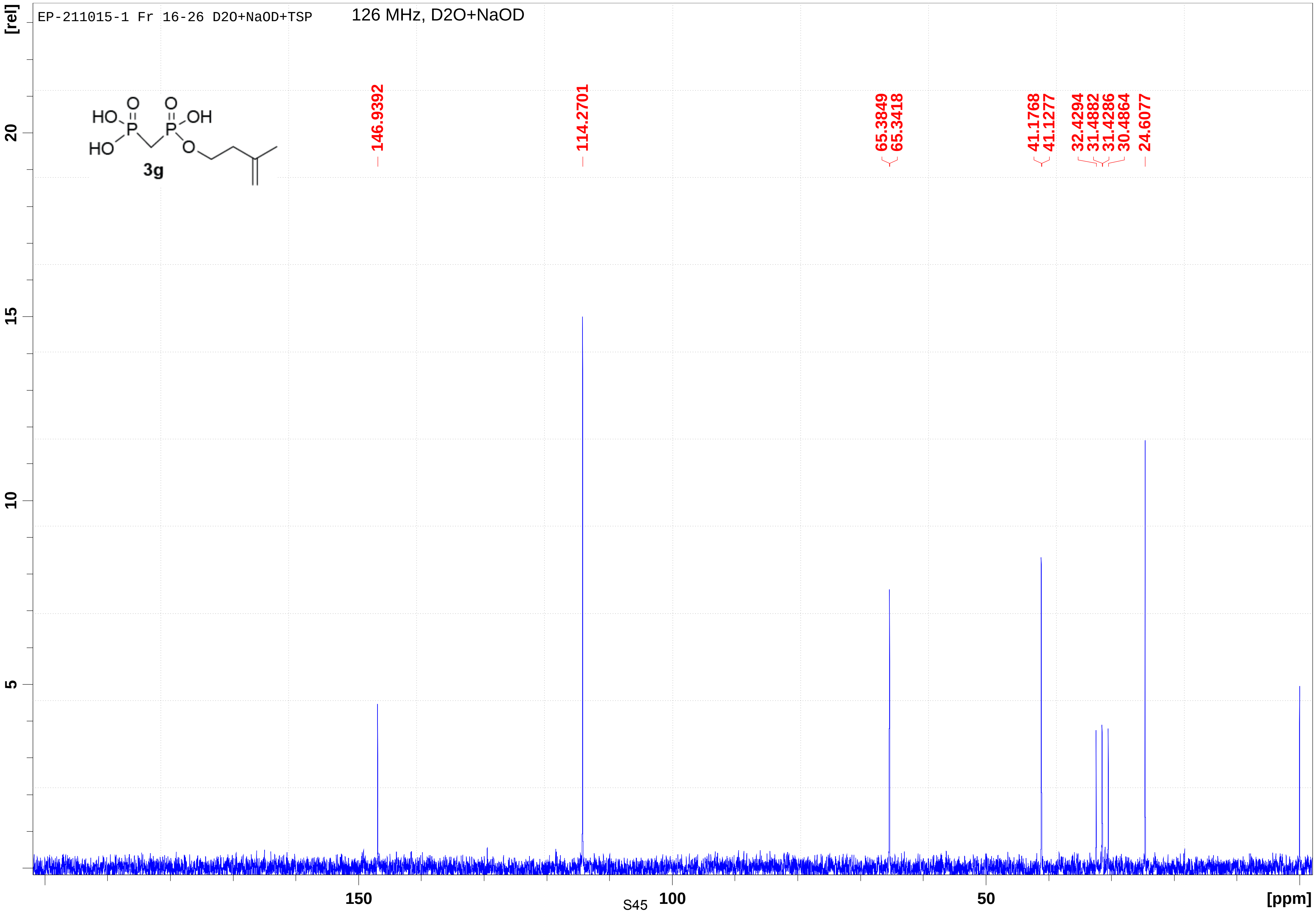

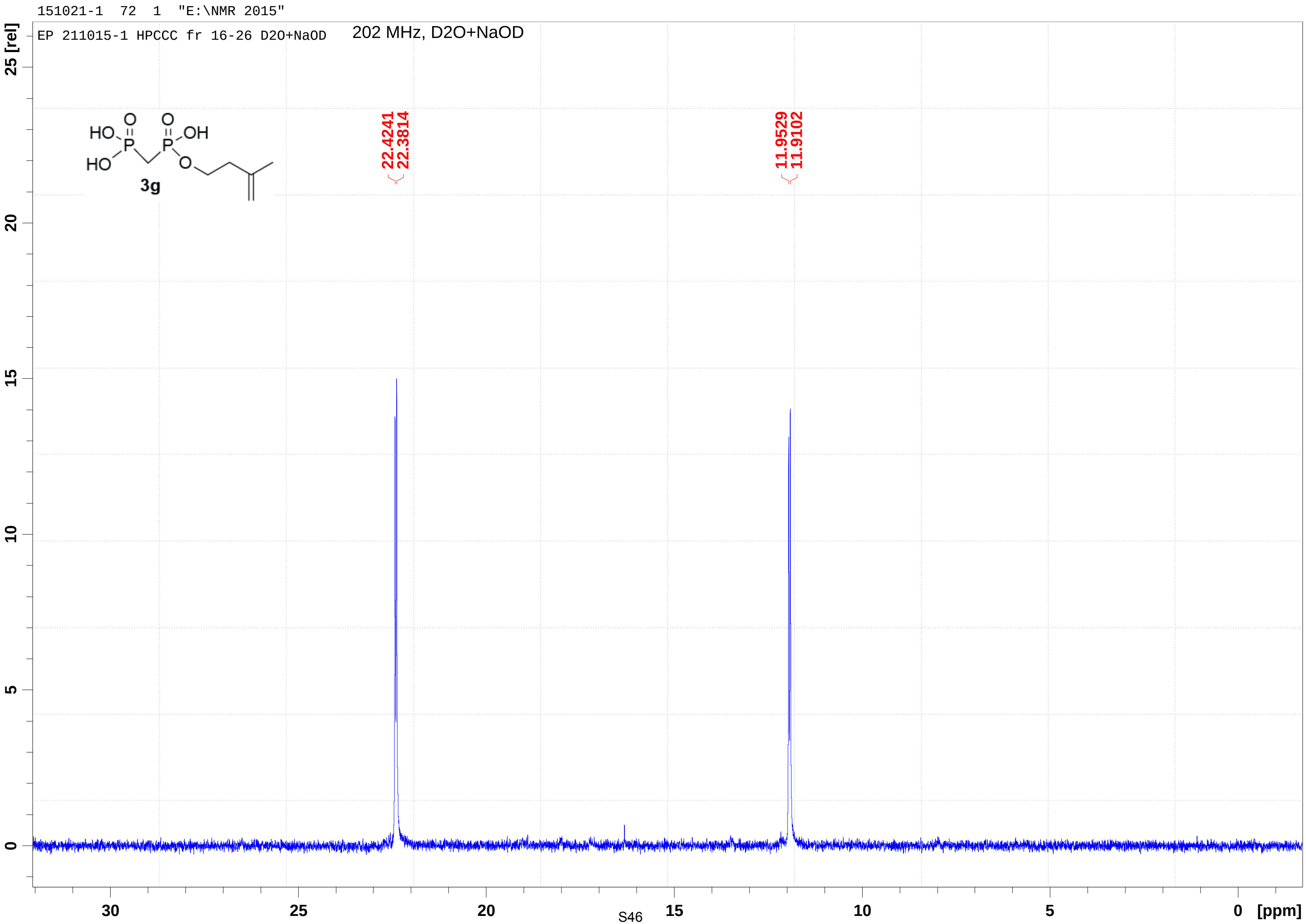
151021-1 72 1 "E:\NMR 2015"
EP 211015-1 HPCCC fr 16-26 D2O+NaOD
202 MHz, D2O+NaOD
3g
22.4241
22.3814
11.9529
11.9102
25 [rel]
20
15
10
5
0
30
25
20
15
10
5
0
[ppm]

HPCCC chromatogram from the purification of **3g**

Detection by UV at 200 (red), 220 (blue) and 254 (green) nm.

Fractions 1-8: **3g** and medronic acid

Fractions 9-23: **3g**

Fractions 24-26: **3g** and **3g** with one methyl ester remaining

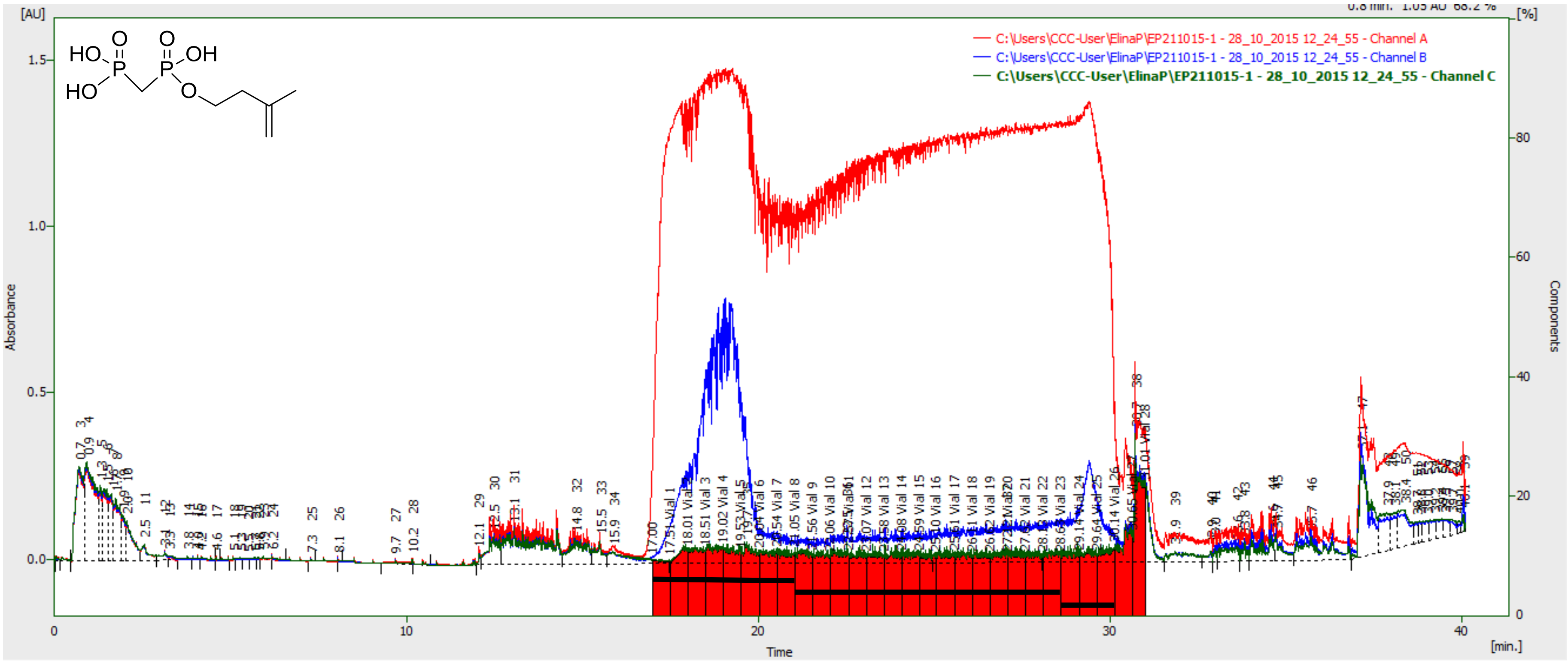

Supplement: File 2 — 1H, 13C, and 31P NMR spectra and an example of HPCCC chromatogram. [file Beilstein_J_Org_Chem-12-2145-s002.pdf]
